# Reactivity and Stereoselectivity in the Inverse-Electron-Demand Diels–Alder Reaction of 1-Aza-1,3-Butadiene

**DOI:** 10.3390/molecules30193861

**Published:** 2025-09-24

**Authors:** Ken Sakata, Yui Go, Takeshi Yoshikawa

**Affiliations:** Department of Physical Chemistry, Faculty of Pharmaceutical Sciences, Toho University, Miyama, Funabashi 274-8510, Chiba, Japan

**Keywords:** inverse-electron-demand Diels–Alder reaction, *endo* selectivity, regioselectivity, DFT calculations

## Abstract

The reactivity and stereoselectivity in the inverse-electron-demand Diels–Alder reaction between 4-methoxycarbonyl-*N*-(phenylsulfonyl)-1-aza-1,3-butadiene and methoxyethene was examined using density functional theory (DFT) calculations at the M06-2X level. The formation of the two bonds in this reaction was calculated to be asynchronous. The formation of the C−C bond occurs first and is driven by electron delocalization from the dienophile to the diene, a process which simultaneously governs the regioselectivity. Moreover, the *endo* selectivity of the reaction was found to arise from non-bonding-orbital interactions, electrostatic attractions, and dispersion interactions. The sulfonyl group attached to the diene influences the selectivity and the reactivity. In contrast, when a methoxycarbonyl group is attached to the diene, it affects the selectivity in a different way depending on the position where it is attached.

## 1. Introduction

The Diels–Alder reaction is one of the most useful reactions in organic synthesis for the synthesis of cyclic compounds [1]. The normal-electron-demand Diels–Alder (NEDDA) reaction, which is a cycloaddition reaction between an electron-rich diene and an electron-deficient dienophile, usually proceeds with *endo* selectivity [2]. Since the proposition of the rule of “maximum accumulation of double bonds” by Alder and Stein [3,4], the *endo* selectivity of the reaction has been discussed from a variety of viewpoints [5,6,7,8,9,10,11,12,13,14,15,16,17,18,19,20,21,22,23,24,25,26,27,28,29]. In particular, secondary orbital interactions (SOIs) between the non-bonded atoms in the diene and dienophile, as proposed by Hoffmann and Woodward, have been widely accepted as the origin of the selectivity [30,31]. The concept of SOI has, in many cases, been used to explain the selectivity of various cycloaddition reactions in terms of the frontier-orbital theory [32]. However, the effect of SOIs turns out to be much weaker than suggested by the frontier-orbital argument when the contribution of other orbitals is taken into consideration [33]. We have previously examined the NEDDA reaction between cyclopentadiene and maleic anhydride using density-functional-theory (DFT) calculations and revealed that the origin of the *endo* selectivity in the reaction arises not from the orbital interactions but from the electrostatic attractions caused by the highly polarized C=O bonds, which play important roles for the reactivity [34]. The effects of Lewis acids on a highly *endo*-selective Lewis-acid-catalyzed NEDDA reaction have also been investigated [35].

**Scheme 1:**
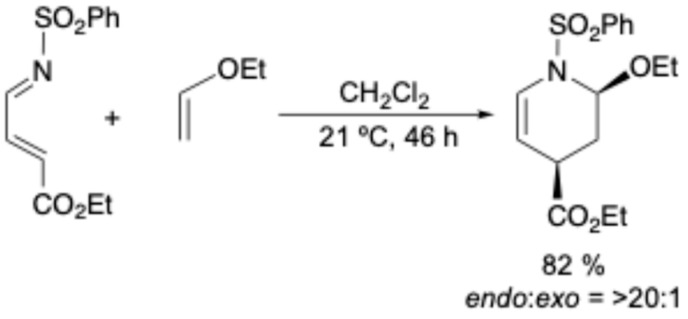
The reaction of 4-ethoxycarbonyl-*N*-(phenylsulfonyl)-1-aza-1,3-butadiene with ethoxyethane reported by Boger and co-workers.

Meanwhile, the inverse-electron-demand Diels–Alder (IEDDA) reaction, which employs an electron-deficient diene and an electron-rich dienophile, has recently emerged as an important tool in both chemical biology and organic synthetic chemistry [36,37,38]. Boger and co-workers have developed IEDDA reactions between *N*-sulfonyl-1-aza-1,3-butadine and electron-rich dienophiles [39,40]. In the reaction of 4-ethoxycarbonyl-*N*-(phenylsulfonyl)-1-aza-1,3-butadiene with ethoxyethane, for example, high regio- and *endo*-selectivity (*endo*:*exo* ratio is >20:1) was reported (Scheme 1) [40].

**Scheme 2:**
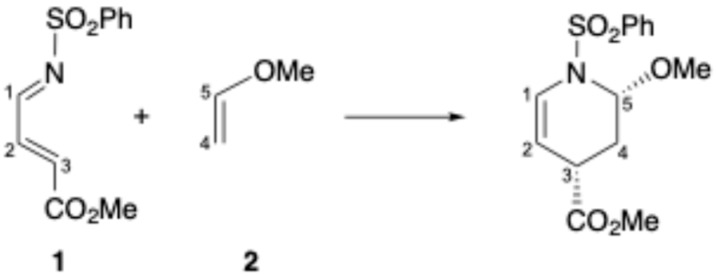
The model reaction examined in the present study. Carbon atoms are numbered as shown.

It has been argued that the *endo* selectivity is a result of not only the interaction between the alkoxy substituent of the dienophile and the carbon atom adjacent to the nitrogen atom in the azadiene, i.e., SOI, but also a result of the *n*-σ* interaction (or anomeric effect) that arises from the interaction between the lone-pair electron of the nitrogen atom and the σ* orbital of the C-O bond. Therefore, it is of great interest to compare the origin of the stereoselectivity in the IEDDA reaction with that of the NEDDA reaction, although some DFT studies on the stereoselectivity in IEDDA reactions were already reported [41,42,43]. In the present study, we have examined in detail a model reaction between 4-methoxycarbonyl-*N*-(phenylsulfonyl)-1-aza-1,3-butadiene (**1**) and methoxyethene (**2**) to clarify the origin of the selectivity of the reaction (Scheme 2).

## 2. Results and Discussion

### 2.1. Transition-State (TS) Structures

**Scheme 3:**
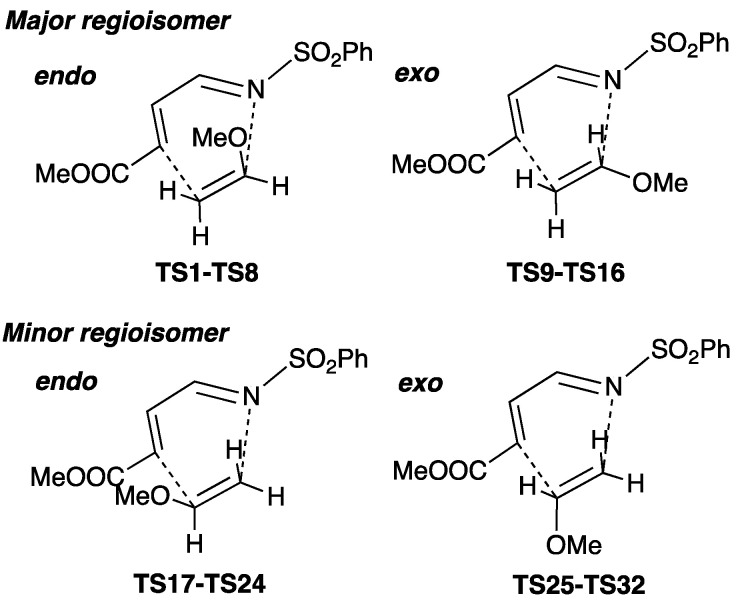
Transition-state structures **TS1**–**TS32** for the cycloaddition reaction between **1** and **2**.

**Figure 1:**
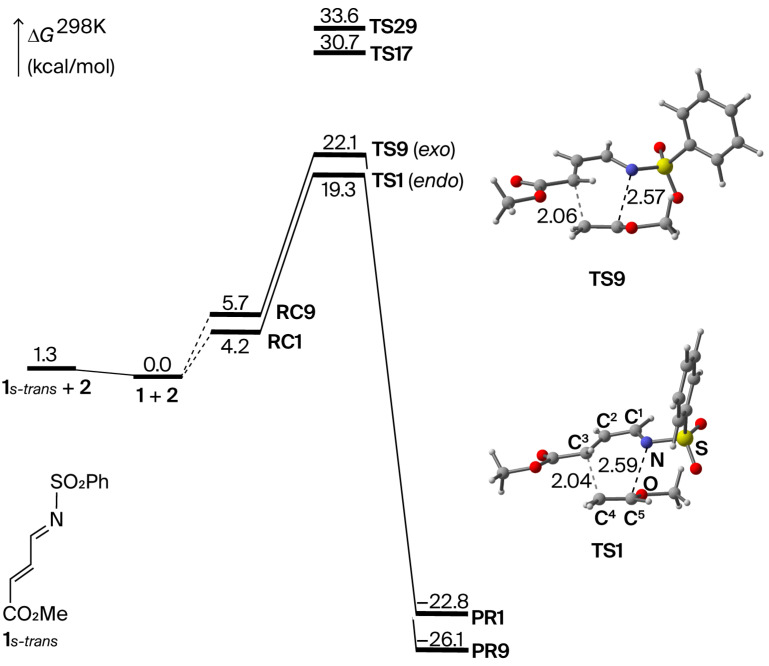
Energy diagram (kcal/mol) and transition-state structures for **TS1** and **TS9** with bond distances in Å.

A variety of transition-state (TS) structures for the cycloaddition (**TS*n***, *n* = 1–32) were explored (Figure S1). TS structures **TS1**–**TS16** lead to the major regioisomer where the nitrogen atom in **1** binds to the carbon atom attached to the methoxy group in **2** (Scheme 3). Conversely, **TS17**–**TS32** give the minor regioisomer where the nitrogen atom in **1** binds to the carbon atom located further away from the methoxy group. Our calculations showed that the latter structures have ca. 10 kcal/mol higher free energies than the former structures, showing that the reaction is highly regioselective (Figure S1 and Table S1). Of the TS structures that lead to the major regioisomer, **TS1**–**TS8** correspond to an *endo* addition, while **TS9**–**TS16** give *exo* adducts. **TS1**, which has the lowest free energy of the *endo* TS structures, was calculated to have 2.8 kcal/mol lower free energy than **TS9**, which has the lowest free energy of the *exo* TSs (Figure 1). A probability distribution of structures of **TS1**–**TS16** provides an *endo*:*exo* ratio of 136:1, showing that the *endo* addition is kinetically preferred over the *exo* addition. These results are consistent with the experimental results reported by Boger and co-workers [40].

### 2.2. Asynchronous Bond Formations and Regioselectivity

Intrinsic reaction coordinate (IRC) [44,45] calculation was performed for **TS1** (Figure S2). The C^3^−C^4^ bond, which is located furthest from the methoxy group, is 2.04 Å in the TS (*s* = 0 amu^1/2^∙bohr), whilst the N−C^5^ bond reaches the same bond length at a much later stage of the reaction (*s* ~ 4.0 amu^1/2^∙bohr). Thus, the C^3^−C^4^ bond is formed much faster than the N−C^5^ bond, demonstrating that the formation of the two bonds is asynchronous. The fragment charges based on a natural population analysis (NPA) [46] show that the C^3^−C^4^ bond formation is brought about primarily by electron delocalization from the dienophile to the diene, while the N−C^5^ bond formation is associated mainly with electron delocalization in the opposite direction (Figure S3). This fragment-charge profile thus indicates the opposite behavior of the NEDDA reactions examined in our previous studies [35,47].

**Figure 2:**
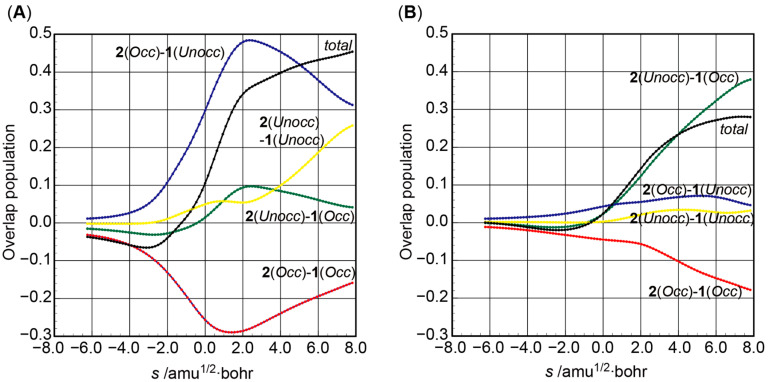
Changes in the Mulliken overlap population for the (**A**) C^3^−C^4^ and (**B**) N−C^5^ bonds along the IRC obtained from **TS1**.

Then, the change in the Mulliken overlap population [48,49,50,51] for the C^3^−C^4^ and N−C^5^ bonds along the IRC was further examined (Figure 2A and Figure 2B, respectively). The population of the C^3^−C^4^ bond is slightly negative when *s* < −1 amu^1/2^∙bohr and after this increases positively. The decomposition in terms of fragment Kohn–Sham orbitals [47,52] showed that the component between the occupied orbitals of the dienophile fragment and unoccupied orbitals of the diene fragment (**2**(*Occ*)−**1**(*Unocc*) in Figure 2A) contributes to the increase in the overlap population. Thus, the electron delocalization from the dienophile to the diene plays an important role in the formation of the C^3^−C^4^ bond. In contrast, the increase in the overlap population between the N and C^5^ atoms (*s* > 0 amu^1/2^∙bohr) depends largely on the contribution of the unoccupied orbitals of the dienophile fragment and the occupied orbitals of the diene fragment (**2**(*Unocc*)−**1**(*Occ*) in Figure 2B). This observation indicates that electron delocalization in the direction opposite to the C^3^−C^4^ bond formation process is essential for the formation of the N−C^5^ bond.

**Figure 3:**
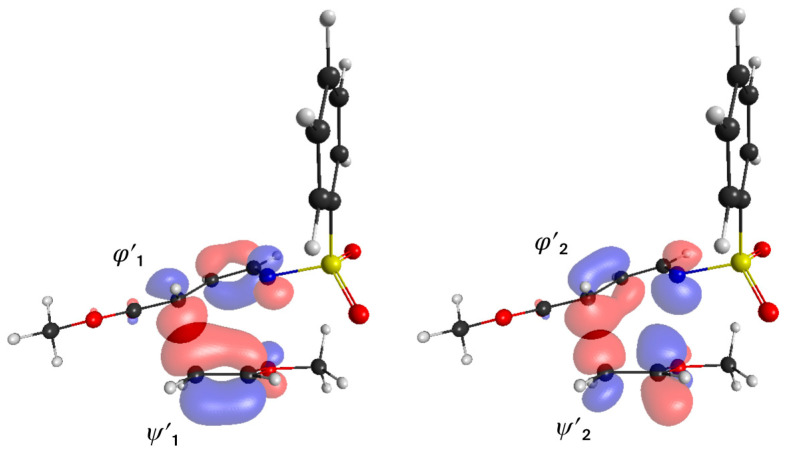
Pairs of interaction frontier orbitals (IFOs) (*ψ*′_1_; *φ*′_1_) and (*ψ*′_2_; *φ*′_2_) in **TS1**.

Subsequently, orbital interactions were analyzed in terms of the interaction frontier orbitals (IFOs) [53,54]. One pair of orbitals, (*ψ*′_1_; *φ*′_1_), is mainly responsible for the electron delocalization from the dienophile fragment to the diene fragment in **TS1** (Figure 3). The orbital *ψ*′_1_, obtained from the linear combination of the occupied Kohn–Sham orbitals of the dienophile fragment, consists of the π bonding orbital of the C=C bond of the dienophile and the amplitude of the C^4^ atom is larger than that of the C^5^ atom. The orbital *φ*′_1_, which consists of the unoccupied orbitals of the diene fragment, is similar to the LUMO of butadiene and the amplitude of the C^3^ atom is slightly larger than that of the N atom. Orbitals *ψ*′_1_ and *φ*′_1_ have energies of −7.62 eV and −2.05 eV, respectively. The interaction between these two orbitals is important for the C^3^−C^4^ bond formation process.

Another pair of orbitals, (*ψ*′_2_; *φ*′_2_), governs the electron delocalization from the diene to the dienophile (Figure 3). The orbital *ψ*′_2_ is the unoccupied π orbital of the dienophile, while the orbital *φ*′_2_ is the occupied π orbital of the diene. Orbitals *ψ*′_2_ and *φ*′_2_ have energies of +3.85 eV and −9.90 eV, respectively. This pair is responsible for the N−C^5^ bond formation process, although the interaction is weaker than that of the (*ψ*′_1_; *φ*′_1_) pair of orbitals due to the larger energy difference between the *ψ*′_2_ and *φ*′_2_ orbitals. We also investigated other pairs of orbitals but did not find any that corresponded to an *n*-σ* interaction.

The regioselectivity in this reaction is closely related to the asynchronous bond-formation process. The electron-withdrawing SO_2_Ph group in the diene fragment both lowers the energy levels of the occupied and unoccupied π orbitals and prompts the polarization of the π orbitals (Figure S4). The SO_2_Ph group enhances the overlap repulsion at the N atom, while the repulsion at the C^3^ atom is diminished. At the same time, the electron-accepting ability of the *p*π orbital at the C^3^ atom increases. On the other hand, the electron-donating ability of the *p*π orbital at the C^4^ atom in the dienophile fragment is strengthened by the methoxy group [2]. Thus, the combination of the C^3^ atom and the C^4^ atom favors electron delocalization from the dienophile fragment to the diene fragment, controlling the regioselectivity of the reaction.

### 2.3. Stereoselectivity

**Scheme 4:**
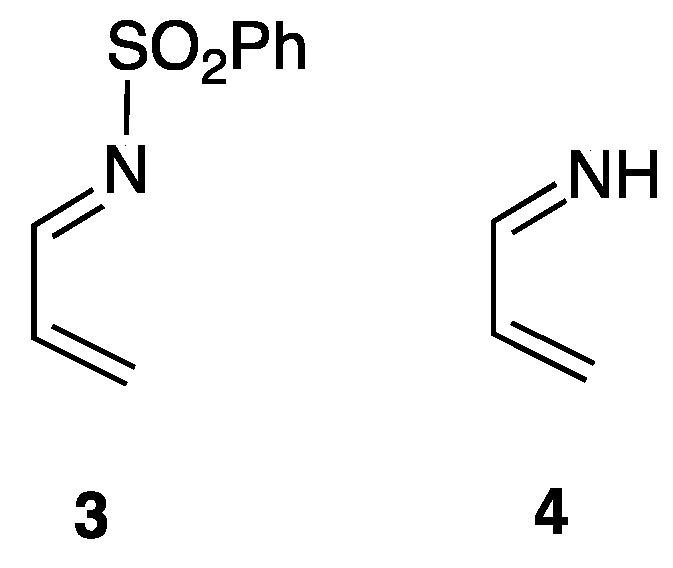
*N*-(phenylsulfonyl)-1-aza-1,3-butadiene (**3**) and 1-aza-1,3-butadiene (**4**).

In the reaction involving *N*-(phenylsulfonyl)-1-aza-1,3-butadiene (**3**) (Scheme 4), the relative free energies of the *endo* and *exo* TSs, **TS1‴** and **TS9‴**, were calculated to be 17.9 and 20.3 kcal/mol, respectively. Thus, the free energy of the *endo* TS **TS1‴** is by 2.4 kcal/mol lower than that of the *exo* TS **TS9‴**, indicating that the effect that the CO_2_Me group on the C^3^ atom has on the *endo* selectivity is relatively small (Figure S5a). In contrast, the relative free energies of the *endo* and *exo* TSs, **TS1⁗** and **TS9⁗**, were found to be 30.3 and 29.8 kcal/mol, respectively, in the reaction involving 1-aza-1,3-butadiene (**4**) (Figure S5b). It shows that the energy of the *endo* structure, **TS1⁗**, is 0.5 kcal/mol higher than that of the *exo* structure, **TS9⁗**. These results demonstrate that, in addition to resulting in higher reactivity, the SO_2_Ph group also dictates the stereochemical outcome of the reaction. The results of an energy decomposition analysis (EDA) [55] suggested that, although the group directly interacts with the dienophile, the SO_2_Ph group facilitates the interaction between the diene group and the dienophile (Figure S6).

As shown in Figure 3, the small *p*π amplitude at the oxygen atom of the methoxy group in *ψ*′_1_ can be *in-phase* with the amplitudes around the C^1^−C^2^ bond in *φ*′_1_ when the *p*π amplitude of the C^4^ atom in *ψ*′_1_ overlaps with the same phase of the C^3^ atom in *φ*′_1_. This may suggest the presence of a non-bonding orbital interaction between the oxygen atom in *ψ*′_1_ and the C^1^ or C^2^ atom in the *endo* TS (in **TS1**, the distances between the oxygen atom and the C^1^ atom and between the oxygen atom and the C^2^ atom are 2.90 and 3.03 Å, respectively). Thus, we examined the overlap population between the O and C^1^ atoms and between the O and C^2^ atoms in **TS1** (Figure S7a,b). The overlap population between the O and C^1^ atoms has a small positive value of +0.020. Here, the contribution between the occupied orbitals of the dienophile fragment and the unoccupied orbitals of the diene fragment has a positive value, +0.035, while the contribution between the unoccupied orbitals of the dienophile fragment and the occupied orbitals of the diene fragment has a negative value of −0.010. The contribution in between the occupied orbitals also has a negative value of −0.008. On the other hand, the overlap population between the O and C^2^ atoms also has a positive value of +0.042. This is because the contribution between the occupied orbitals of the dienophile and the unoccupied orbitals of the diene is +0.037, while the contribution between the unoccupied orbitals of the dienophile and the occupied orbitals of the diene is +0.009. Thus, the non-bonding orbital interaction between the methoxy oxygen atom and C^1^ or C^2^ atom exists, but the value is relatively small.

In the case where the SO_2_Ph group is removed from the diene, the overlap population between the O and C^1^ atoms decreases (Figure S6). The value of the contribution between the occupied orbitals of the diene and the unoccupied orbitals of the dienophile becomes negative, making the total overlap between these atoms smaller. In contrast, the overlap population between the O and C^2^ atoms increases because of the increase in the contribution value between the occupied orbitals of the diene and the unoccupied orbitals of the dienophile. The introduction of an SO_2_Ph group into the diene changes the non-bonding orbital interaction by diminishing the electron delocalization from the diene to the dienophile.

The polarization of the π orbitals induced by the SO_2_Ph group of the diene causes a decrease in the electron population at the C^1^ atom as well as an increase in the electron-accepting ability of the *p*π orbital at the C^3^ atom (Figure S8). This change affects the weak attractive electrostatic interaction between the non-bonded atoms. The electrostatic potential (ESP) maps on the isodensity surfaces of **1** and **2** (Figure S9) show that the methoxy oxygen atom is responsible for the negatively charged region in **2** (the atomic charge calculated by NPA is −0.53), while the C^1^−C^2^ bond is the location of the positively charged region in **1**. The attraction between these two regions increases in the *endo* TS. Thus, the electrostatic attraction between the methoxy oxygen atom and the C^1^−C^2^ bond contributes to the *endo* preference. Furthermore, the results of the EDA also suggested that the dispersion energy has an effect in this context. A non-covalent interaction (NCI) plot for **TS1** shows interactions not only between the dienophile and the SO_2_Ph group in the diene but also between the non-bonded atoms (Figure S10).

### 2.4. Effect of the Position of the Methoxycarbonyl Group on the Reactivity and Selectivity

**Scheme 5:**
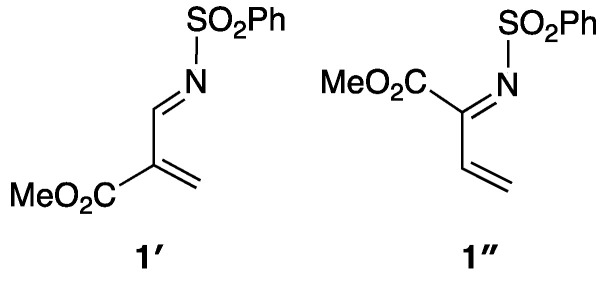
3-Methoxycarbonyl-*N*-(phenylsulfonyl)-1-aza-1,3-butadiene (**1′**) and 2-methoxycarbonyl-*N*-(phenylsulfonyl)-1-aza-1,3-butadiene (**1″**).

**Figure 4:**
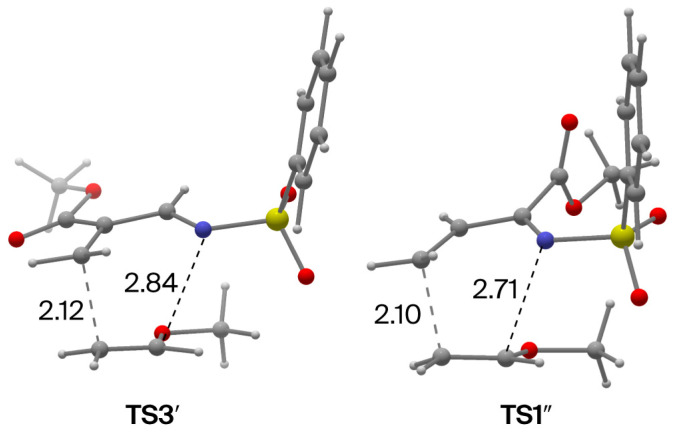
TS structures **TS3′** and **TS1″** with bond distances in Å.

In 1, the electron-withdrawing CO_2_Me group is attached to the C^3^ atom. Boger and co-workers have reported that the introduction of the electron-withdrawing group at the C^2^ atom enhances the reactivity [40]. We therefore investigated the effect of the position of the methoxycarbonyl group on both the reactivity and the *endo* selectivity (Scheme 5). In the case of **1′**, **TS3′** has the lowest free energy of the *endo* attack TSs studied here (Figure 4 and Figure S11). The free energy of **TS3′** relative to the initial state (**1′** + **2**) (11.1 kcal/mol) is much lower than that of **TS1** in the reaction of **1** (19.3 kcal/mol). This result suggests that the reactivity of **1′** is much higher than that of 1. In addition, **TS3′** has 3.3 kcal/mol lower free energy than the lowest energy *exo* transition state (**TS11′**). The *endo* preference in the reaction of **1′** is slightly higher than that in the reaction of **1** (the probability distributions of the structures **TS1′**–**TS16′** afford an *endo*:*exo* ratio of 236:1).

In the case of **1″**, the free energies of the lowest energy *endo* and *exo* TSs relative to the initial state (**1″** +**2**), i.e., **TS1″** and **TS9″**, are 15.1 and 18.9 kcal/mol, respectively (the energy difference is 3.8 kcal/mol; Figure 4 and Figure S12). Thus, the *endo* preference in this case (*endo*:*exo* = 707:1) was found to be higher than that of **1** and **1′**. This result is in agreement with the experimentally observed *endo*:*exo* ratio (>20:1) in the reaction between 4-phenyl-2-ethoxycarbonyl-N-(phenylsulfonyl)-1-aza-1,3-butadiene and ethoxyethane [40].

As has already been shown, the electron-accepting ability at the C^3^ atom in the diene plays an important role in the reaction. To investigate further, we used the 2 *p*π orbital at the C^3^ atom as the reference orbital, *δ_r_*, and then projected this reference orbital onto the unoccupied orbital spaces of **3**, **4**, **1**, **1′**, and **1″**. The orbital has energy expectation values, *λ_unoc_*(*δ_r_*), of +0.67 and +1.53 eV for **3** and **4**, respectively, showing that the SO_2_Ph group greatly strengthens the electrophilicity of the C^3^ atom. Certainly, the reactivity of **3** is much higher than that of **4** in terms of activation energy. Moreover, the *λ_unoc_*(*δ_r_*) values of **1**, **1′**, and **1″** were estimated to be +1.14, −0.05, +0.55 eV, respectively. The attachment of a CO_2_Me group to the C^3^ atom weakens the electrophilicity of the C^3^ atom, while when this group is attached to the C^2^ atom it strengthens the electrophilicity of the C^3^ atom. These results show that the electron-accepting ability of the dienes increases in the order **1′** > **1″** > **1**, which is in agreement with their order of reactivity in the IEDDA reaction.

With regard to the *endo* selectivity in the reaction of **1′**, the positively charged carbon atom in the CO_2_Me group attached to the C^2^ atom (+0.80 for NPA atomic charge) increases the electrostatic attractive interaction with the methoxy oxygen atom. However, the slightly larger deformation of the dienophile in the *endo* structure compensates for the larger interaction energy. Consequently, the *endo* preference slightly increases in comparison to that in the reaction of **1**. On the other hand, the larger deformation of the diene in the *exo* structure increases the *endo* preference in the case of **1″**, because the CO_2_Me group, which is roughly orthogonal to the diene backbone, causes the geometrical position of the SO_2_Ph group to change slightly (Table S2 and Figure S13). Thus, the influence that a CO_2_Me group attached to the C^1^ atom has on the selectivity is different from that when it is attached to the C^2^ atom.

## 3. Computational Details

DFT calculations were carried out using the *Gaussian16* [56] and *GAMESS* [57] packages. Geometry optimizations and analytical vibrational-frequency analyses were performed using restricted Kohn–Sham DFT calculations [58,59] based on the M06-2X functional [60,61,62,63]. For the numerical integration, a larger grid (*superfinegrid*) was used in the *Gaussian16* program [56]. Pople’s 6-311G(*d*,*p*) basis set was used for the Gaussian basis functions (6*d* polarization functions) [64].

## 4. Conclusions

The inverse-electron-demand Diels–Alder reaction between 4-methoxycarbonyl-*N*-(phenylsulfonyl)-1-aza-1,3-butadiene and methoxyethene was examined using DFT calculations at the M06-2X level. The formation of the two bonds in this reaction is asynchronous, i.e., the formation of the C−C bond proceeds that of the N−C bond. The formation of the C−C bond is driven by electron delocalization from the dienophile to the diene, indicating that the reaction is an inverse-electron-demand reaction. The *endo* structure is preferred over the *exo* structure due to a weak non-bonding orbital interaction, electrostatic attractions, and dispersion interactions. The presence of an electron-withdrawing SO_2_Ph group in the diene results in higher reactivity and *endo* selectivity. The presence of a CO_2_Me group in the diene affects the selectivity in a different way depending on the position where it is attached.

## Data Availability

All related data and methods are presented in this paper and the Supplementary Materials.

## Supplementary Materials

The following supporting information can be downloaded at https://www.mdpi.com/article/10.3390/molecules30193861/s1. Table S1: Total electronic energy (*E*) and Gibbs free energy (*G*); Table S2: EDA for the reactions of **3**, **1**, **1′**, and **1″** with **2**; Figure S1: Transition state structures of **TS1**–**TS32**; Figure S2: Change in bond distances along the IRC of **TS1**; Figure S3: The fragment charge based on natural population analysis along the IRC of **TS1**; Figure S4: Natural population atomic charges in 1-aza-1,3-butadiene and *N*-(phenylsulfonyl)-1-aza-1,3-butadiene; Figure S5: Transition state structures for (a) the reaction between **3** and **2** and (b) the reaction between **4** and **2**; Figure S6: Energy decomposition analysis and overlap populations for **TS1‴** and **TS9‴**; Figure S7: Change in the Mulliken overlap populations (a) between O and C^1^ atoms and (b) between O and C^2^ atoms along the IRC of **TS1**; Figure S8: Natural population atomic charges in **1** and **2**; Figure S9: The electrostatic potential maps on the isodensity (0.0004 e/au^3^) surfaces of **1** and **2**; Figure S10: NCI plot for **TS1**; Figure S11: Transition state structures of **TS1′**–**TS32′**; Figure S12: Transition state structures of **TS1″**–**TS32″**; Figure S13: Dihedral angle of C(Ph gorup)−S−N−C^1^, *ϕ* (degree); Cartesian coordinates of stationary points.
