# Supplementary material for: Reactivity and Stereoselectivity in the Inverse-Electron-Demand Diels–Alder Reaction of 1-Aza-1,3-Butadiene
Source: Molecules. 2025 Sep 24;30(19):3861. doi: 10.3390/molecules30193861 (PMC12525806; doi:10.3390/molecules30193861)
Supplement: Supplementary file 1 [file molecules-30-03861-s001.zip › molecules-3880408-supplementary.pdf]

Supplementary Material

Reactivity and Stereoselectivity in Inverse-Electron-Demand Diels−Alder Reaction of 1-Aza-1,3-Butadiene

Ken Sakata*, Yui Go, and Takeshi Yoshikawa

Faculty of Pharmaceutical Sciences, Toho University, Miyama, Funabashi, Chiba 274-8510, Japan

Contents

Total electronic energy (*E*) and Gibbs free energy (*G*) (**Table S1**) S2
Energy decomposition analysis (EDA) for the reactions of **3**, **1**, **1′**, and **1″** with **2** (**Table S2**) S4
Transition state structures of **TS1-TS32** (**Figure S1**) S5
Change in bond distances along the IRC of **TS1** (**Figure S2**) S7
The fragment charge based on natural population analysis along the IRC of **TS1** (**Figure S3**) S7
Natural population atomic charges in 1-aza-1,3-butadiene and *N*-(phenylsulfonyl)-1-aza-1,3-butadiene (**Figure S4**) S8
Transition state structures for (a) the reaction between **3** and **2** and (b) the reaction between **4** and **2** (**Figure S5**) S8
Energy decomposition analysis and overlap populations for **TS1‴** and **TS9‴** (**Figure S6**) S9
Change in the Mulliken overlap populations (a) between O and C^1 atoms and (b) between O and C^2 atoms along the IRC of **TS1** (**Figure S7**) S10
Natural population atomic charges in **1** and **2** (**Figure S8**) S11
The electrostatic potential maps on the isodensity (0.0004 e/au^3) surfaces of **1** and **2** (**Figure S9**) S11
NCI plot for **TS1** (**Figure S10**) S12
Transition state structures of **TS1′-TS32′** (**Figure S11**) S13
Transition state structures of **TS1″-TS32″** **(Figure S12)** S15
Dihedral angle of C(Ph gorup)−S−N−C^1, ϕ (degree). (**Figure S13**) S17
Cartesian coordinates of stationary points at the M06-2X/6-311G(d,p)[6D] level of theory S18

Table S1. Total Electronic Energy E and Gibbs Free Energy G at 298 K (au)

	E	G
**1**	−1179.424718	−1179.260405
**$1_{s\text{-}trans}$**	−1179.422193	−1179.258343
**2**	−193.073180	−193.016467
**TS1**	−1372.494409	−1372.246147
**TS2**	−1372.486147	−1372.237624
**TS3**	−1372.493692	−1372.245481
**TS4**	−1372.484993	−1372.236231
**TS5**	−1372.487293	−1372.238315
**TS6**	−1372.483929	−1372.234406
**TS7**	−1372.485804	−1372.236776
**TS8**	−1372.482042	−1372.232521
**TS9**	−1372.489925	−1372.241613
**TS10**	−1372.487260	−1372.239155
**TS11**	−1372.487424	−1372.240257
**TS12**	−1372.486005	−1372.238024
**TS13**	−1372.484805	−1372.236213
**TS14**	−1372.478872	−1372.231891
**TS15**	−1372.484970	−1372.236355
**TS16**	−1372.477907	−1372.230512
**TS17**	−1372.475425	−1372.227889
**TS18**	−1372.472132	−1372.223837
**TS19**	−1372.474939	−1372.226930
**TS20**	−1372.471463	−1372.222526
**TS21**	−1372.472876	−1372.224822
**TS22**	−1372.470103	−1372.220227
**TS23**	−1372.472219	−1372.224432
**TS24**	−1372.469206	−1372.219381
**TS25**	−1372.469115	−1372.222036
**TS26**	−1372.465331	−1372.217468
**TS27**	−1372.467378	−1372.221631
**TS28**	−1372.463296	−1372.216221
**TS29**	−1372.470940	−1372.223384
**TS30**	−1372.468514	−1372.219898
**TS31**	−1372.469776	−1372.222017
**TS32**	−1372.466883	−1372.218134
**RC1**	−1372.512239	−1372.270234
**PR1**	−1372.568591	−1372.313130
**RC9**	−1372.510138	−1372.267794
**PR9**	−1372.573492	−1372.318431

**1′**	–1179.422989	–1179.259083
**TS1′**	–1372.504672	–1372.257142
**TS2′**	–1372.499016	–1372.250862
**TS3′**	–1372.505294	–1372.257919
**TS4′**	–1372.498793	–1372.250898
**TS5′**	–1372.497668	–1372.249130
**TS6′**	–1372.494926	–1372.245921
**TS7′**	–1372.499557	–1372.251110
**TS8′**	–1372.496183	–1372.247208
**TS9′**	–1372.499331	–1372.251603
**TS10′**	–1372.498217	–1372.250854
**TS11′**	–1372.500009	–1372.252681
**TS12′**	–1372.497914	–1372.250634
**TS13′**	–1372.492849	–1372.245451
**TS14′**	–1372.488491	–1372.241944
**TS15′**	–1372.494151	–1372.246459
**TS16′**	–1372.488826	–1372.242468
**TS17′**	–1372.478391	–1372.231404
**TS18′**	–1372.475464	–1372.227742
**TS19′**	–1372.478391	–1372.231404
**TS20′**	–1372.476704	–1372.228865
**TS21′**	–1372.477574	–1372.230203
**TS22′**	–1372.475270	–1372.225629
**TS23′**	–1372.477469	–1372.229866
**TS24′**	–1372.474414	–1372.224925
**TS25′**	–1372.472991	–1372.226967
**TS26′**	–1372.469375	–1372.222637
**TS27′**	–1372.474130	–1372.228238
**TS28′**	–1372.469783	–1372.223263
**TS29′**	–1372.475516	–1372.227570
**TS30′**	–1372.473095	–1372.224256
**TS31′**	–1372.476080	–1372.228223
**TS32′**	–1372.473017	–1372.224312
**1″**	–1179.411092	–1179.246889
**TS1″**	–1372.487638	–1372.239273
**TS2″**	–1372.480428	–1372.231999
**TS3″**	–1372.488451	–1372.239241
**TS4″**	–1372.480968	–1372.232820
**TS5″**	–1372.478783	–1372.230111
**TS6″**	–1372.470087	–1372.221080

**TS7″**	–1372.480288	–1372.231329
**TS8″**	–1372.476075	–1372.225255
**TS9″**	–1372.481647	–1372.233170
**TS10″**	–1372.477310	–1372.229377
**TS11″**	–1372.480417	–1372.232908
**TS12″**	–1372.477048	–1372.229587
**TS13″**	–1372.472777	–1372.225328
**TS14″**	–1372.470296	–1372.222329
**TS15″**	–1372.472597	–1372.225007
**TS16″**	–1372.469393	–1372.221927
**TS17″**	–1372.462722	–1372.214852
**TS18″**	–1372.460574	–1372.213020
**TS19″**	–1372.459998	–1372.212477
**TS20″**	–1372.459386	–1372.211321
**TS21″**	–1372.463992	–1372.215035
**TS22″**	–1372.460725	–1372.211968
**TS23″**	–1372.463555	–1372.214296
**TS24″**	–1372.461767	–1372.211782
**TS25″**	–1372.459481	–1372.212688
**TS26″**	–1372.452961	–1372.205953
**TS27″**	–1372.458173	–1372.211266
**TS28″**	–1372.453912	–1372.207908
**TS29″**	–1372.463116	–1372.214218
**TS30″**	–1372.457880	–1372.209513
**TS31″**	–1372.461883	–1372.213186
**TS32″**	–1372.463116	–1372.214000

Table S2. Energy decomposition analysis (EDA) for the reactions of **3**, **1**, **1′**, and **1″** with **2** (kcal/mol).

	**3**			**1**			**1′**			**1″**		
	endo	*exo*	*Δ*	*endo*	*exo*	*Δ*	*endo*	*exo*	*Δ*	*endo*	*exo*	*Δ*
DEF_A	13.9	14.3	*−0.4*	16.9	17.0	*−0.1*	10.0	10.0	*0.0*	12.1	13.2	*−1.1*
DEF_B	7.8	7.0	*0.8*	8.9	8.2	*0.7*	6.0	4.5	*1.5*	6.9	6.7	*0.2*
DEF	21.7	21.3	*0.4*	25.8	25.2	*0.6*	16.0	14.5	*1.5*	19.0	19.9	*−0.9*
ES	−52.0	−51.1	*−0.9*	−58.5	−56.6	*−1.9*	−44.9	−39.4	*−5.5*	−47.7	−48.8	*1.1*
EX	−73.5	−72.9	*−0.6*	−81.1	−79.4	*−1.7*	−61.4	−55.5	*−5.9*	−66.9	−69.3	*2.4*
REP	195.7	195.1	*0.6*	218.0	214.1	*3.9*	165.7	150.7	*15.0*	180.2	185.9	*−5.7*
POL	−57.6	−56.7	*−0.9*	−64.8	−62.3	*−2.5*	−48.3	−42.7	*−5.7*	−52.1	−54.3	*2.2*
DISP	−33.6	−32.6	*−1.0*	−37.2	−36.0	*−1.2*	−32.9	−30.0	*−2.8*	−34.6	−31.8	*−2.8*
INT	−21.0	−18.2	*−2.8*	−23.6	−20.2	*−3.4*	−21.7	−16.9	*−4.9*	−21.2	−18.3	*−2.9*
total	0.7	3.1	*−2.4*	2.2	5.0	*−2.8*	−5.7	−2.4	*−3.3*	−2.1	1.7	*−3.8*

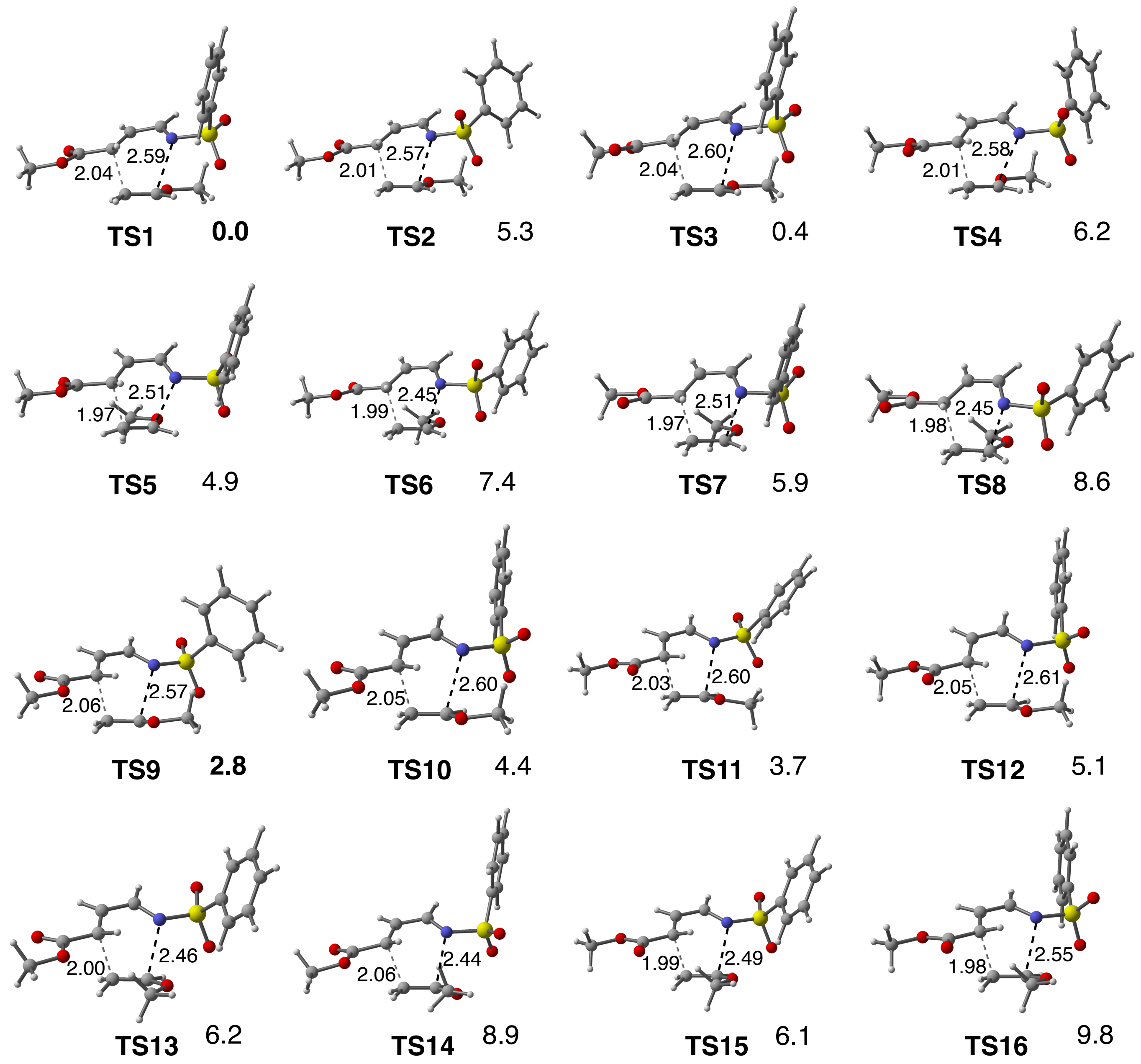

Figure S1. Transition state structures of **TS1-TS32**. Gibbs free energies relative to **TS1** are shown in kcal/mol. Distances are shown in Å.

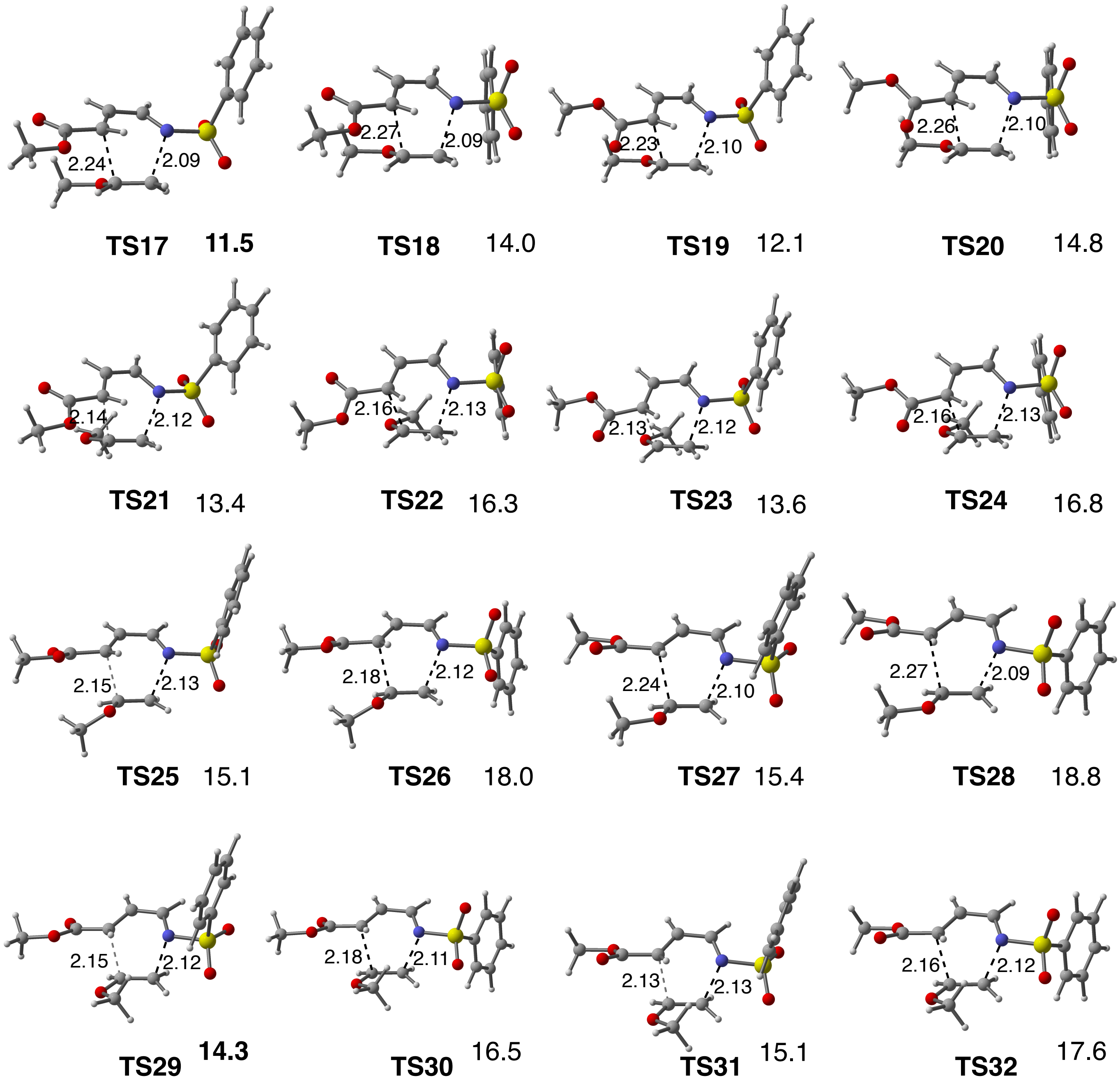

Figure S1. (*Continued.*)

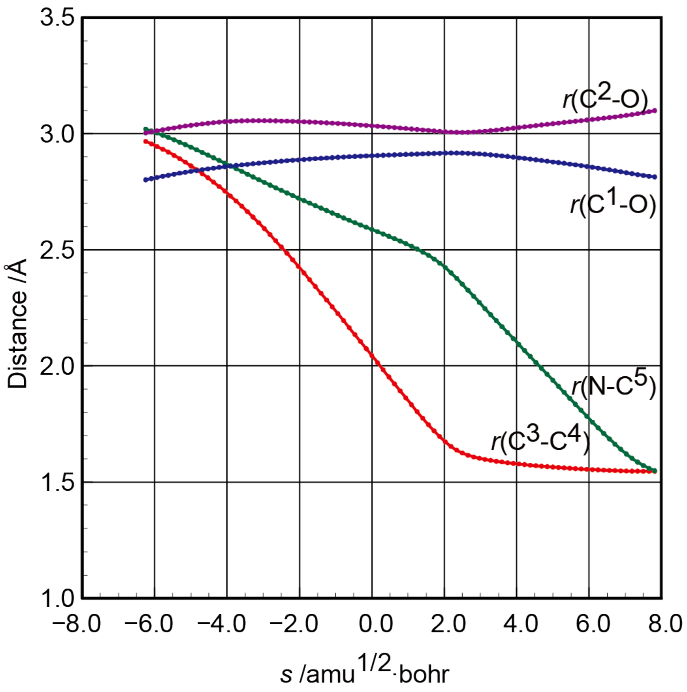

Figure S2. Change in bond distances along the IRC of **TS1**.

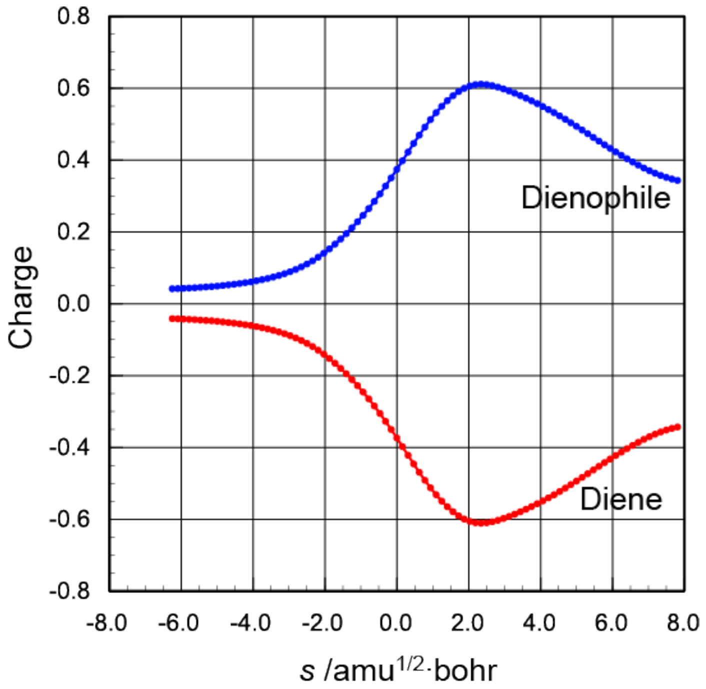

Figure S3. The fragment charge based on natural population analysis (NPA) along the IRC of **TS1**.

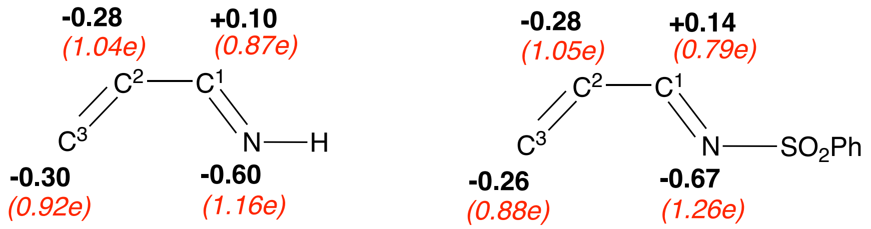

Figure S4. Natural population atomic charges in 1-aza-1,3-butadiene and *N*-(phenylsulfonyl)-1-aza-1,3-butadiene in which 1-aza-1,3-butadiene part was fixed on a plane surface. Electron populations of pπ orbital are shown in parenthesis.

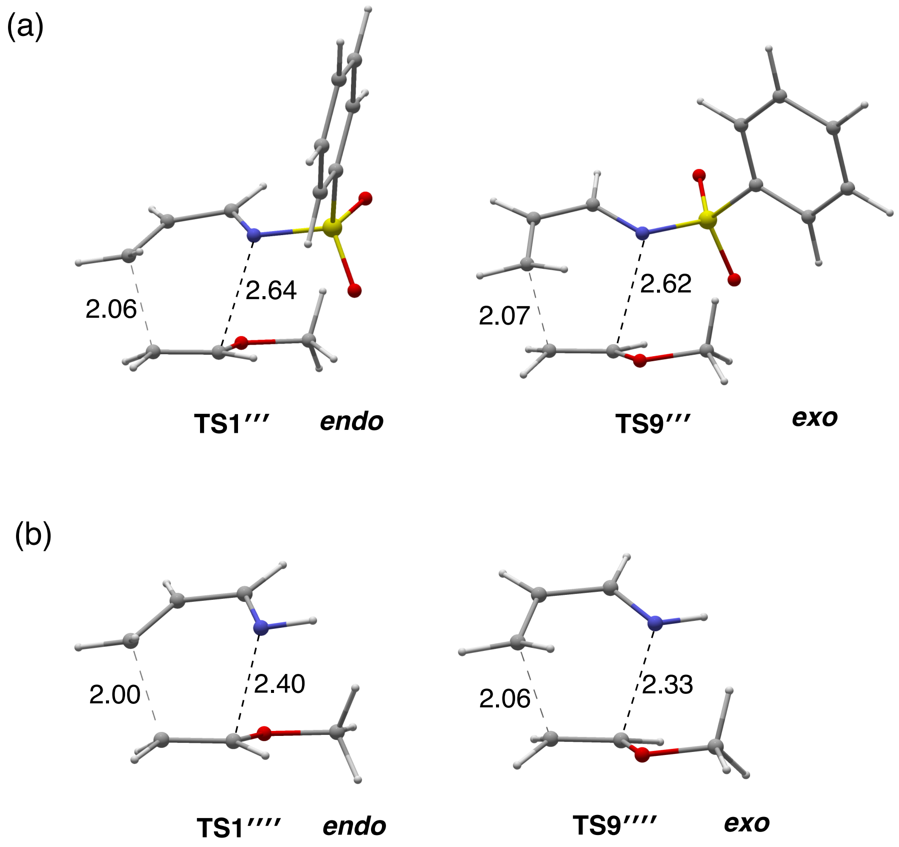

Figure S5. Transition state structures for (a) the reaction between **3** and **2**, **TS1‴** and **TS9‴**, and (b) the reaction between **4** and **2**, **TS1⁗** and **TS9⁗**. Distances are shown in Å.

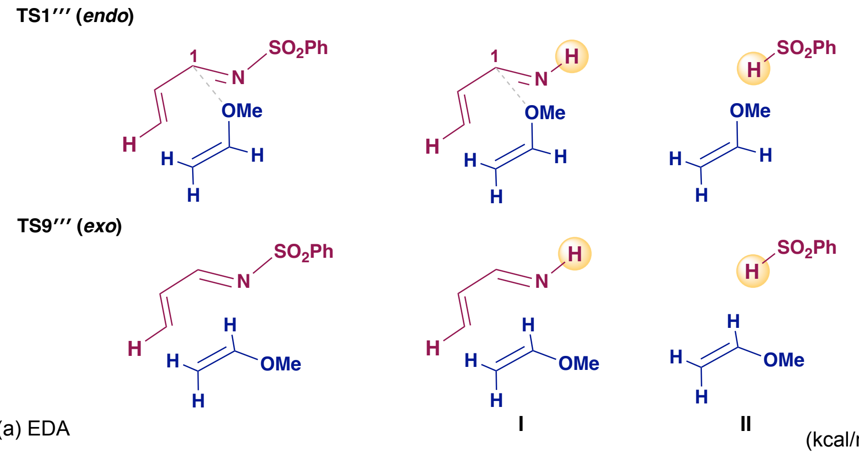

(a) EDA

(kcal/mol)

				I			II		
	endo	*exo*	*Δ*	*endo*	*exo*	*Δ*	*endo*	*exo*	*Δ*
ES	−51.98	−51.12	*−0.86*	−45.67	−46.34	*0.67*	−6.24	−5.22	*−1.02*
EX	−73.50	−72.94	*−0.56*	−72.55	−71.80	*−0.75*	−4.00	−4.57	*0.57*
REP	195.69	195.06	*0.63*	188.32	186.34	*1.98*	15.37	17.49	*−2.12*
POL	−57.61	−56.65	*−0.96*	−50.23	−49.86	*−0.37*	−2.57	−3.00	*0.43*
DISP	−33.58	−32.60	*−0.98*	−26.88	−24.72	*−2.16*	−9.07	−10.42	*1.35*
total	−20.98	−18.24	*−2.74*	−7.00	−6.38	*−0.62*	−6.51	−5.71	*−0.80*

(b) Overlap population between C^1 and O atoms in the *endo* structure

Occ-Occ	−0.010	−0.011
Occ-Unocc	0.035	0.033
Unocc-Occ	−0.008	−0.019
Unocc-Unocc	0.002	0.006
total	0.020	0.010

(c) Overlap population between C^2 and O atoms in the *endo* structure

Occ-Occ	−0.004	−0.002
Occ-Unocc	0.037	0.033
Unocc-Occ	0.009	0.019
Unocc-Unocc	−0.000	0.001
total	0.042	0.050

Figure S6. (a) Energy decomposition analysis and (b, c) overlap populations for **TS1'''** and **TS9'''**. In model **I**, SO_2Ph group was replaced to the hydrogen atom with the geometry of the remaining atoms frozen to the same as that in **TS1'''** or **TS9'''**. In model **II**, 1-aza-1,3-butadine was replaced to the hydrogen atom.

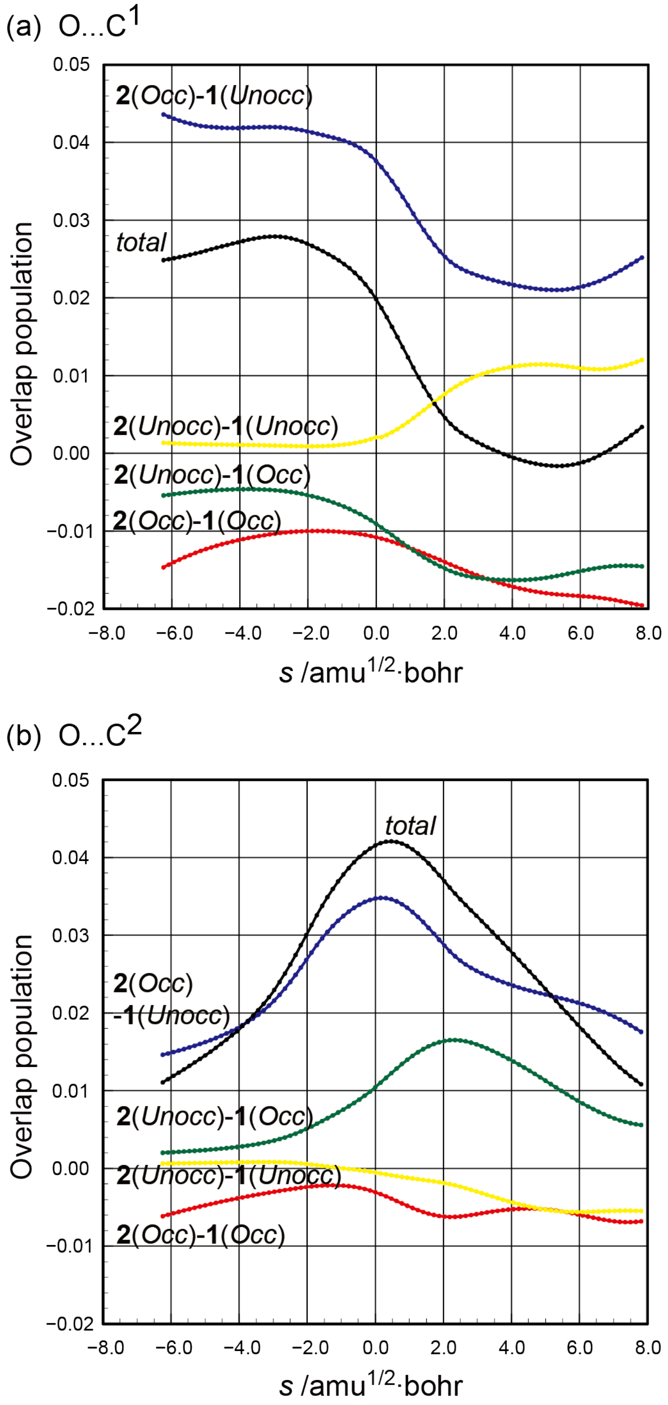

Figure S7. Change in the Mulliken overlap populations (a) between O and C^1 atoms and (b) between O and C^2 atoms along the IRC of **TS1**.

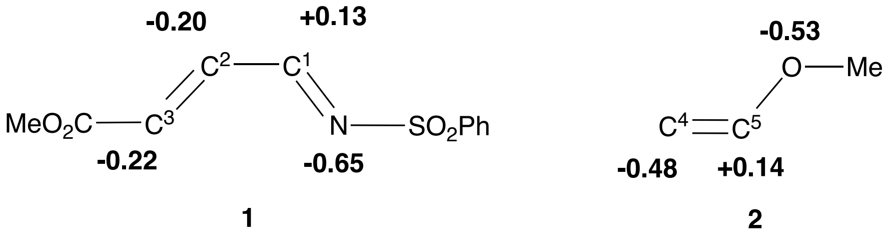

Figure S8. Natural population atomic charges in **1** and **2**.

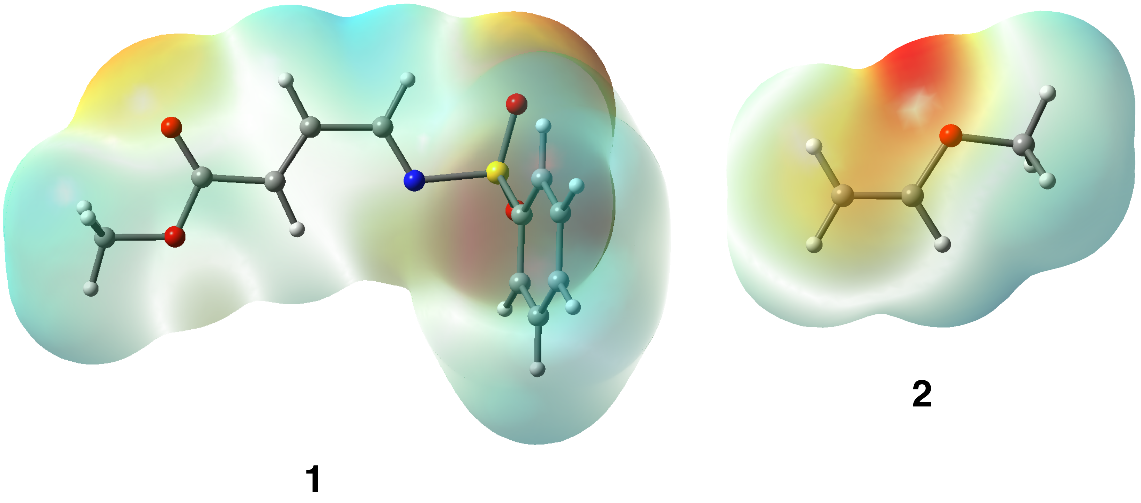

Figure S9. The electrostatic potential (ESP) maps on the isodensity (0.0004 e/au^3) surfaces of reactants, **1** and **2**, at the M06-2X/6-311G(d,p) level of theory. Negative value regions are described in red and positive regions are in blue.

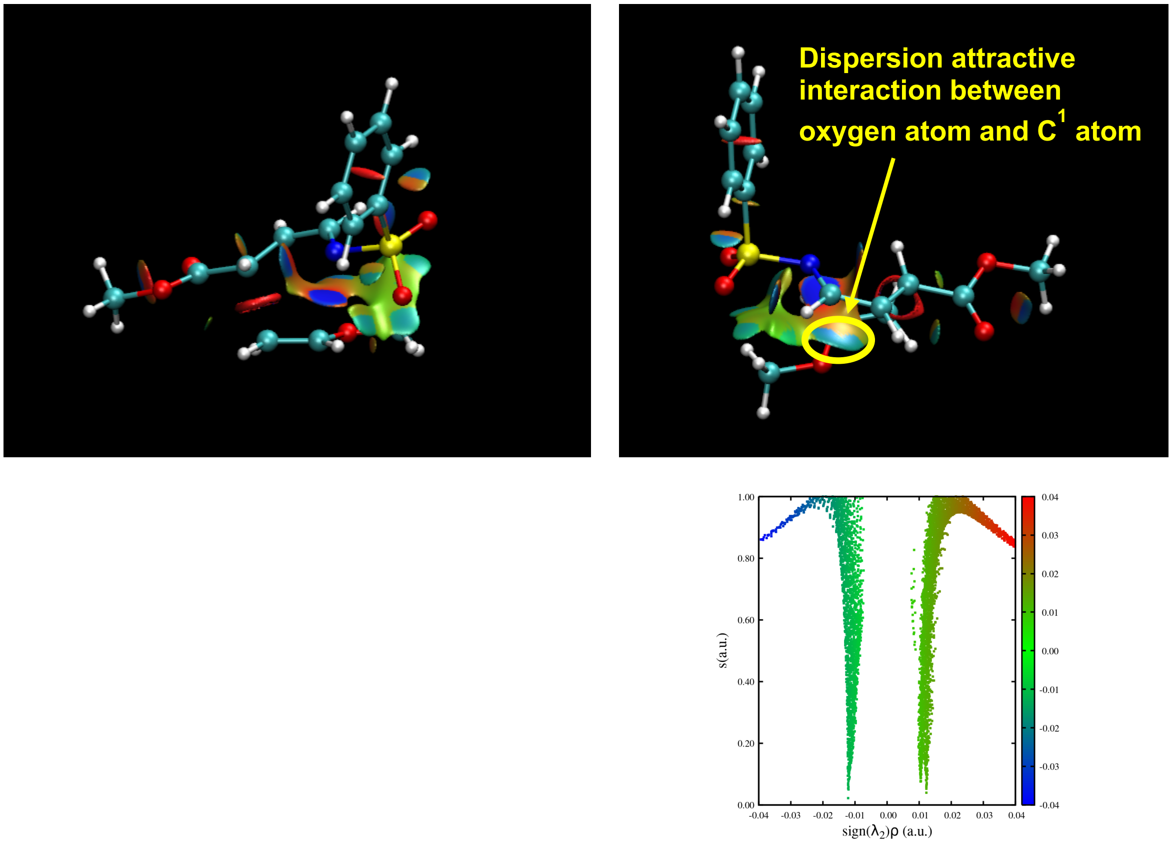

Figure S10. NCI plot for **TS1**.

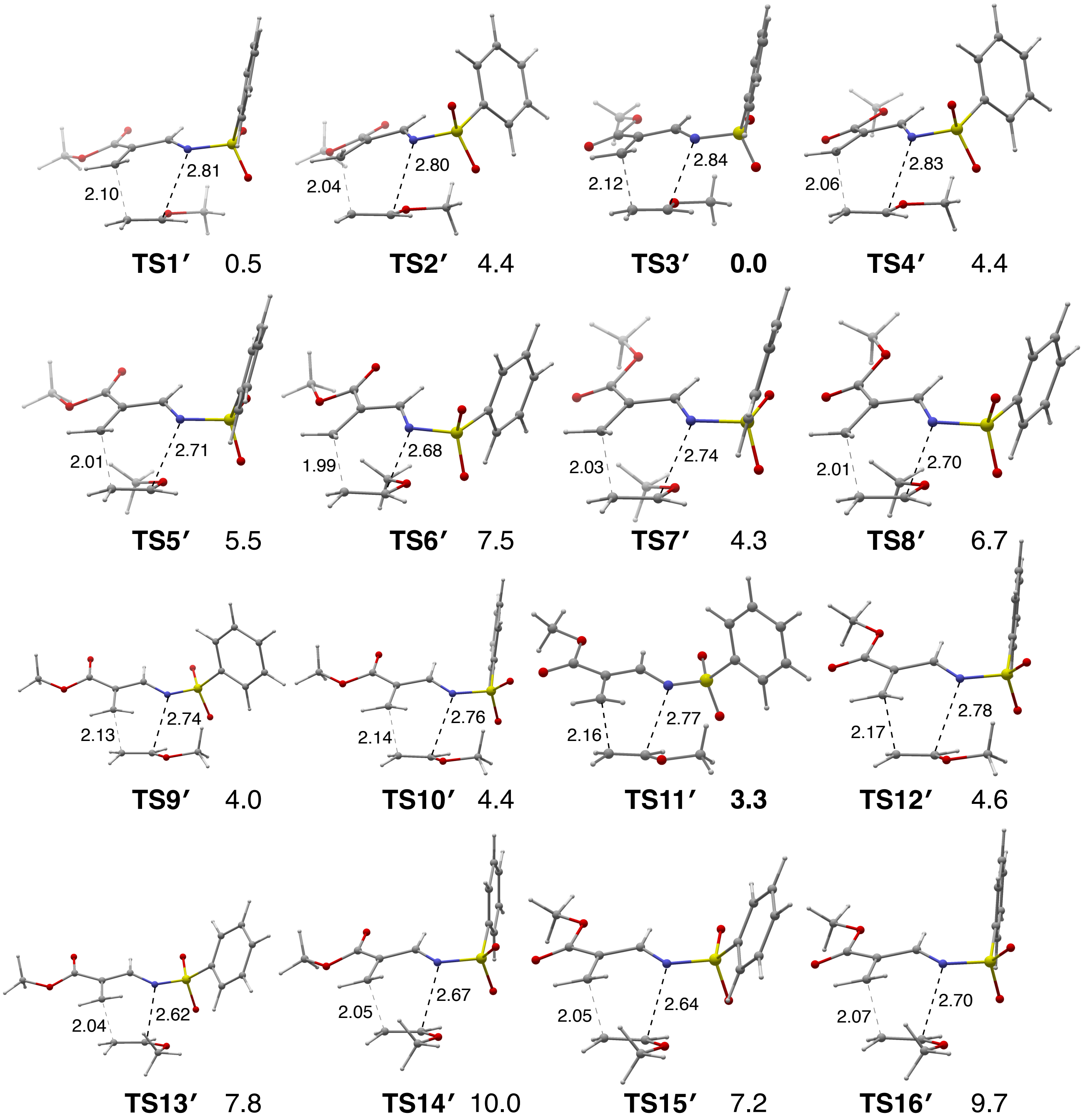

Figure S11. Transition state structures of **TS1′-TS32′**. Gibbs free energies relative to **TS3′** are shown in kcal/mol. Distances are shown in Å.

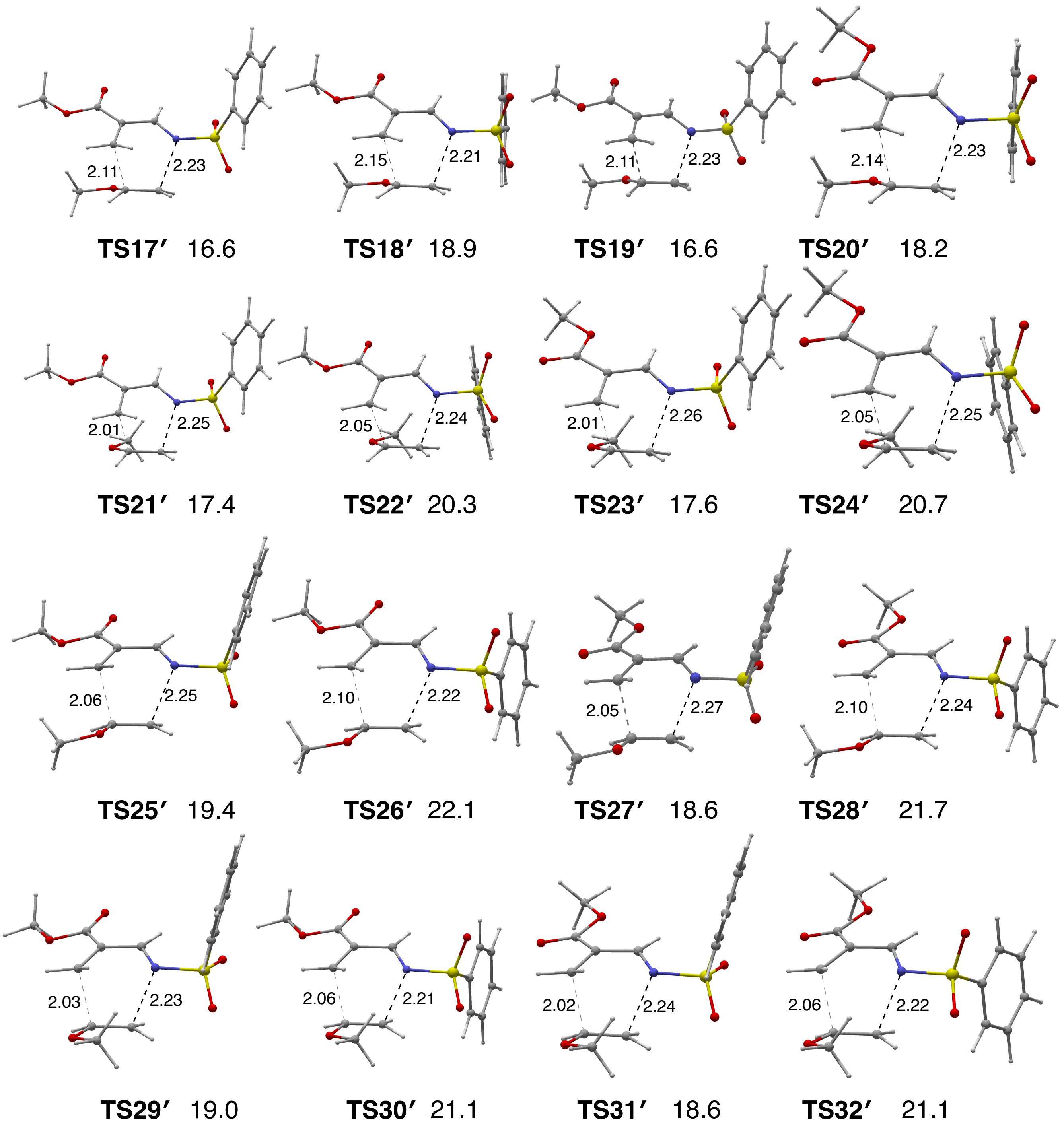

Figure S11. (*Continued.*)

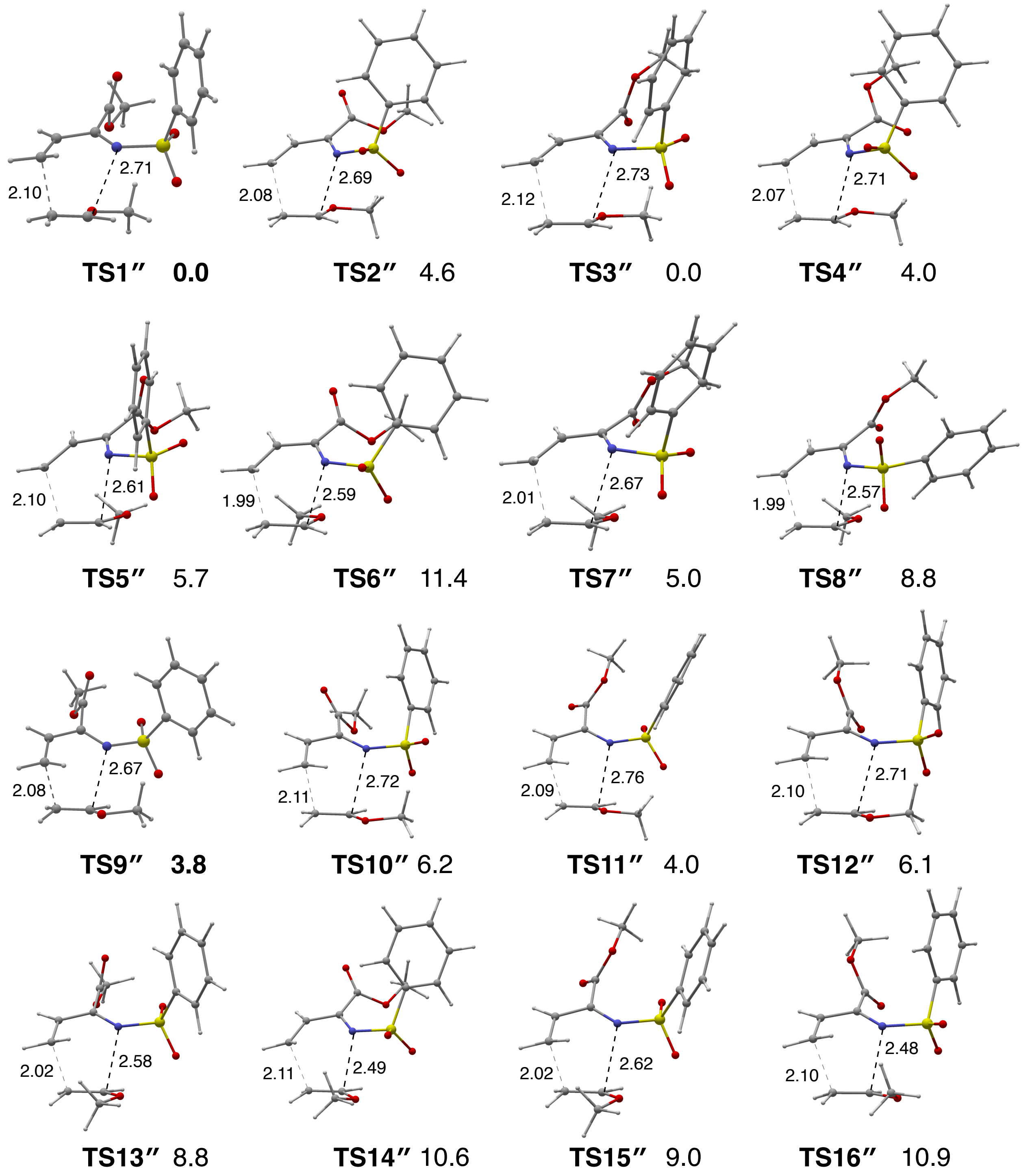

Figure S12. Transition state structures of **TS1″-TS32″**. Gibbs free energies relative to **TS1″** are shown in kcal/mol. Distances are shown in Å.

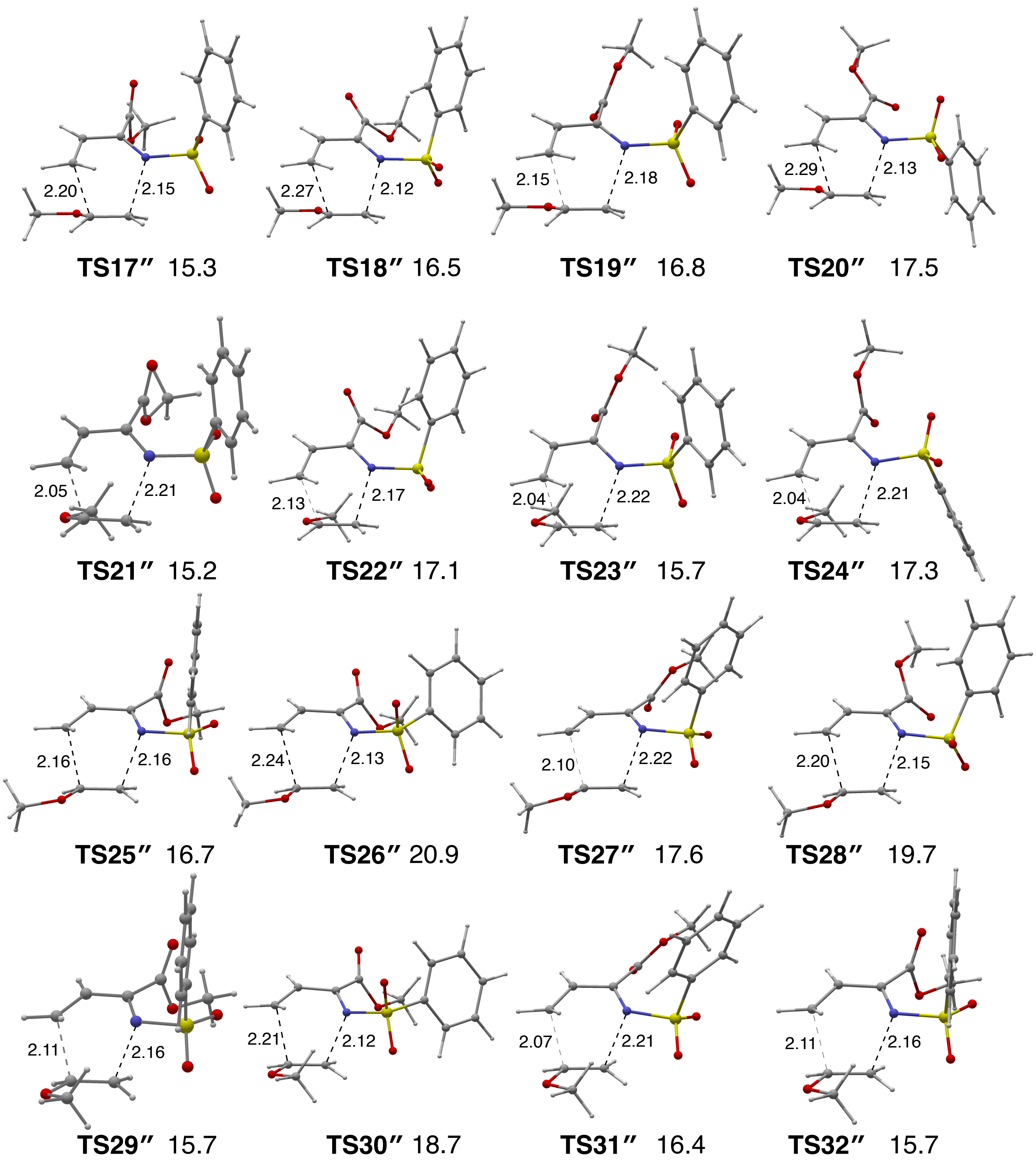

Figure S12. (*Continued.*)

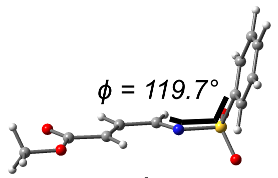

1

TS1 (*endo*): *ϕ = 109.7°*

TS9 (*exo*): *ϕ = 117.9°*

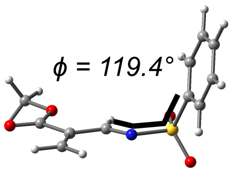

1′

TS3′ (*endo*): *ϕ = 112.6°*

TS11′ (*exo*): *ϕ = 120.4°*

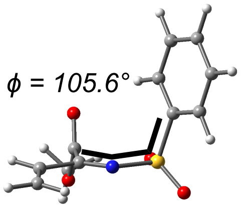

1″

TS3″ (*endo*): *ϕ = 105.2°*

TS9″ (*exo*): *ϕ = 108.3°*

Figure S13. Dihedral angle of C(Ph gorup)−S−N−C^{1}, ϕ (degree).

Cartesian coordinates of stationary points at the M06-2X/6-311G(d,p)[6D] level of theory are given below:

1 (NIMAG=0)

Center Number	Atomic Number	Atomic Type	Coordinates (Angstroms) X	Y	Z
1	6	0	-2.686256	-0.099023	0.023002
2	6	0	-2.059786	-0.977173	-0.757418
3	6	0	-0.650044	-1.351483	-0.567266
4	1	0	-2.605578	-1.448624	-1.568165
5	1	0	-0.239383	-2.136037	-1.209292
6	16	0	1.669603	-1.354584	0.451461
7	8	0	1.948749	-2.330785	-0.587998
8	8	0	1.847893	-1.681518	1.846985
9	6	0	2.557003	0.136708	0.078171
10	6	0	2.643697	1.118386	1.058637
11	6	0	3.134427	0.284416	-1.175840
12	6	0	3.330251	2.288661	0.764314
13	1	0	2.193005	0.952390	2.029589
14	6	0	3.821706	1.460416	-1.453958
15	1	0	3.055311	-0.516060	-1.901108
16	6	0	3.915055	2.457609	-0.488356
17	1	0	3.414338	3.066313	1.513181
18	1	0	4.287648	1.595099	-2.422166
19	1	0	4.452408	3.371712	-0.710838
20	6	0	-4.115477	0.224732	-0.237574
21	8	0	-4.780288	-0.245525	-1.120364
22	8	0	-4.569804	1.117667	0.648229
23	6	0	-5.940199	1.491148	0.480996
24	1	0	-6.147866	2.210542	1.267741
25	1	0	-6.090996	1.936600	-0.502542
26	1	0	-6.582449	0.615423	0.576257
27	7	0	0.066153	-0.778407	0.315609
28	1	0	-2.187820	0.389742	0.851646

1$_{\text{s-trans}}$ (NIMAG=0)

Center Number	Atomic Number	Atomic Type	Coordinates (Angstroms) X	Y	Z
1	6	0	3.117390	0.178578	0.338979
2	6	0	2.007922	0.111998	-0.395871
3	6	0	0.752260	0.688656	0.076260
4	1	0	2.015991	-0.371889	-1.366986
5	1	0	0.741041	1.167507	1.061318
6	16	0	-1.684039	1.374524	-0.003059
7	8	0	-2.136232	2.300363	-1.015189
8	8	0	-1.435051	1.818427	1.358557
9	6	0	-2.775774	-0.023664	0.049271
10	6	0	-3.050736	-0.622296	1.271305
11	6	0	-3.323833	-0.479619	-1.144379
12	6	0	-3.906164	-1.718108	1.294043
13	1	0	-2.612334	-0.220561	2.176506
14	6	0	-4.172388	-1.577508	-1.105564
15	1	0	-3.093320	0.028108	-2.073102
16	6	0	-4.460390	-2.193568	0.110037
17	1	0	-4.142309	-2.196813	2.236256
18	1	0	-4.613175	-1.949736	-2.021930
19	1	0	-5.126101	-3.048022	0.134037
20	6	0	4.384364	-0.416603	-0.167498

21	8	0	4.506967	-0.984303	-1.217569
22	8	0	5.377613	-0.234546	0.709903
23	6	0	6.642146	-0.771219	0.310117
24	1	0	7.329019	-0.541733	1.119502
25	1	0	6.562753	-1.848233	0.161396
26	1	0	6.969388	-0.307161	-0.620416
27	7	0	-0.289455	0.625396	-0.653480
28	1	0	3.144701	0.658984	1.310909

2 (NIMAG=0)

Center Number	Atomic Number	Atomic Type	Coordinates (Angstroms) X	Y	Z
1	6	0	-1.865006	-0.069760	0.034592
2	6	0	-0.609253	0.349553	-0.032795
3	1	0	-2.673284	0.646447	0.031970
4	1	0	-2.092814	-1.126371	0.085462
5	1	0	-0.352436	1.406623	-0.083893
6	8	0	0.443115	-0.505217	-0.059916
7	6	0	1.711864	0.111333	0.040420
8	1	0	2.452667	-0.681536	-0.036087
9	1	0	1.867166	0.827801	-0.773023
10	1	0	1.828158	0.622022	1.001590

TS1 (NIMAG=1; 413.2*i* cm^{-1})

Center Number	Atomic Number	Atomic Type	Coordinates (Angstroms) X	Y	Z
1	6	0	-2.213208	-0.605458	0.313791
2	6	0	-1.654262	0.043169	1.414657
3	6	0	-0.340250	0.501963	1.295100
4	1	0	-2.263837	0.298106	2.270350
5	1	0	0.132662	1.051729	2.110406
6	16	0	1.780571	1.032728	-0.018385
7	8	0	2.146965	1.832013	1.147612
8	8	0	1.796814	1.653659	-1.337301
9	6	0	2.855053	-0.385960	-0.062844
10	6	0	2.918320	-1.140861	-1.228279
11	6	0	3.588497	-0.713622	1.069132
12	6	0	3.740522	-2.259267	-1.253561
13	1	0	2.345608	-0.838427	-2.096430
14	6	0	4.409100	-1.835808	1.029352
15	1	0	3.523787	-0.085399	1.949017
16	6	0	4.481317	-2.606289	-0.126239
17	1	0	3.808107	-2.857765	-2.153721
18	1	0	4.995238	-2.104287	1.899634
19	1	0	5.123114	-3.478821	-0.152207
20	6	0	-3.601856	-1.144511	0.451349
21	8	0	-4.393151	-0.815453	1.291320
22	8	0	-3.870677	-2.034535	-0.515458
23	6	0	-5.189403	-2.585814	-0.477943
24	1	0	-5.244282	-3.277937	-1.313464
25	1	0	-5.354097	-3.105204	0.466154
26	1	0	-5.932288	-1.794264	-0.580633
27	7	0	0.278880	0.353912	0.137703
28	1	0	-1.550299	-1.175849	-0.324586
29	6	0	-2.591132	0.707388	-1.207039
30	6	0	-1.563377	1.630573	-1.154641

31	1	0	-2.623553	0.030984	-2.050067
32	1	0	-3.539680	1.040776	-0.800928
33	1	0	-0.688130	1.621140	-1.796640
34	8	0	-1.657623	2.578670	-0.251049
35	6	0	-0.574162	3.514422	-0.140508
36	1	0	-1.023920	4.492786	0.012390
37	1	0	0.042078	3.245129	0.717751
38	1	0	0.038605	3.501892	-1.041741

TS2 (NIMAG=1; 428.3*i* cm^{-1})

Center Number	Atomic Number	Atomic Type	Coordinates (Angstroms) X	Y	Z
1	6	0	2.452262	-0.511841	0.031304
2	6	0	1.412300	-0.917671	-0.810578
3	6	0	0.105530	-0.703291	-0.364352
4	1	0	1.618466	-1.277933	-1.808583
5	1	0	-0.736428	-1.016740	-0.985415
6	16	0	-1.546900	0.275275	1.419067
7	8	0	-1.669069	1.728847	1.473439
8	8	0	-1.662077	-0.496546	2.639122
9	6	0	-2.811482	-0.322148	0.303266
10	6	0	-3.073995	-1.688076	0.264517
11	6	0	-3.513356	0.577563	-0.488589
12	6	0	-4.046149	-2.160962	-0.607750
13	1	0	-2.532625	-2.357453	0.923226
14	6	0	-4.490672	0.093639	-1.352738
15	1	0	-3.311769	1.637813	-0.394122
16	6	0	-4.749277	-1.271523	-1.416691
17	1	0	-4.262697	-3.221320	-0.649254
18	1	0	-5.056207	0.783266	-1.967429
19	1	0	-5.511009	-1.644335	-2.090890
20	6	0	3.850899	-0.822019	-0.402531
21	8	0	4.186097	-1.087409	-1.524029
22	8	0	4.704279	-0.734210	0.627501
23	6	0	6.072577	-0.992042	0.302627
24	1	0	6.622599	-0.882527	1.232956
25	1	0	6.181706	-2.001176	-0.094763
26	1	0	6.424495	-0.278031	-0.442590
27	7	0	-0.070777	-0.037656	0.757976
28	1	0	2.273215	-0.564778	1.098731
29	6	0	2.627594	1.492317	0.107599
30	6	0	1.334693	1.968226	-0.027866
31	1	0	3.120071	1.631364	1.060304
32	1	0	3.242856	1.571303	-0.782116
33	1	0	0.706648	2.316100	0.785492
34	8	0	0.836278	1.969881	-1.242938
35	6	0	-0.525309	2.377145	-1.415874
36	1	0	-0.542895	3.085011	-2.242418
37	1	0	-1.113262	1.495044	-1.671620
38	1	0	-0.915066	2.823195	-0.501216

TS3 (NIMAG=1; 407.9*i* cm^{-1})

Center Number	Atomic Number	Atomic Type	Coordinates (Angstroms) X	Y	Z
1	6	0	2.144303	-0.825221	0.040769
2	6	0	1.710023	-0.270467	-1.164783
3	6	0	0.419379	0.266536	-1.195325

4	1	0	2.386541	-0.130957	-1.995338
5	1	0	0.041064	0.748869	-2.098181
6	16	0	-1.753247	1.050466	-0.114914
7	8	0	-1.985599	1.734150	-1.384410
8	8	0	-1.821939	1.814902	1.124391
9	6	0	-2.913826	-0.294146	-0.001101
10	6	0	-3.079436	-0.932000	1.222929
11	6	0	-3.611442	-0.682888	-1.136228
12	6	0	-3.969742	-1.993873	1.305268
13	1	0	-2.529921	-0.585045	2.089414
14	6	0	-4.501856	-1.746980	-1.038712
15	1	0	-3.464564	-0.144972	-2.064695
16	6	0	-4.676686	-2.400826	0.176122
17	1	0	-4.116202	-2.501723	2.250581
18	1	0	-5.061495	-2.061258	-1.911102
19	1	0	-5.371803	-3.228910	0.246625
20	6	0	3.470517	-1.508263	0.134024
21	8	0	3.721720	-2.382681	0.917020
22	8	0	4.362451	-1.011395	-0.735464
23	6	0	5.653271	-1.624542	-0.691396
24	1	0	6.248441	-1.113684	-1.443030
25	1	0	6.093847	-1.505251	0.298757
26	1	0	5.573537	-2.687854	-0.917740
27	7	0	-0.289383	0.277779	-0.082144
28	1	0	1.393822	-1.281193	0.674153
29	6	0	2.508221	0.622652	1.431282
30	6	0	1.543958	1.587369	1.210480
31	1	0	2.446942	0.040322	2.340648
32	1	0	3.498021	0.855142	1.054751
33	1	0	0.628173	1.691239	1.784040
34	8	0	1.752539	2.426116	0.221351
35	6	0	0.736195	3.399984	-0.062380
36	1	0	1.251843	4.333519	-0.275992
37	1	0	0.167204	3.076100	-0.934319
38	1	0	0.061528	3.510976	0.786266

TS4 (NIMAG=1; 422.6*i* cm^{-1})

Center Number	Atomic Number	Atomic Type	Coordinates (Angstroms) X	Y	Z
1	6	0	2.464799	-0.288342	0.564171
2	6	0	1.514029	-1.027769	-0.149836
3	6	0	0.168292	-0.715426	0.068748
4	1	0	1.804799	-1.725404	-0.921394
5	1	0	-0.607548	-1.276297	-0.457290
6	16	0	-1.655673	0.792861	1.192623
7	8	0	-1.790659	2.151270	0.676055
8	8	0	-1.885545	0.537292	2.599179
9	6	0	-2.800977	-0.237043	0.282054
10	6	0	-3.052071	-1.524607	0.746062
11	6	0	-3.425528	0.263152	-0.853228
12	6	0	-3.931392	-2.334285	0.038350
13	1	0	-2.575506	-1.869251	1.656661
14	6	0	-4.310685	-0.553842	-1.549748
15	1	0	-3.239529	1.287158	-1.154073
16	6	0	-4.555479	-1.850117	-1.108822
17	1	0	-4.138369	-3.338485	0.387249
18	1	0	-4.816343	-0.174320	-2.429260
19	1	0	-5.245698	-2.482960	-1.653707
20	6	0	3.912949	-0.654846	0.490018
21	8	0	4.702273	-0.459667	1.372249

22	8	0	4.241105	-1.198107	-0.692417
23	6	0	5.616013	-1.567598	-0.823765
24	1	0	5.716143	-1.982457	-1.822831
25	1	0	6.255163	-0.692779	-0.701573
26	1	0	5.881456	-2.307802	-0.068879
27	7	0	-0.120207	0.315427	0.833567
28	1	0	2.172654	0.055065	1.549539
29	6	0	2.632654	1.590225	-0.124203
30	6	0	1.357146	1.932101	-0.537573
31	1	0	3.038913	2.099925	0.739076
32	1	0	3.323870	1.339386	-0.921350
33	1	0	0.656354	2.537015	0.028212
34	8	0	0.969283	1.452573	-1.697334
35	6	0	-0.371947	1.714395	-2.124666
36	1	0	-0.315489	2.071858	-3.151225
37	1	0	-0.927210	0.776513	-2.089328
38	1	0	-0.849920	2.446183	-1.473984

TS5 (NIMAG=1; 458.8i cm^{-1})

Center Number	Atomic Number	Atomic Type	Coordinates (Angstroms) X	Y	Z
1	6	0	-2.054078	0.672518	-0.249640
2	6	0	-1.561942	-0.050629	-1.354417
3	6	0	-0.269950	-0.560833	-1.265877
4	1	0	-2.216406	-0.316344	-2.173380
5	1	0	0.146725	-1.179743	-2.060524
6	16	0	1.836481	-1.176674	0.028195
7	8	0	2.136652	-2.011645	-1.126729
8	8	0	1.817346	-1.760363	1.362067
9	6	0	3.007252	0.167774	0.040981
10	6	0	3.162546	0.911806	1.204497
11	6	0	3.723324	0.451129	-1.113960
12	6	0	4.059246	1.972082	1.204634
13	1	0	2.602046	0.642903	2.091422
14	6	0	4.619079	1.514890	-1.100394
15	1	0	3.587404	-0.167753	-1.992437
16	6	0	4.782982	2.273780	0.053630
17	1	0	4.198846	2.559667	2.103809
18	1	0	5.192588	1.746513	-1.989559
19	1	0	5.483488	3.100368	0.059658
20	6	0	-3.451793	1.189916	-0.373773
21	8	0	-4.336484	0.625402	-0.963393
22	8	0	-3.617896	2.335481	0.293480
23	6	0	-4.940109	2.881298	0.245425
24	1	0	-4.902551	3.795409	0.830796
25	1	0	-5.221964	3.093017	-0.785863
26	1	0	-5.654236	2.176221	0.671438
27	7	0	0.388769	-0.381874	-0.133686
28	1	0	-1.353428	1.335170	0.244842
29	6	0	-2.262416	-0.405314	1.389914
30	6	0	-1.393342	-1.488598	1.247801
31	1	0	-2.005439	0.295639	2.173814
32	1	0	-3.323099	-0.610058	1.292210
33	1	0	-0.449847	-1.592074	1.768009
34	8	0	-1.640753	-2.485840	0.436282
35	6	0	-2.960007	-2.664175	-0.103572
36	1	0	-2.857962	-3.442505	-0.854018
37	1	0	-3.623111	-3.001820	0.697160
38	1	0	-3.343817	-1.752839	-0.557843

TS6 (NIMAG=1; 481.2*i* cm^{-1})

Center Number	Atomic Number	Atomic Type	Coordinates (Angstroms) X	Y	Z
1	6	0	2.351667	-0.402962	-0.452193
2	6	0	1.326542	0.167031	-1.227025
3	6	0	0.020300	-0.242310	-0.967564
4	1	0	1.533968	0.995030	-1.891450
5	1	0	-0.808334	0.208637	-1.515258
6	16	0	-1.701821	-1.524280	0.493055
7	8	0	-1.699540	-1.454892	1.946920
8	8	0	-2.049278	-2.762417	-0.174947
9	6	0	-2.827146	-0.265508	-0.097011
10	6	0	-3.613235	-0.528752	-1.210774
11	6	0	-2.872263	0.958754	0.561985
12	6	0	-4.471494	0.463548	-1.675173
13	1	0	-3.554820	-1.501824	-1.683529
14	6	0	-3.730579	1.941671	0.086687
15	1	0	-2.247950	1.127838	1.431205
16	6	0	-4.526988	1.693815	-1.029396
17	1	0	-5.098718	0.272685	-2.537323
18	1	0	-3.785817	2.898795	0.590981
19	1	0	-5.197894	2.463105	-1.392929
20	6	0	3.735513	0.094227	-0.721688
21	8	0	3.998736	1.219622	-1.059178
22	8	0	4.654000	-0.847352	-0.493192
23	6	0	6.010085	-0.439676	-0.701777
24	1	0	6.617362	-1.310398	-0.472480
25	1	0	6.153724	-0.128634	-1.736383
26	1	0	6.258616	0.392160	-0.042334
27	7	0	-0.173200	-1.067952	0.041566
28	1	0	2.263022	-1.459811	-0.229172
29	6	0	2.231450	0.039617	1.482290
30	6	0	0.894972	0.347419	1.729716
31	1	0	2.591354	-0.869523	1.946487
32	1	0	2.938258	0.861797	1.473292
33	1	0	0.207189	-0.299357	2.258443
34	8	0	0.354926	1.493581	1.365735
35	6	0	1.196440	2.580067	0.951065
36	1	0	0.520194	3.330293	0.550707
37	1	0	1.719279	2.977186	1.825181
38	1	0	1.909769	2.276194	0.187176

TS7 (NIMAG=1; 457.9*i* cm^{-1})

Center Number	Atomic Number	Atomic Type	Coordinates (Angstroms) X	Y	Z
1	6	0	1.974531	-0.910215	0.027310
2	6	0	1.582072	-0.293334	-1.178227
3	6	0	0.320349	0.296664	-1.209799
4	1	0	2.279120	-0.159497	-1.993367
5	1	0	-0.021235	0.846019	-2.087263
6	16	0	-1.797939	1.174006	-0.099943
7	8	0	-1.986617	1.899074	-1.349038
8	8	0	-1.802505	1.893180	1.165962
9	6	0	-3.053488	-0.088258	-0.013041
10	6	0	-3.297487	-0.709077	1.206297
11	6	0	-3.745616	-0.433582	-1.165621
12	6	0	-4.260689	-1.707340	1.266452

13	1	0	-2.751373	-0.394691	2.087212
14	6	0	-4.709065	-1.433866	-1.091519
15	1	0	-3.538260	0.090323	-2.090651
16	6	0	-4.962159	-2.069888	0.119169
17	1	0	-4.468743	-2.199218	2.208766
18	1	0	-5.264966	-1.712032	-1.978520
19	1	0	-5.714455	-2.847861	0.171863
20	6	0	3.293801	-1.612105	0.080367
21	8	0	3.504902	-2.631775	0.673383
22	8	0	4.245476	-0.936609	-0.593481
23	6	0	5.536505	-1.553614	-0.593427
24	1	0	6.177959	-0.899218	-1.177114
25	1	0	5.907681	-1.651802	0.426962
26	1	0	5.479807	-2.543054	-1.046667
27	7	0	-0.398980	0.280957	-0.101655
28	1	0	1.197694	-1.447013	0.559382
29	6	0	2.209419	0.347615	1.524141
30	6	0	1.374993	1.433025	1.254761
31	1	0	1.925918	-0.258029	2.375410
32	1	0	3.276745	0.504512	1.412974
33	1	0	0.422015	1.609748	1.736179
34	8	0	1.670643	2.348132	0.363573
35	6	0	3.006387	2.444201	-0.149637
36	1	0	2.950156	3.166524	-0.958816
37	1	0	3.662344	2.819078	0.640713
38	1	0	3.366979	1.488036	-0.522929

TS8 (NIMAG=1; 481.1i cm^{-1})

Center Number	Atomic Number	Atomic Type	Coordinates (Angstroms) X	Y	Z
1	6	0	2.351710	-0.689581	-0.468786
2	6	0	1.408746	0.091482	-1.161114
3	6	0	0.062921	-0.224501	-0.977824
4	1	0	1.701427	0.984897	-1.694720
5	1	0	-0.705726	0.383672	-1.456953
6	16	0	-1.808363	-1.541632	0.249031
7	8	0	-1.834530	-1.696629	1.695824
8	8	0	-2.255053	-2.621745	-0.607180
9	6	0	-2.794962	-0.103577	-0.148757
10	6	0	-3.555928	-0.105565	-1.310238
11	6	0	-2.760593	0.993560	0.705709
12	6	0	-4.304767	1.025002	-1.623060
13	1	0	-3.564762	-0.988919	-1.937509
14	6	0	-3.510670	2.116528	0.380896
15	1	0	-2.160970	0.956304	1.607204
16	6	0	-4.279222	2.131535	-0.781123
17	1	0	-4.911253	1.037725	-2.520374
18	1	0	-3.504690	2.977376	1.038498
19	1	0	-4.866016	3.008701	-1.026784
20	6	0	3.806610	-0.406229	-0.661058
21	8	0	4.668917	-1.237071	-0.694741
22	8	0	4.048060	0.916951	-0.752845
23	6	0	5.422650	1.265119	-0.945555
24	1	0	5.447949	2.349449	-1.008881
25	1	0	6.022570	0.911105	-0.106891
26	1	0	5.798585	0.815890	-1.864511
27	7	0	-0.232689	-1.172898	-0.112590
28	1	0	2.134123	-1.749044	-0.397854
29	6	0	2.239321	-0.498619	1.502598
30	6	0	0.920714	-0.158182	1.795807

31	1	0	2.549517	-1.483005	1.828615
32	1	0	2.990903	0.276053	1.604376
33	1	0	0.191145	-0.841438	2.209922
34	8	0	0.449036	1.064017	1.633717
35	6	0	1.353198	2.151923	1.403879
36	1	0	0.723358	3.001319	1.153487
37	1	0	1.907451	2.356858	2.323939
38	1	0	2.039213	1.941894	0.585623

TS9 (NIMAG=1; 418.9*i* cm^{-1})

Center Number	Atomic Number	Atomic Type	Coordinates (Angstroms) X	Y	Z
1	6	0	2.407562	-0.489594	0.233709
2	6	0	1.815654	-1.674552	-0.195474
3	6	0	0.477043	-1.663785	-0.587793
4	1	0	2.436157	-2.537042	-0.398667
5	1	0	0.033470	-2.560388	-1.022160
6	16	0	-1.740197	-0.605877	-1.216390
7	8	0	-2.064013	-1.938434	-1.700648
8	8	0	-1.839500	0.530626	-2.124711
9	6	0	-2.789313	-0.259883	0.185316
10	6	0	-3.523881	0.916840	0.212081
11	6	0	-2.868253	-1.198198	1.208683
12	6	0	-4.353767	1.163987	1.302258
13	1	0	-3.449371	1.609215	-0.617918
14	6	0	-3.695259	-0.938843	2.291944
15	1	0	-2.294541	-2.115967	1.150254
16	6	0	-4.434926	0.241463	2.338494
17	1	0	-4.938714	2.074890	1.337451
18	1	0	-3.767817	-1.657823	3.098586
19	1	0	-5.081348	0.437827	3.185441
20	6	0	3.862337	-0.556162	0.590074
21	8	0	4.623442	-1.407978	0.223629
22	8	0	4.221299	0.479390	1.362692
23	6	0	5.601953	0.504863	1.734401
24	1	0	5.725755	1.392154	2.348731
25	1	0	5.857263	-0.393777	2.296040
26	1	0	6.231236	0.557356	0.845438
27	7	0	-0.220512	-0.542085	-0.538958
28	1	0	1.824058	0.230057	0.799557
29	6	0	2.529386	0.840755	-1.330741
30	6	0	1.263093	1.390229	-1.363765
31	1	0	3.287690	1.429392	-0.826388
32	1	0	2.843750	0.223655	-2.160676
33	1	0	0.515204	1.168499	-2.118092
34	8	0	0.950031	2.267900	-0.433239
35	6	0	-0.414517	2.689020	-0.346489
36	1	0	-0.886426	2.670118	-1.328803
37	1	0	-0.944405	2.014507	0.326917
38	1	0	-0.397981	3.697092	0.061273

TS10 (NIMAG=1; 416.4*i* cm^{-1})

Center Number	Atomic Number	Atomic Type	Coordinates (Angstroms) X	Y	Z
1	6	0	-2.298645	-0.722254	-0.177150
2	6	0	-1.177432	-1.524043	0.026684
3	6	0	0.091665	-0.946888	-0.049531

4	1	0	-1.299847	-2.529540	0.405821
5	1	0	0.961707	-1.565958	0.175829
6	16	0	1.689063	1.090476	-0.229270
7	8	0	1.690326	1.874569	1.002872
8	8	0	1.892286	1.771731	-1.495377
9	6	0	2.962051	-0.151502	-0.077528
10	6	0	3.451419	-0.750410	-1.232723
11	6	0	3.420210	-0.502919	1.185990
12	6	0	4.425525	-1.733788	-1.112543
13	1	0	3.083307	-0.429770	-2.200056
14	6	0	4.396871	-1.487138	1.291978
15	1	0	3.026749	0.006819	2.057193
16	6	0	4.893295	-2.101638	0.146420
17	1	0	4.824693	-2.207834	-2.000723
18	1	0	4.773444	-1.769474	2.267413
19	1	0	5.655016	-2.867123	0.234132
20	6	0	-3.632307	-1.396190	-0.057899
21	8	0	-3.837071	-2.417380	0.538190
22	8	0	-4.588072	-0.699012	-0.688206
23	6	0	-5.899523	-1.263200	-0.608369
24	1	0	-6.545200	-0.588217	-1.162880
25	1	0	-5.908561	-2.259031	-1.051550
26	1	0	-6.216391	-1.335944	0.432483
27	7	0	0.215944	0.338125	-0.306424
28	1	0	-2.249382	0.088194	-0.898139
29	6	0	-2.516680	0.623103	1.349052
30	6	0	-1.600289	1.611577	1.041608
31	1	0	-3.547995	0.838404	1.091606
32	1	0	-2.337543	0.014962	2.224587
33	1	0	-0.644610	1.731028	1.542762
34	8	0	-1.908457	2.477522	0.104105
35	6	0	-0.880687	3.378659	-0.334941
36	1	0	-0.347537	2.925601	-1.170541
37	1	0	-1.386985	4.287682	-0.650283
38	1	0	-0.182418	3.586036	0.476665

TS11 (NIMAG=1; 436.9i cm^{-1})

Center Number	Atomic Number	Atomic Type	Coordinates (Angstroms) X	Y	Z
1	6	0	2.327433	-0.411005	0.335817
2	6	0	1.833235	-1.417299	-0.495949
3	6	0	0.523799	-1.308690	-0.965791
4	1	0	2.498699	-2.159329	-0.913919
5	1	0	0.132165	-2.039988	-1.673827
6	16	0	-1.701072	-0.145857	-1.330035
7	8	0	-1.972812	-1.214555	-2.279886
8	8	0	-1.826692	1.237638	-1.777338
9	6	0	-2.779320	-0.379952	0.071929
10	6	0	-2.486693	0.248439	1.278180
11	6	0	-3.917100	-1.156451	-0.094976
12	6	0	-3.364754	0.093100	2.341854
13	1	0	-1.569370	0.816399	1.381617
14	6	0	-4.791357	-1.299184	0.978086
15	1	0	-4.097751	-1.642110	-1.046118
16	6	0	-4.517015	-0.675276	2.189594
17	1	0	-3.148380	0.565654	3.292140
18	1	0	-5.684088	-1.902207	0.866574
19	1	0	-5.198658	-0.793440	3.023377
20	6	0	3.722873	-0.518082	0.868239
21	8	0	4.072737	-0.092061	1.934292

22	8	0	4.549352	-1.118948	0.000939
23	6	0	5.899974	-1.254900	0.450306
24	1	0	6.431229	-1.750470	-0.357323
25	1	0	6.330162	-0.273737	0.652551
26	1	0	5.935862	-1.852242	1.361404
27	7	0	-0.201580	-0.258843	-0.629509
28	1	0	1.651175	0.064928	1.039221
29	6	0	2.528451	1.311903	-0.723125
30	6	0	1.267631	1.878632	-0.733505
31	1	0	3.225835	1.744254	-0.013541
32	1	0	2.931442	0.950099	-1.658172
33	1	0	0.605430	1.889704	-1.594424
34	8	0	0.840318	2.465610	0.365300
35	6	0	-0.370801	3.228988	0.267026
36	1	0	-1.097207	2.711423	-0.360419
37	1	0	-0.741390	3.342168	1.282883
38	1	0	-0.138309	4.211687	-0.147480

TS12 (NIMAG=1; 406.5*i* cm^{-1})

Center Number	Atomic Number	Atomic Type	Coordinates (Angstroms) X	Y	Z
1	6	0	-2.369574	-0.423513	-0.425211
2	6	0	-1.324947	-1.320601	-0.202533
3	6	0	-0.014110	-0.836192	-0.177544
4	1	0	-1.529577	-2.332491	0.116925
5	1	0	0.793856	-1.531425	0.057728
6	16	0	1.733756	1.079885	-0.141044
7	8	0	1.720212	1.769343	1.146232
8	8	0	2.055813	1.833696	-1.339202
9	6	0	2.905059	-0.261773	-0.020109
10	6	0	3.406165	-0.817577	-1.191772
11	6	0	3.275671	-0.728687	1.234630
12	6	0	4.301209	-1.876031	-1.097936
13	1	0	3.109204	-0.407320	-2.149693
14	6	0	4.174194	-1.787135	1.314404
15	1	0	2.877688	-0.249485	2.120995
16	6	0	4.680637	-2.359630	0.151644
17	1	0	4.708488	-2.318362	-1.998706
18	1	0	4.482874	-2.159859	2.283330
19	1	0	5.381631	-3.183024	0.218973
20	6	0	-3.774692	-0.936953	-0.483134
21	8	0	-4.640887	-0.460341	-1.163058
22	8	0	-3.966212	-1.979242	0.339096
23	6	0	-5.290215	-2.518468	0.327930
24	1	0	-5.280531	-3.340136	1.038589
25	1	0	-6.010106	-1.756211	0.626921
26	1	0	-5.544882	-2.873373	-0.670809
27	7	0	0.218492	0.446365	-0.351544
28	1	0	-2.204531	0.415477	-1.094370
29	6	0	-2.598171	0.843487	1.167924
30	6	0	-1.593467	1.774003	0.981830
31	1	0	-3.591641	1.152863	0.860824
32	1	0	-2.522862	0.171101	2.010785
33	1	0	-0.662364	1.781193	1.540593
34	8	0	-1.774273	2.719360	0.090203
35	6	0	-0.654868	3.559822	-0.228757
36	1	0	-0.113851	3.122462	-1.067567
37	1	0	-1.068475	4.527556	-0.501543
38	1	0	0.010967	3.651285	0.630164

TS13 (NIMAG=1; 489.3i cm^{-1})

Center Number	Atomic Number	Atomic Type	Coordinates (Angstroms) X	Y	Z
1	6	0	-2.226968	-0.446576	-0.561871
2	6	0	-1.676285	-1.671531	-0.951284
3	6	0	-0.333991	-1.886190	-0.643587
4	1	0	-2.313155	-2.473584	-1.298528
5	1	0	0.148979	-2.838033	-0.864148
6	16	0	1.864759	-1.299467	0.511933
7	8	0	2.351355	-2.541788	-0.069323
8	8	0	1.881365	-1.127509	1.957428
9	6	0	2.780414	0.047019	-0.208169
10	6	0	2.583164	1.327067	0.298803
11	6	0	3.666339	-0.210667	-1.243107
12	6	0	3.298796	2.377959	-0.257129
13	1	0	1.887134	1.477339	1.116666
14	6	0	4.378083	0.853213	-1.790154
15	1	0	3.794648	-1.226765	-1.595538
16	6	0	4.192649	2.140612	-1.300202
17	1	0	3.166477	3.383046	0.124799
18	1	0	5.079522	0.673371	-2.595581
19	1	0	4.750374	2.965027	-1.728393
20	6	0	-3.668018	-0.194621	-0.873180
21	8	0	-4.484914	-1.034276	-1.124755
22	8	0	-3.950403	1.120441	-0.805407
23	6	0	-5.314395	1.458555	-1.075332
24	1	0	-5.374990	2.539739	-0.987666
25	1	0	-5.589236	1.136879	-2.079698
26	1	0	-5.973393	0.973095	-0.355003
27	7	0	0.314242	-0.942769	0.017710
28	1	0	-1.583435	0.421292	-0.644850
29	6	0	-2.303124	-0.359793	1.437317
30	6	0	-1.016902	-0.061677	1.883530
31	1	0	-3.049411	0.424866	1.493109
32	1	0	-2.661187	-1.354924	1.663255
33	1	0	-0.348720	-0.786018	2.328056
34	8	0	-0.504552	1.155022	1.923642
35	6	0	-1.321705	2.265202	1.556244
36	1	0	-2.103185	2.412781	2.306503
37	1	0	-0.659130	3.126317	1.542455
38	1	0	-1.778300	2.129870	0.575716

TS14 (NIMAG=1; 486.2i cm^{-1})

Center Number	Atomic Number	Atomic Type	Coordinates (Angstroms) X	Y	Z
1	6	0	2.213846	-0.832004	0.057108
2	6	0	1.171171	-1.529229	-0.549193
3	6	0	-0.097216	-0.946136	-0.508133
4	1	0	1.380255	-2.377655	-1.186758
5	1	0	-0.938014	-1.431832	-1.007302
6	16	0	-1.640341	1.088038	-0.057562
7	8	0	-1.734570	1.655915	-1.395382
8	8	0	-1.681723	1.944622	1.110391
9	6	0	-2.946830	-0.120218	0.102437
10	6	0	-3.235289	-0.627067	1.364834
11	6	0	-3.635982	-0.531691	-1.030706
12	6	0	-4.237991	-1.579492	1.488378

13	1	0	-2.690860	-0.262162	2.227659
14	6	0	-4.640395	-1.484877	-0.892781
15	1	0	-3.393059	-0.090772	-1.990144
16	6	0	-4.935966	-2.008051	0.361394
17	1	0	-4.481237	-1.982501	2.463811
18	1	0	-5.194249	-1.813328	-1.763690
19	1	0	-5.719636	-2.749129	0.464277
20	6	0	3.592929	-1.398203	-0.063387
21	8	0	3.938428	-2.203909	-0.882785
22	8	0	4.418192	-0.867057	0.851487
23	6	0	5.767317	-1.338770	0.793291
24	1	0	6.296119	-0.820728	1.588316
25	1	0	5.795684	-2.417242	0.948708
26	1	0	6.205464	-1.109086	-0.178329
27	7	0	-0.207397	0.239817	0.053000
28	1	0	2.007420	-0.297560	0.974481
29	6	0	2.561265	0.951379	-0.922266
30	6	0	1.411238	1.731287	-0.995724
31	1	0	3.361819	1.269201	-0.264575
32	1	0	2.866269	0.487761	-1.850827
33	1	0	0.750669	1.691484	-1.854363
34	8	0	1.073115	2.721483	-0.193813
35	6	0	1.670032	2.778806	1.095221
36	1	0	2.741791	2.982964	1.022336
37	1	0	1.168547	3.590036	1.614610
38	1	0	1.483931	1.840399	1.619762

TS15 (NIMAG=1; 475.6*i* cm^{-1})

Center Number	Atomic Number	Atomic Type	Coordinates (Angstroms) X	Y	Z
1	6	0	-2.211087	-0.207052	-0.456701
2	6	0	-1.784183	-1.534627	-0.596500
3	6	0	-0.463047	-1.803312	-0.240497
4	1	0	-2.487091	-2.334109	-0.781120
5	1	0	-0.073262	-2.821253	-0.254600
6	16	0	1.790167	-1.196244	0.791682
7	8	0	2.147023	-2.580284	0.516020
8	8	0	1.835331	-0.704059	2.161690
9	6	0	2.829702	-0.146004	-0.200998
10	6	0	2.718841	1.231979	-0.046828
11	6	0	3.726035	-0.721534	-1.088410
12	6	0	3.534089	2.052573	-0.813006
13	1	0	2.007457	1.637325	0.664281
14	6	0	4.539077	0.114291	-1.848845
15	1	0	3.781683	-1.800394	-1.166097
16	6	0	4.441196	1.493880	-1.712187
17	1	0	3.467735	3.128702	-0.708247
18	1	0	5.249724	-0.314507	-2.544696
19	1	0	5.076560	2.140118	-2.306101
20	6	0	-3.589819	0.212489	-0.855442
21	8	0	-3.888403	1.350754	-1.110278
22	8	0	-4.461015	-0.799189	-0.856540
23	6	0	-5.799488	-0.449348	-1.221638
24	1	0	-6.368591	-1.373178	-1.173196
25	1	0	-6.198370	0.289102	-0.525925
26	1	0	-5.817972	-0.035064	-2.229551
27	7	0	0.280170	-0.808647	0.211951
28	1	0	-1.474672	0.551510	-0.697424
29	6	0	-2.275738	0.265145	1.475932
30	6	0	-0.971932	0.615458	1.824749

31	1	0	-3.001263	1.066050	1.385161
32	1	0	-2.649680	-0.649830	1.915177
33	1	0	-0.307597	-0.012708	2.402561
34	8	0	-0.424362	1.782907	1.552519
35	6	0	-1.230729	2.818331	0.982653
36	1	0	-1.953525	3.166457	1.724909
37	1	0	-0.542432	3.621324	0.734823
38	1	0	-1.758676	2.486246	0.088500

TS16 (NIMAG=1; 458.8i cm^{-1})

Center Number	Atomic Number	Atomic Type	Coordinates (Angstroms) X	Y	Z
1	6	0	-2.188054	-0.545330	-0.322229
2	6	0	-1.120434	-1.432752	-0.121694
3	6	0	0.161520	-0.881233	-0.137906
4	1	0	-1.287413	-2.446317	0.212755
5	1	0	1.022594	-1.517680	0.072571
6	16	0	1.749231	1.188511	-0.219704
7	8	0	1.724774	1.944075	1.026887
8	8	0	1.978348	1.875183	-1.474184
9	6	0	3.021133	-0.057297	-0.056164
10	6	0	3.539671	-0.639704	-1.207018
11	6	0	3.447711	-0.431585	1.211846
12	6	0	4.508802	-1.627339	-1.078891
13	1	0	3.197006	-0.302131	-2.178010
14	6	0	4.419861	-1.419352	1.327138
15	1	0	3.034298	0.066216	2.080709
16	6	0	4.944341	-2.016839	0.185020
17	1	0	4.929749	-2.087328	-1.964519
18	1	0	4.772153	-1.717157	2.307158
19	1	0	5.702898	-2.784734	0.279555
20	6	0	-3.597534	-1.039734	-0.398658
21	8	0	-4.484656	-0.436673	-0.944159
22	8	0	-3.775748	-2.199581	0.240761
23	6	0	-5.110683	-2.712697	0.206174
24	1	0	-5.082643	-3.644329	0.763970
25	1	0	-5.798467	-2.004343	0.668436
26	1	0	-5.419913	-2.887264	-0.824271
27	7	0	0.289226	0.416376	-0.325435
28	1	0	-2.004591	0.255726	-1.029847
29	6	0	-2.367867	0.652603	1.244429
30	6	0	-1.437540	1.676737	1.057384
31	1	0	-3.414348	0.903779	1.108696
32	1	0	-2.159297	-0.027587	2.059040
33	1	0	-0.486335	1.728094	1.570918
34	8	0	-1.621891	2.711102	0.275198
35	6	0	-2.886897	2.892573	-0.367114
36	1	0	-3.639469	3.157238	0.380206
37	1	0	-2.744330	3.716740	-1.059239
38	1	0	-3.204955	1.999115	-0.904751

TS17 (NIMAG=1; 494.5i cm^{-1})

Center Number	Atomic Number	Atomic Type	Coordinates (Angstroms) X	Y	Z
1	6	0	1.917209	0.737615	0.213907
2	6	0	1.276187	0.245354	1.335019
3	6	0	0.065935	-0.419627	1.149934

4	1	0	1.795073	0.197543	2.284599
5	1	0	-0.391519	-0.997003	1.950457
6	16	0	-1.903452	-1.267600	-0.257055
7	8	0	-2.133694	-2.193619	0.839086
8	8	0	-1.961332	-1.721502	-1.631942
9	6	0	-3.027111	0.101240	-0.068134
10	6	0	-3.253530	0.933669	-1.157359
11	6	0	-3.640317	0.305741	1.160326
12	6	0	-4.120954	2.007146	-1.003981
13	1	0	-2.770458	0.723924	-2.103825
14	6	0	-4.506666	1.384331	1.299324
15	1	0	-3.451607	-0.381620	1.976021
16	6	0	-4.742222	2.232126	0.221867
17	1	0	-4.316503	2.665435	-1.841426
18	1	0	-5.001301	1.558355	2.246898
19	1	0	-5.419329	3.070292	0.335227
20	6	0	3.269386	1.335286	0.335338
21	8	0	3.943250	1.370905	1.332341
22	8	0	3.681908	1.827527	-0.846178
23	6	0	4.982887	2.419543	-0.834577
24	1	0	5.160135	2.763921	-1.849568
25	1	0	5.011342	3.253072	-0.132763
26	1	0	5.731371	1.683662	-0.538728
27	7	0	-0.417555	-0.472996	-0.089033
28	1	0	1.337891	1.052961	-0.642941
29	6	0	2.194080	-1.223978	-0.838538
30	6	0	0.936775	-1.382347	-1.394182
31	1	0	3.000530	-0.709677	-1.353684
32	1	0	0.442631	-2.333962	-1.254349
33	1	0	0.661648	-0.810257	-2.267927
34	8	0	2.512904	-2.115618	0.124947
35	6	0	3.795033	-1.957397	0.711989
36	1	0	3.821308	-1.074438	1.353439
37	1	0	3.977151	-2.850447	1.305121
38	1	0	4.565310	-1.872942	-0.062060

TS18 (NIMAG=1; 488.6*i* cm^{-1})

Center Number	Atomic Number	Atomic Type	Coordinates (Angstroms) X	Y	Z
1	6	0	-2.308484	-0.531969	0.482782
2	6	0	-1.363206	-0.120633	1.399091
3	6	0	-0.026192	-0.424997	1.138661
4	1	0	-1.629807	0.576027	2.184568
5	1	0	0.764393	0.019122	1.741486
6	16	0	1.825599	-1.613371	-0.234774
7	8	0	1.885764	-1.954668	-1.640105
8	8	0	2.146903	-2.577612	0.795818
9	6	0	2.872143	-0.189122	0.020994
10	6	0	3.402439	0.041749	1.285920
11	6	0	3.142217	0.655144	-1.050396
12	6	0	4.199665	1.163263	1.484339
13	1	0	3.212367	-0.667296	2.083079
14	6	0	3.942237	1.771211	-0.839264
15	1	0	2.762113	0.413327	-2.035766
16	6	0	4.461952	2.027753	0.426195
17	1	0	4.624368	1.355095	2.461974
18	1	0	4.169385	2.434025	-1.665045
19	1	0	5.086978	2.898362	0.584685
20	6	0	-3.725443	-0.124896	0.646535
21	8	0	-4.154352	0.630013	1.480904

22	8	0	-4.497799	-0.695502	-0.293307
23	6	0	-5.886451	-0.364032	-0.220630
24	1	0	-6.361744	-0.902604	-1.035563
25	1	0	-6.297638	-0.674446	0.739897
26	1	0	-6.025534	0.711733	-0.333218
27	7	0	0.236399	-1.120633	0.041257
28	1	0	-2.133226	-1.416737	-0.113680
29	6	0	-1.518951	0.650890	-1.285597
30	6	0	-0.488465	-0.181114	-1.676083
31	1	0	-2.534743	0.547402	-1.655515
32	1	0	0.479213	0.277106	-1.823893
33	1	0	-0.694679	-1.090402	-2.220555
34	8	0	-1.136344	1.845735	-0.780973
35	6	0	-2.187362	2.696301	-0.349144
36	1	0	-2.644288	2.314211	0.565653
37	1	0	-1.741390	3.670081	-0.160511
38	1	0	-2.950110	2.792335	-1.129128

TS19 (NIMAG=1; 487.4i cm^{-1})

Center Number	Atomic Number	Atomic Type	Coordinates (Angstroms) X	Y	Z
1	6	0	-1.892938	0.737365	0.257960
2	6	0	-1.347061	0.557275	-1.000313
3	6	0	-0.130334	-0.117430	-1.089961
4	1	0	-1.933819	0.739349	-1.891291
5	1	0	0.254353	-0.461837	-2.047777
6	16	0	1.934603	-1.284835	-0.117751
7	8	0	2.071152	-1.874577	-1.439185
8	8	0	2.086216	-2.096674	1.072578
9	6	0	3.059632	0.091441	-0.008601
10	6	0	3.368501	0.602049	1.246259
11	6	0	3.591501	0.622517	-1.175639
12	6	0	4.236427	1.682859	1.328390
13	1	0	2.947397	0.142981	2.132315
14	6	0	4.459739	1.704195	-1.078185
15	1	0	3.340148	0.178428	-2.131159
16	6	0	4.777048	2.232814	0.168806
17	1	0	4.495134	2.093276	2.296633
18	1	0	4.892158	2.129432	-1.975432
19	1	0	5.455170	3.074873	0.239084
20	6	0	-3.248507	1.298664	0.482122
21	8	0	-3.797816	1.313446	1.553849
22	8	0	-3.821961	1.765917	-0.640371
23	6	0	-5.152269	2.261018	-0.476090
24	1	0	-5.459263	2.614142	-1.456614
25	1	0	-5.810803	1.463609	-0.127953
26	1	0	-5.167263	3.073266	0.250277
27	7	0	0.451578	-0.484589	0.047358
28	1	0	-1.238892	0.822697	1.115318
29	6	0	-2.115895	-1.402196	0.844180
30	6	0	-0.813651	-1.725162	1.179878
31	1	0	-2.822038	-1.006390	1.568957
32	1	0	-0.376401	-2.605118	0.728243
33	1	0	-0.416766	-1.414088	2.134672
34	8	0	-2.611480	-2.028105	-0.249805
35	6	0	-3.935220	-1.672209	-0.603505
36	1	0	-4.586858	-1.671353	0.276616
37	1	0	-3.954741	-0.684608	-1.073441
38	1	0	-4.282931	-2.418415	-1.314264

TS20 (NIMAG=1; 479.8i cm^{-1})

Center Number	Atomic Number	Atomic Type	Coordinates (Angstroms) X	Y	Z
1	6	0	-2.297287	-0.760190	0.206704
2	6	0	-1.456099	-0.278168	1.189545
3	6	0	-0.084675	-0.499692	1.043646
4	1	0	-1.817198	0.413505	1.939637
5	1	0	0.622136	0.002639	1.702791
6	16	0	1.947864	-1.570078	-0.158969
7	8	0	2.138527	-1.934726	-1.546416
8	8	0	2.256706	-2.486735	0.918034
9	6	0	2.870129	-0.069441	0.136004
10	6	0	3.281945	0.228031	1.430820
11	6	0	3.168394	0.764535	-0.936061
12	6	0	3.984131	1.406374	1.657085
13	1	0	3.078400	-0.473432	2.231362
14	6	0	3.872874	1.937902	-0.696695
15	1	0	2.884797	0.472284	-1.940121
16	6	0	4.271603	2.260695	0.597195
17	1	0	4.316613	1.650565	2.658484
18	1	0	4.120849	2.594057	-1.521803
19	1	0	4.822917	3.175689	0.777694
20	6	0	-3.750374	-0.461234	0.164495
21	8	0	-4.459842	-0.719707	-0.773532
22	8	0	-4.196185	0.159500	1.270439
23	6	0	-5.580752	0.512505	1.246092
24	1	0	-5.783945	0.977620	2.206675
25	1	0	-5.777988	1.208496	0.429082
26	1	0	-6.195727	-0.376514	1.108491
27	7	0	0.312009	-1.186407	-0.016145
28	1	0	-2.010280	-1.635454	-0.360488
29	6	0	-1.488325	0.405374	-1.547674
30	6	0	-0.330346	-0.298363	-1.810064
31	1	0	-2.445166	0.148932	-1.993244
32	1	0	0.589090	0.268501	-1.859728
33	1	0	-0.369817	-1.232213	-2.350197
34	8	0	-1.319453	1.669705	-1.088630
35	6	0	-2.512634	2.363136	-0.771842
36	1	0	-3.260782	2.232618	-1.560644
37	1	0	-2.920657	2.009893	0.179638
38	1	0	-2.252689	3.415832	-0.685748

TS21 (NIMAG=1; 532.8i cm^{-1})

Center Number	Atomic Number	Atomic Type	Coordinates (Angstroms) X	Y	Z
1	6	0	-2.024655	-0.851262	0.188372
2	6	0	-1.317611	-0.449016	1.314349
3	6	0	-0.083291	0.158098	1.132714
4	1	0	-1.819875	-0.407960	2.272431
5	1	0	0.417176	0.662217	1.958181
6	16	0	1.804631	1.156689	-0.277002
7	8	0	1.972485	2.092465	0.825478
8	8	0	1.832258	1.627543	-1.647545
9	6	0	3.013905	-0.136531	-0.095483
10	6	0	3.262357	-0.972017	-1.177945
11	6	0	3.668463	-0.283790	1.119892
12	6	0	4.194754	-1.990143	-1.030321

13	1	0	2.744137	-0.807844	-2.114786
14	6	0	4.600570	-1.307091	1.252580
15	1	0	3.459982	0.405064	1.929465
16	6	0	4.858580	-2.157531	0.182522
17	1	0	4.407665	-2.650092	-1.862159
18	1	0	5.128297	-1.435748	2.189461
19	1	0	5.586417	-2.952659	0.291247
20	6	0	-3.427040	-1.312721	0.379207
21	8	0	-4.030650	-1.290127	1.415964
22	8	0	-3.958426	-1.741350	-0.780075
23	6	0	-5.318547	-2.173672	-0.694807
24	1	0	-5.594475	-2.482421	-1.699254
25	1	0	-5.405667	-3.006521	0.003130
26	1	0	-5.952997	-1.356062	-0.351774
27	7	0	0.361751	0.293560	-0.115680
28	1	0	-1.497457	-1.234643	-0.675806
29	6	0	-2.344050	1.007084	-0.817202
30	6	0	-1.080783	1.264756	-1.336236
31	1	0	-3.061115	0.462854	-1.419989
32	1	0	-0.593474	2.218079	-1.189581
33	1	0	-0.777429	0.717905	-2.216790
34	8	0	-2.950399	1.781566	0.119096
35	6	0	-2.211526	2.878435	0.636616
36	1	0	-2.093236	3.656855	-0.124394
37	1	0	-2.796757	3.267610	1.466148
38	1	0	-1.226436	2.573055	0.997363

TS22 (NIMAG=1; 524.8*i* cm^{-1})

Center Number	Atomic Number	Atomic Type	Coordinates (Angstroms) X	Y	Z
1	6	0	-2.390791	-0.616521	0.395901
2	6	0	-1.480204	-0.442826	1.426803
3	6	0	-0.160246	-0.824664	1.217943
4	1	0	-1.752901	0.160796	2.283132
5	1	0	0.599486	-0.572820	1.956608
6	16	0	1.779046	-1.718495	-0.228836
7	8	0	1.868764	-2.062966	-1.631075
8	8	0	2.184552	-2.634293	0.817420
9	6	0	2.694148	-0.199209	0.006442
10	6	0	3.105533	0.156683	1.287119
11	6	0	2.964072	0.606838	-1.094919
12	6	0	3.752570	1.373560	1.471428
13	1	0	2.948584	-0.528062	2.112089
14	6	0	3.617535	1.817943	-0.898880
15	1	0	2.690017	0.265394	-2.086538
16	6	0	3.996285	2.206301	0.383285
17	1	0	4.079124	1.664094	2.462341
18	1	0	3.842160	2.451874	-1.747980
19	1	0	4.504184	3.151618	0.531892
20	6	0	-3.747534	-0.026905	0.563138
21	8	0	-4.094494	0.664898	1.480246
22	8	0	-4.545136	-0.345349	-0.471192
23	6	0	-5.864371	0.202321	-0.405446
24	1	0	-6.373685	-0.145166	-1.299922
25	1	0	-6.373743	-0.150911	0.491211
26	1	0	-5.819346	1.291420	-0.382071
27	7	0	0.159455	-1.323475	0.030626
28	1	0	-2.300479	-1.463206	-0.272451
29	6	0	-1.590080	0.665263	-1.146563
30	6	0	-0.502472	-0.085468	-1.566753

31	1	0	-2.551255	0.531790	-1.627242
32	1	0	0.477749	0.361712	-1.634487
33	1	0	-0.682093	-0.937728	-2.205502
34	8	0	-1.487304	1.879044	-0.544346
35	6	0	-0.181597	2.360479	-0.267946
36	1	0	0.349178	2.602314	-1.195766
37	1	0	-0.314694	3.265242	0.320261
38	1	0	0.408658	1.638437	0.303666

TS23 (NIMAG=1; 531.9i cm^{-1})

Center Number	Atomic Number	Atomic Type	Coordinates (Angstroms) X	Y	Z
1	6	0	1.997818	-0.892962	0.276258
2	6	0	1.403432	-0.699167	-0.965693
3	6	0	0.171666	-0.059977	-1.005927
4	1	0	1.976848	-0.830961	-1.872966
5	1	0	-0.244955	0.286554	-1.950608
6	16	0	-1.808928	1.192240	0.021561
7	8	0	-1.870363	1.891131	-1.254063
8	8	0	-1.935125	1.926159	1.264867
9	6	0	-3.025057	-0.106940	0.001659
10	6	0	-3.367559	-0.715954	1.203083
11	6	0	-3.591423	-0.484004	-1.208372
12	6	0	-4.305537	-1.739414	1.184631
13	1	0	-2.916051	-0.376260	2.127179
14	6	0	-4.530580	-1.509496	-1.211099
15	1	0	-3.311041	0.034020	-2.117437
16	6	0	-4.882104	-2.135469	-0.019716
17	1	0	-4.590645	-2.225074	2.109630
18	1	0	-4.990880	-1.814798	-2.142615
19	1	0	-5.614975	-2.933360	-0.027606
20	6	0	3.394759	-1.390559	0.405149
21	8	0	3.921301	-1.628824	1.460967
22	8	0	4.016806	-1.506604	-0.773783
23	6	0	5.383433	-1.917202	-0.696280
24	1	0	5.735793	-1.965092	-1.722805
25	1	0	5.960011	-1.191218	-0.122758
26	1	0	5.458619	-2.892171	-0.214611
27	7	0	-0.375718	0.307294	0.151304
28	1	0	1.376746	-1.102078	1.137953
29	6	0	2.274475	1.110061	0.952596
30	6	0	0.978575	1.472172	1.300830
31	1	0	2.922685	0.674954	1.704191
32	1	0	0.528825	2.386679	0.941631
33	1	0	0.587658	1.097477	2.235343
34	8	0	2.978279	1.694571	-0.053147
35	6	0	2.316874	2.693846	-0.814862
36	1	0	1.358677	2.342029	-1.205086
37	1	0	2.154208	3.593784	-0.212721
38	1	0	2.981544	2.927568	-1.643080

TS24 (NIMAG=1; 522.8i cm^{-1})

Center Number	Atomic Number	Atomic Type	Coordinates (Angstroms) X	Y	Z
1	6	0	-2.400256	-0.801031	0.120709
2	6	0	-1.602162	-0.491372	1.212848
3	6	0	-0.249451	-0.808511	1.150854

4	1	0	-1.971503	0.158956	1.993919
5	1	0	0.424319	-0.455072	1.930288
6	16	0	1.861067	-1.693419	-0.037131
7	8	0	2.099865	-2.147962	-1.389336
8	8	0	2.214755	-2.490976	1.118995
9	6	0	2.668075	-0.106572	0.141810
10	6	0	2.939489	0.379896	1.416847
11	6	0	2.996468	0.618856	-0.999000
12	6	0	3.501603	1.644930	1.548106
13	1	0	2.743448	-0.241678	2.282472
14	6	0	3.563986	1.879585	-0.855057
15	1	0	2.834060	0.179313	-1.976470
16	6	0	3.800988	2.396573	0.415696
17	1	0	3.719618	2.036691	2.534000
18	1	0	3.832893	2.452237	-1.734286
19	1	0	4.242681	3.379929	0.523453
20	6	0	-3.810407	-0.333588	0.024661
21	8	0	-4.544243	-0.617301	-0.885814
22	8	0	-4.163756	0.466625	1.037319
23	6	0	-5.492359	0.987652	0.957786
24	1	0	-5.621287	1.603441	1.843433
25	1	0	-5.607026	1.583147	0.051881
26	1	0	-6.217045	0.173576	0.942924
27	7	0	0.205401	-1.377219	0.042118
28	1	0	-2.187981	-1.688747	-0.461446
29	6	0	-1.529920	0.401301	-1.443037
30	6	0	-0.369931	-0.312001	-1.705335
31	1	0	-2.437542	0.172630	-1.988254
32	1	0	0.587974	0.185290	-1.725920
33	1	0	-0.447415	-1.220382	-2.284647
34	8	0	-1.544626	1.661880	-0.931873
35	6	0	-0.293928	2.244425	-0.601672
36	1	0	0.287156	1.611336	0.075305
37	1	0	0.296278	2.439190	-1.504227
38	1	0	-0.522662	3.186404	-0.108778

TS25 (NIMAG=1; 546.9*i* cm^{-1})

Center Number	Atomic Number	Atomic Type	Coordinates (Angstroms) X	Y	Z
1	6	0	-1.892209	0.490285	-0.620244
2	6	0	-1.134819	0.296535	-1.765369
3	6	0	0.138099	-0.244103	-1.609329
4	1	0	-1.585956	0.383736	-2.745832
5	1	0	0.739214	-0.538943	-2.467998
6	16	0	2.030049	-1.296616	-0.239507
7	8	0	2.480166	-1.822215	-1.518769
8	8	0	1.970252	-2.154283	0.926949
9	6	0	3.025310	0.126016	0.154169
10	6	0	2.982643	0.630508	1.448591
11	6	0	3.808433	0.698122	-0.838770
12	6	0	3.750318	1.746933	1.751297
13	1	0	2.370495	0.142427	2.197128
14	6	0	4.573403	1.815043	-0.520401
15	1	0	3.825289	0.259102	-1.828866
16	6	0	4.540857	2.337436	0.768222
17	1	0	3.735709	2.154268	2.754642
18	1	0	5.197781	2.272898	-1.277619
19	1	0	5.139406	3.207307	1.010965
20	6	0	-3.302527	0.938026	-0.767506
21	8	0	-4.018274	0.673793	-1.698076

22	8	0	-3.697912	1.665798	0.287457
23	6	0	-5.047644	2.129315	0.228119
24	1	0	-5.204260	2.701059	1.138578
25	1	0	-5.194932	2.754565	-0.652620
26	1	0	-5.737535	1.285124	0.177530
27	7	0	0.502546	-0.583325	-0.376735
28	1	0	-1.395228	0.754357	0.304390
29	6	0	-2.184150	-1.385054	0.397163
30	6	0	-0.941283	-1.991706	0.315986
31	1	0	-2.973243	-1.645204	-0.303567
32	1	0	-0.731402	-2.628549	-0.531317
33	1	0	-0.381707	-2.170725	1.223493
34	8	0	-2.589776	-0.893745	1.608322
35	6	0	-3.975948	-1.033306	1.860745
36	1	0	-4.221635	-2.072300	2.101742
37	1	0	-4.209234	-0.393011	2.708892
38	1	0	-4.569915	-0.716042	0.996632

TS26 (NIMAG=1; 537.6*i* cm^{-1})

Center Number	Atomic Number	Atomic Type	Coordinates (Angstroms) X	Y	Z
1	6	0	2.079286	-0.737903	0.379768
2	6	0	1.045749	-1.651519	0.267915
3	6	0	-0.234805	-1.223797	0.613165
4	1	0	1.199033	-2.597759	-0.235862
5	1	0	-1.094259	-1.873663	0.448684
6	16	0	-1.914277	0.588081	1.372796
7	8	0	-1.853313	2.032572	1.314506
8	8	0	-2.290407	-0.111343	2.583342
9	6	0	-3.018558	0.047020	0.076438
10	6	0	-3.698558	-1.156962	0.221869
11	6	0	-3.178789	0.846936	-1.050291
12	6	0	-4.539166	-1.580032	-0.802046
13	1	0	-3.586275	-1.726917	1.136739
14	6	0	-4.022129	0.413115	-2.065717
15	1	0	-2.672402	1.803553	-1.105448
16	6	0	-4.694046	-0.800279	-1.943660
17	1	0	-5.080577	-2.512747	-0.702738
18	1	0	-4.165489	1.027656	-2.945945
19	1	0	-5.353086	-1.132288	-2.736848
20	6	0	3.427345	-1.124497	-0.112510
21	8	0	3.639578	-1.888014	-1.018593
22	8	0	4.395536	-0.495787	0.568895
23	6	0	5.726259	-0.808388	0.154776
24	1	0	6.380526	-0.233748	0.804382
25	1	0	5.914702	-1.876727	0.261586
26	1	0	5.874690	-0.529092	-0.889850
27	7	0	-0.373675	0.051807	0.943961
28	1	0	2.039978	0.017714	1.153912
29	6	0	1.687244	0.995614	-0.889504
30	6	0	0.338460	1.222059	-0.677847
31	1	0	2.028530	0.435424	-1.756042
32	1	0	-0.366272	0.741794	-1.342222
33	1	0	0.032630	2.132817	-0.179412
34	8	0	2.590940	1.850638	-0.326974
35	6	0	3.755899	2.068409	-1.102028
36	1	0	3.539681	2.734417	-1.943203
37	1	0	4.490597	2.529688	-0.445603
38	1	0	4.158328	1.124756	-1.485960

TS27 (NIMAG=1; 526.4i cm^{-1})

Center Number	Atomic Number	Atomic Type	Coordinates (Angstroms) X	Y	Z
1	6	0	1.979107	-0.651428	0.034667
2	6	0	1.348699	-1.108141	-1.114502
3	6	0	0.074042	-0.640102	-1.417082
4	1	0	1.896671	-1.687355	-1.846055
5	1	0	-0.420267	-0.901808	-2.350984
6	16	0	-1.965037	0.866168	-1.048947
7	8	0	-2.303643	0.504158	-2.416036
8	8	0	-2.034390	2.248373	-0.619074
9	6	0	-2.961238	-0.118840	0.049001
10	6	0	-2.982004	0.208395	1.399959
11	6	0	-3.683005	-1.185608	-0.467168
12	6	0	-3.752950	-0.564350	2.257291
13	1	0	-2.412147	1.057217	1.758074
14	6	0	-4.453139	-1.949248	0.403831
15	1	0	-3.648065	-1.393006	-1.529696
16	6	0	-4.484503	-1.640338	1.759341
17	1	0	-3.786508	-0.326165	3.313196
18	1	0	-5.030744	-2.781936	0.021911
19	1	0	-5.086071	-2.238509	2.433275
20	6	0	3.380392	-1.066367	0.308309
21	8	0	3.861382	-1.212625	1.402085
22	8	0	4.080369	-1.252595	-0.823821
23	6	0	5.429164	-1.686982	-0.636689
24	1	0	5.847244	-1.789139	-1.634210
25	1	0	5.986486	-0.950572	-0.057243
26	1	0	5.449528	-2.640504	-0.108757
27	7	0	-0.419341	0.311901	-0.630666
28	1	0	1.383988	-0.429088	0.913240
29	6	0	2.023942	1.588992	0.059486
30	6	0	0.938674	1.903500	-0.739948
31	1	0	3.025647	1.502027	-0.352793
32	1	0	1.059815	1.851258	-1.813253
33	1	0	0.188356	2.577759	-0.349895
34	8	0	1.911502	1.904677	1.371744
35	6	0	3.086362	1.786529	2.163695
36	1	0	3.959517	2.158027	1.617169
37	1	0	2.926593	2.406721	3.043296
38	1	0	3.252908	0.751101	2.460206

TS28 (NIMAG=1; 516.1i cm^{-1})

Center Number	Atomic Number	Atomic Type	Coordinates (Angstroms) X	Y	Z
1	6	0	2.211759	0.255975	-0.684224
2	6	0	1.200866	1.164036	-0.954583
3	6	0	-0.111032	0.710387	-1.065569
4	1	0	1.399198	2.227401	-0.922596
5	1	0	-0.925394	1.422790	-1.195989
6	16	0	-1.923769	-1.121221	-0.934225
7	8	0	-1.968800	-2.385451	-0.232808
8	8	0	-2.281162	-1.012697	-2.332763
9	6	0	-2.943822	0.031777	-0.027105
10	6	0	-3.567998	1.069391	-0.709893
11	6	0	-3.092351	-0.135563	1.345815
12	6	0	-4.342297	1.974349	0.008412

13	1	0	-3.464044	1.138368	-1.786404
14	6	0	-3.868021	0.775550	2.051689
15	1	0	-2.630035	-0.983425	1.837513
16	6	0	-4.485601	1.830357	1.384527
17	1	0	-4.840823	2.784946	-0.508676
18	1	0	-4.001844	0.655107	3.119683
19	1	0	-5.092892	2.535532	1.939211
20	6	0	3.593758	0.752449	-0.455136
21	8	0	4.601684	0.143819	-0.703953
22	8	0	3.604103	1.982583	0.088397
23	6	0	4.903892	2.538464	0.299289
24	1	0	4.738071	3.519841	0.734928
25	1	0	5.479952	1.907126	0.976233
26	1	0	5.438292	2.620432	-0.647287
27	7	0	-0.333963	-0.565963	-0.784564
28	1	0	2.137048	-0.755460	-1.067336
29	6	0	1.708436	-0.942519	1.181114
30	6	0	0.326251	-0.880097	1.173120
31	1	0	2.310575	-0.213079	1.716095
32	1	0	-0.146499	0.006926	1.573381
33	1	0	-0.217945	-1.815770	1.202817
34	8	0	2.256009	-2.154118	0.943947
35	6	0	3.663791	-2.274694	1.104077
36	1	0	4.007568	-1.685919	1.960985
37	1	0	3.861863	-3.328117	1.290559
38	1	0	4.184503	-1.947686	0.204664

TS29 (NIMAG=1; 545.7*i* cm^{-1})

Center Number	Atomic Number	Atomic Type	Coordinates (Angstroms) X	Y	Z
1	6	0	-2.046264	0.523812	-0.558607
2	6	0	-1.288251	0.480428	-1.722424
3	6	0	-0.007840	-0.050857	-1.636461
4	1	0	-1.748973	0.674920	-2.682882
5	1	0	0.591463	-0.242879	-2.524934
6	16	0	1.887579	-1.242900	-0.379077
7	8	0	2.312600	-1.710903	-1.688323
8	8	0	1.848957	-2.149445	0.753131
9	6	0	2.892822	0.159541	0.060912
10	6	0	2.930566	0.563384	1.389997
11	6	0	3.601862	0.819318	-0.933670
12	6	0	3.701987	1.667555	1.727533
13	1	0	2.381693	0.003814	2.137648
14	6	0	4.370369	1.923141	-0.581145
15	1	0	3.562055	0.455983	-1.953412
16	6	0	4.416206	2.346145	0.743275
17	1	0	3.750739	1.995931	2.758386
18	1	0	4.937386	2.448142	-1.339870
19	1	0	5.017714	3.206074	1.012497
20	6	0	-3.471336	0.939694	-0.645920
21	8	0	-4.173391	0.775717	-1.609513
22	8	0	-3.887614	1.500220	0.494145
23	6	0	-5.267597	1.870510	0.515088
24	1	0	-5.441885	2.304393	1.495580
25	1	0	-5.477470	2.594454	-0.272556
26	1	0	-5.893823	0.990674	0.365257
27	7	0	0.357092	-0.535845	-0.448994
28	1	0	-1.543748	0.720816	0.380066
29	6	0	-2.286169	-1.449669	0.253985
30	6	0	-1.065211	-2.053899	-0.028486

31	1	0	-3.127444	-1.604436	-0.410226
32	1	0	-0.951282	-2.494804	-1.009379
33	1	0	-0.436853	-2.458237	0.752620
34	8	0	-2.699991	-1.143678	1.512717
35	6	0	-1.684697	-1.070879	2.501224
36	1	0	-0.819304	-0.509031	2.134986
37	1	0	-2.126382	-0.561733	3.354539
38	1	0	-1.357964	-2.071408	2.800808

TS30 (NIMAG=1; 538.3i cm^{-1})

Center Number	Atomic Number	Atomic Type	Coordinates (Angstroms) X	Y	Z
1	6	0	-2.200751	-0.615708	-0.506837
2	6	0	-1.169845	-1.488667	-0.822806
3	6	0	0.101608	-0.954070	-0.998417
4	1	0	-1.321219	-2.558988	-0.757116
5	1	0	0.962838	-1.605260	-1.146282
6	16	0	1.749355	1.049458	-0.963593
7	8	0	1.676835	2.322225	-0.273763
8	8	0	2.068566	0.971884	-2.373681
9	6	0	2.917663	0.022746	-0.087064
10	6	0	3.600544	-0.969363	-0.781611
11	6	0	3.114022	0.232096	1.274032
12	6	0	4.486330	-1.785312	-0.086082
13	1	0	3.453355	-1.071554	-1.850383
14	6	0	4.001894	-0.589793	1.956823
15	1	0	2.599575	1.044573	1.773433
16	6	0	4.680535	-1.599020	1.278694
17	1	0	5.030946	-2.559390	-0.612469
18	1	0	4.173610	-0.435294	3.014928
19	1	0	5.374082	-2.235076	1.815429
20	6	0	-3.531435	-1.176472	-0.154773
21	8	0	-3.707929	-2.263363	0.332002
22	8	0	-4.512954	-0.310179	-0.422394
23	6	0	-5.822791	-0.749547	-0.057842
24	1	0	-6.493041	0.058365	-0.336958
25	1	0	-6.076493	-1.666073	-0.590901
26	1	0	-5.870260	-0.937719	1.015068
27	7	0	0.237985	0.344472	-0.746999
28	1	0	-2.188463	0.380805	-0.929904
29	6	0	-1.758053	0.469485	1.328326
30	6	0	-0.377924	0.617758	1.251944
31	1	0	-2.182833	-0.353873	1.889013
32	1	0	0.220209	-0.208946	1.612096
33	1	0	0.079919	1.597650	1.267382
34	8	0	-2.632920	1.498880	1.213927
35	6	0	-2.127567	2.686709	0.617706
36	1	0	-1.538415	2.461075	-0.275997
37	1	0	-2.995317	3.285872	0.353313
38	1	0	-1.502245	3.240588	1.324532

TS31 (NIMAG=1; 544.5i cm^{-1})

Center Number	Atomic Number	Atomic Type	Coordinates (Angstroms) X	Y	Z
1	6	0	2.039956	-0.665582	0.068864
2	6	0	1.403114	-1.163796	-1.063241
3	6	0	0.134956	-0.671921	-1.347815

4	1	0	1.939129	-1.760082	-1.789928
5	1	0	-0.371461	-0.908030	-2.282359
6	16	0	-1.836344	0.923143	-0.950224
7	8	0	-2.124812	0.733859	-2.362839
8	8	0	-1.872324	2.248196	-0.360632
9	6	0	-2.921826	-0.135408	-0.016612
10	6	0	-3.084331	0.107054	1.342047
11	6	0	-3.566057	-1.180697	-0.664286
12	6	0	-3.917367	-0.730586	2.071729
13	1	0	-2.581253	0.948884	1.802051
14	6	0	-4.397745	-2.011059	0.078814
15	1	0	-3.428163	-1.319292	-1.729747
16	6	0	-4.568611	-1.787799	1.441155
17	1	0	-4.062718	-0.556619	3.130704
18	1	0	-4.916010	-2.828052	-0.407740
19	1	0	-5.218486	-2.437437	2.015086
20	6	0	3.430219	-1.059240	0.418206
21	8	0	3.832615	-1.209537	1.537606
22	8	0	4.198490	-1.208502	-0.677078
23	6	0	5.548630	-1.597475	-0.412943
24	1	0	6.030803	-1.671397	-1.383769
25	1	0	6.041062	-0.849289	0.208540
26	1	0	5.570869	-2.557057	0.104066
27	7	0	-0.329849	0.283041	-0.542977
28	1	0	1.429441	-0.435676	0.933430
29	6	0	2.277423	1.448540	-0.070189
30	6	0	1.100429	1.858286	-0.688231
31	1	0	3.170235	1.302420	-0.665673
32	1	0	1.075271	1.827859	-1.768879
33	1	0	0.417591	2.549257	-0.214418
34	8	0	2.589414	1.730751	1.222745
35	6	0	1.504525	2.093998	2.061410
36	1	0	0.651275	1.423818	1.913462
37	1	0	1.869101	2.012322	3.082414
38	1	0	1.186810	3.122759	1.865322

TS32 (NIMAG=1; 535.6i cm^{-1})

Center Number	Atomic Number	Atomic Type	Coordinates (Angstroms) X	Y	Z
1	6	0	-2.261419	-0.239827	-0.650623
2	6	0	-1.299306	-1.163415	-1.037989
3	6	0	0.015712	-0.718565	-1.131076
4	1	0	-1.528842	-2.219488	-1.094059
5	1	0	0.825551	-1.420165	-1.330535
6	16	0	1.818039	1.124531	-0.848144
7	8	0	1.826597	2.321448	-0.031082
8	8	0	2.178398	1.163020	-2.249868
9	6	0	2.869075	-0.080785	-0.053590
10	6	0	3.491426	-1.052156	-0.829546
11	6	0	3.038440	-0.025669	1.325979
12	6	0	4.285363	-2.005092	-0.200422
13	1	0	3.371551	-1.033597	-1.906441
14	6	0	3.834480	-0.983308	1.941973
15	1	0	2.576276	0.773672	1.893177
16	6	0	4.450284	-1.973182	1.180474
17	1	0	4.782509	-2.765223	-0.790313
18	1	0	3.984847	-0.949789	3.014001
19	1	0	5.072919	-2.715310	1.665515
20	6	0	-3.676670	-0.631411	-0.426614
21	8	0	-4.620343	0.073703	-0.648881

22	8	0	-3.778410	-1.874229	0.083373
23	6	0	-5.117968	-2.318890	0.310507
24	1	0	-5.031967	-3.322693	0.717389
25	1	0	-5.620362	-1.656502	1.015790
26	1	0	-5.676971	-2.327656	-0.625455
27	7	0	0.248722	0.530780	-0.743431
28	1	0	-2.140730	0.787277	-0.971790
29	6	0	-1.801065	0.586296	1.296262
30	6	0	-0.413100	0.654432	1.265093
31	1	0	-2.287916	-0.265784	1.754397
32	1	0	0.124953	-0.240057	1.549950
33	1	0	0.104571	1.596816	1.383390
34	8	0	-2.612182	1.671749	1.279396
35	6	0	-2.026579	2.885236	0.826085
36	1	0	-1.426631	2.723143	-0.074422
37	1	0	-2.852913	3.557192	0.609001
38	1	0	-1.391032	3.322692	1.602130

RC1 (NIMAG=0)

Center Number	Atomic Number	Atomic Type	Coordinates (Angstroms) X	Y	Z
1	6	0	2.281046	-0.923640	-0.342546
2	6	0	1.706637	-0.209074	-1.309011
3	6	0	0.369015	0.364850	-1.153436
4	1	0	2.258386	0.013325	-2.215391
5	1	0	0.020187	1.060971	-1.920285
6	16	0	-1.805779	0.941702	-0.018246
7	8	0	-2.067967	1.710509	-1.226177
8	8	0	-1.742673	1.618681	1.264284
9	6	0	-2.982223	-0.382438	0.080402
10	6	0	-3.030081	-1.142012	1.244102
11	6	0	-3.820070	-0.622014	-0.999755
12	6	0	-3.948124	-2.180147	1.318820
13	1	0	-2.367500	-0.909722	2.068945
14	6	0	-4.737700	-1.662943	-0.907503
15	1	0	-3.754369	0.006194	-1.879509
16	6	0	-4.797554	-2.438617	0.245256
17	1	0	-4.005212	-2.784699	2.215406
18	1	0	-5.406758	-1.864767	-1.734717
19	1	0	-5.514201	-3.248491	0.311153
20	6	0	3.676100	-1.408359	-0.511135
21	8	0	4.343350	-1.276160	-1.501234
22	8	0	4.107943	-2.010747	0.604702
23	6	0	5.450672	-2.496658	0.545828
24	1	0	5.643993	-2.949097	1.514278
25	1	0	5.550885	-3.232057	-0.252864
26	1	0	6.140961	-1.673912	0.357663
27	7	0	-0.355143	0.076980	-0.140840
28	1	0	1.772115	-1.136471	0.588836
29	6	0	2.786251	1.453352	1.659342
30	6	0	1.695528	2.058565	1.203872
31	1	0	2.791502	1.018852	2.648737
32	1	0	3.679632	1.394775	1.049827
33	1	0	0.777174	2.151484	1.781627
34	8	0	1.631695	2.544924	-0.064108
35	6	0	0.712421	3.618466	-0.238595
36	1	0	1.138660	4.546410	0.151792
37	1	0	0.537054	3.718130	-1.308340
38	1	0	-0.234337	3.409339	0.267076

PR1 (NIMAG=0)

Center Number	Atomic Number	Atomic Type	Coordinates (Angstroms) X	Y	Z
1	6	0	-1.529257	0.448142	0.350455
2	6	0	-0.897104	0.584691	1.706327
3	6	0	0.392632	0.894242	1.771968
4	1	0	-1.510980	0.497051	2.593414
5	1	0	0.949052	1.019013	2.691009
6	16	0	2.324143	-0.094689	0.314681
7	8	0	2.957607	-0.312606	1.595125
8	8	0	3.068397	0.309317	-0.858446
9	6	0	1.390038	-1.551550	-0.104719
10	6	0	1.133329	-1.825248	-1.444716
11	6	0	0.840772	-2.315122	0.920336
12	6	0	0.287384	-2.881329	-1.761556
13	1	0	1.608966	-1.229388	-2.214567
14	6	0	-0.007893	-3.365018	0.588307
15	1	0	1.085664	-2.089534	1.950643
16	6	0	-0.289925	-3.640107	-0.746861
17	1	0	0.082819	-3.114589	-2.799149
18	1	0	-0.445556	-3.970358	1.372418
19	1	0	-0.952643	-4.459365	-0.998585
20	6	0	-3.040326	0.409518	0.434508
21	8	0	-3.685727	0.426348	1.445201
22	8	0	-3.586258	0.343105	-0.788275
23	6	0	-5.015695	0.299565	-0.813770
24	1	0	-5.290776	0.250846	-1.863597
25	1	0	-5.375720	-0.578839	-0.277938
26	1	0	-5.428416	1.193873	-0.346891
27	7	0	1.141240	1.064657	0.567857
28	1	0	-1.222060	-0.498453	-0.118568
29	6	0	-1.045585	1.614597	-0.522917
30	6	0	0.481617	1.665438	-0.615306
31	1	0	-1.466992	1.563040	-1.526241
32	1	0	-1.364455	2.553065	-0.063219
33	1	0	0.839328	1.109167	-1.489966
34	8	0	0.809918	3.012582	-0.748019
35	6	0	2.173705	3.259291	-1.046968
36	1	0	2.261395	4.331950	-1.207956
37	1	0	2.818962	2.954765	-0.219579
38	1	0	2.482811	2.717563	-1.946336

RC9 (NIMAG=0)

Center Number	Atomic Number	Atomic Type	Coordinates (Angstroms) X	Y	Z
1	6	0	2.409760	-0.775186	-0.134466
2	6	0	1.684674	-1.245154	-1.149516
3	6	0	0.345035	-0.741988	-1.458969
4	1	0	2.114041	-1.988942	-1.812586
5	1	0	-0.070118	-0.971548	-2.444821
6	16	0	-1.812736	0.549881	-1.162102
7	8	0	-2.096755	0.077264	-2.505785
8	8	0	-1.802673	1.966842	-0.858215
9	6	0	-2.911962	-0.266790	-0.030229
10	6	0	-3.240227	0.367494	1.160995
11	6	0	-3.410137	-1.519853	-0.365808
12	6	0	-4.090332	-0.283600	2.047123

13	1	0	-2.853878	1.358403	1.366290
14	6	0	-4.256768	-2.159072	0.530548
15	1	0	-3.152113	-1.966957	-1.318280
16	6	0	-4.591339	-1.543234	1.733587
17	1	0	-4.365828	0.195114	2.978545
18	1	0	-4.661207	-3.133687	0.287375
19	1	0	-5.254243	-2.045687	2.427679
20	6	0	3.793202	-1.291182	0.062947
21	8	0	4.299854	-2.177652	-0.570540
22	8	0	4.413186	-0.623675	1.041849
23	6	0	5.757720	-1.035784	1.297732
24	1	0	6.111920	-0.400980	2.104698
25	1	0	5.779999	-2.085732	1.590760
26	1	0	6.367997	-0.904307	0.403916
27	7	0	-0.310897	-0.040306	-0.618545
28	1	0	2.049688	-0.002187	0.536954
29	6	0	2.491318	2.435374	-0.875791
30	6	0	1.399181	2.477801	-0.121425
31	1	0	3.431524	2.080243	-0.471021
32	1	0	2.451086	2.775430	-1.900230
33	1	0	0.435351	2.821869	-0.488184
34	8	0	1.421694	2.076012	1.176180
35	6	0	0.173833	2.076626	1.851585
36	1	0	-0.436716	2.930086	1.543921
37	1	0	-0.367169	1.150452	1.644994
38	1	0	0.393527	2.149454	2.915655

PR9 (NIMAG=0)

Center Number	Atomic Number	Atomic Type	Coordinates (Angstroms) X	Y	Z
1	6	0	-2.629690	-0.318764	0.570413
2	6	0	-2.043603	0.813812	1.362424
3	6	0	-0.769964	1.175855	1.247992
4	1	0	-2.691600	1.355827	2.037929
5	1	0	-0.343690	2.013215	1.782684
6	16	0	1.259809	1.381757	-0.441711
7	8	0	1.446458	2.607097	0.303643
8	8	0	0.874927	1.400164	-1.839587
9	6	0	2.731279	0.389250	-0.309625
10	6	0	3.145348	-0.363635	-1.399381
11	6	0	3.416803	0.378885	0.900080
12	6	0	4.272331	-1.169327	-1.263686
13	1	0	2.596373	-0.303082	-2.331517
14	6	0	4.538508	-0.428602	1.022424
15	1	0	3.073820	0.999159	1.720092
16	6	0	4.959480	-1.205976	-0.055640
17	1	0	4.613143	-1.763306	-2.102590
18	1	0	5.088084	-0.450234	1.955339
19	1	0	5.835488	-1.835277	0.046106
20	6	0	-4.075335	-0.036245	0.213199
21	8	0	-4.774881	0.782752	0.740370
22	8	0	-4.508383	-0.856785	-0.756967
23	6	0	-5.880223	-0.689415	-1.123053
24	1	0	-6.066715	-1.415690	-1.909325
25	1	0	-6.525639	-0.874560	-0.264155
26	1	0	-6.053235	0.324463	-1.483980
27	7	0	0.157107	0.466407	0.437165
28	1	0	-2.654694	-1.223377	1.193568
29	6	0	-1.759616	-0.626709	-0.651185
30	6	0	-0.319639	-0.780435	-0.188511

31	1	0	-2.086610	-1.544110	-1.137408
32	1	0	-1.801297	0.193955	-1.372066
33	1	0	0.342149	-0.996014	-1.035682
34	8	0	-0.291546	-1.830673	0.731335
35	6	0	0.994042	-2.136748	1.235317
36	1	0	1.711962	-2.294415	0.420242
37	1	0	1.363456	-1.336857	1.882291
38	1	0	0.891372	-3.054769	1.810738

TS1′ (NIMAG=1; 316.1i cm^{-1})

Center Number	Atomic Number	Atomic Type	Coordinates (Angstroms) X	Y	Z
1	6	0	1.874600	-0.516466	1.829766
2	6	0	1.810568	-0.860924	0.485750
3	6	0	0.667187	-0.484575	-0.264270
4	1	0	0.641647	-0.723461	-1.329885
5	16	0	-1.484716	0.792493	-0.678554
6	8	0	-1.250227	0.463630	-2.079442
7	8	0	-1.661475	2.186201	-0.293732
8	6	0	-2.904274	-0.123370	-0.122872
9	6	0	-3.514150	0.248217	1.069478
10	6	0	-3.356955	-1.192736	-0.883525
11	6	0	-4.610046	-0.481005	1.511175
12	1	0	-3.141042	1.102788	1.620467
13	6	0	-4.456655	-1.912814	-0.429734
14	1	0	-2.861852	-1.434639	-1.816070
15	6	0	-5.077223	-1.559487	0.763933
16	1	0	-5.103715	-0.205313	2.434911
17	1	0	-4.830573	-2.746252	-1.011527
18	1	0	-5.934407	-2.123444	1.111917
19	7	0	-0.271986	0.215603	0.306617
20	1	0	0.934688	-0.373412	2.346793
21	1	0	2.715830	-0.862760	2.415876
22	6	0	2.963583	-1.375821	-0.264820
23	8	0	2.956008	-1.682623	-1.430427
24	8	0	4.071628	-1.462071	0.503236
25	6	0	5.229031	-1.954382	-0.171214
26	1	0	6.022164	-1.967278	0.571923
27	1	0	5.046306	-2.957757	-0.556659
28	1	0	5.490818	-1.300120	-1.003172
29	6	0	2.410731	1.504276	2.026036
30	6	0	1.732515	2.062458	0.975913
31	1	0	2.070951	1.718858	3.029500
32	1	0	3.459392	1.278275	1.877394
33	1	0	0.759677	2.540355	1.061745
34	8	0	2.229613	1.910125	-0.230279
35	6	0	1.502008	2.449782	-1.345952
36	1	0	2.129308	3.209645	-1.809467
37	1	0	1.316843	1.637439	-2.047401
38	1	0	0.553428	2.876948	-1.018631

TS2′ (NIMAG=1; 342.3i cm^{-1})

Center Number	Atomic Number	Atomic Type	Coordinates (Angstroms) X	Y	Z
1	6	0	2.528182	-0.912454	-1.384040
2	6	0	1.800701	0.179624	-0.909526
3	6	0	0.407855	0.025188	-0.687736

4	1	0	-0.149411	0.902292	-0.349344
5	16	0	-1.737237	-1.436777	-0.528882
6	8	0	-1.781979	-2.273741	0.664667
7	8	0	-2.325200	-1.908307	-1.763940
8	6	0	-2.512126	0.119807	-0.121957
9	6	0	-2.866772	0.976467	-1.158619
10	6	0	-2.720403	0.456141	1.209544
11	6	0	-3.434181	2.205420	-0.847177
12	1	0	-2.711257	0.668753	-2.186044
13	6	0	-3.294168	1.687346	1.508625
14	1	0	-2.464888	-0.254581	1.986060
15	6	0	-3.642884	2.560004	0.483079
16	1	0	-3.717793	2.884373	-1.641737
17	1	0	-3.474053	1.962062	2.540715
18	1	0	-4.086744	3.519281	0.720573
19	7	0	-0.130052	-1.150066	-0.820569
20	1	0	1.976009	-1.644220	-1.960773
21	1	0	3.563653	-0.754701	-1.656935
22	6	0	2.423695	1.405055	-0.404456
23	8	0	1.831711	2.348852	0.062434
24	8	0	3.770884	1.369145	-0.497718
25	6	0	4.436128	2.533610	-0.010980
26	1	0	4.212674	2.685811	1.045371
27	1	0	5.497765	2.351100	-0.156391
28	1	0	4.117693	3.414836	-0.568424
29	6	0	2.968467	-2.218530	0.125705
30	6	0	1.855084	-2.092797	0.919546
31	1	0	3.117741	-3.151444	-0.399869
32	1	0	3.846574	-1.661333	0.428935
33	1	0	1.001237	-2.765318	0.898005
34	8	0	1.757063	-1.004461	1.643744
35	6	0	0.566609	-0.791389	2.415050
36	1	0	0.812171	-0.945335	3.465672
37	1	0	0.272209	0.245062	2.250906
38	1	0	-0.224384	-1.470620	2.093541

TS3′ (NIMAG=1; 297.7i cm^{-1})

Center Number	Atomic Number	Atomic Type	Coordinates (Angstroms) X	Y	Z
1	6	0	1.796595	0.125400	2.134128
2	6	0	1.811373	-0.617545	0.963545
3	6	0	0.729599	-0.494848	0.052575
4	1	0	0.756855	-1.054964	-0.883570
5	16	0	-1.368701	0.572153	-0.886484
6	8	0	-1.049891	-0.190216	-2.088778
7	8	0	-1.550040	2.013445	-0.984841
8	6	0	-2.834523	-0.125908	-0.160415
9	6	0	-3.502497	0.596458	0.821448
10	6	0	-3.265799	-1.376643	-0.580452
11	6	0	-4.635508	0.040943	1.400583
12	1	0	-3.143522	1.578386	1.104445
13	6	0	-4.403453	-1.919607	0.006776
14	1	0	-2.724502	-1.895065	-1.362346
15	6	0	-5.082138	-1.214420	0.995039
16	1	0	-5.173875	0.588907	2.164046
17	1	0	-4.761431	-2.890930	-0.311532
18	1	0	-5.968483	-1.641809	1.448485
19	7	0	-0.230241	0.348853	0.308582
20	1	0	0.837326	0.443486	2.519480
21	1	0	2.602256	-0.028959	2.841495

22	6	0	3.064567	-1.282281	0.578666
23	8	0	4.089201	-1.228837	1.213300
24	8	0	2.960293	-1.957090	-0.582832
25	6	0	4.156385	-2.606555	-1.011920
26	1	0	3.906197	-3.097738	-1.948511
27	1	0	4.952291	-1.876114	-1.159638
28	1	0	4.480203	-3.335607	-0.268728
29	6	0	2.424751	2.101698	1.714845
30	6	0	1.808652	2.312765	0.513613
31	1	0	2.052819	2.625063	2.584330
32	1	0	3.464192	1.796646	1.700906
33	1	0	0.850512	2.813939	0.399794
34	8	0	2.342058	1.776467	-0.562580
35	6	0	1.678116	1.968679	-1.821334
36	1	0	2.342391	2.549848	-2.459444
37	1	0	1.501849	0.986765	-2.258683
38	1	0	0.726141	2.482758	-1.683600

TS4′ (NIMAG=1; 323.5i cm^{-1})

Center Number	Atomic Number	Atomic Type	Coordinates (Angstroms) X	Y	Z
1	6	0	2.678753	-0.873258	-1.413206
2	6	0	1.891232	0.191866	-0.983072
3	6	0	0.513314	-0.032829	-0.722770
4	1	0	-0.103301	0.820897	-0.434646
5	16	0	-1.538245	-1.610450	-0.466887
6	8	0	-1.537299	-2.402351	0.756994
7	8	0	-2.103225	-2.157145	-1.681601
8	6	0	-2.400103	-0.085280	-0.113978
9	6	0	-2.819791	0.704457	-1.179043
10	6	0	-2.614885	0.291710	1.205652
11	6	0	-3.460214	1.907497	-0.909604
12	1	0	-2.658202	0.363943	-2.195162
13	6	0	-3.261695	1.495944	1.463065
14	1	0	-2.308327	-0.370651	2.006191
15	6	0	-3.676356	2.302520	0.408082
16	1	0	-3.797201	2.532971	-1.726980
17	1	0	-3.449796	1.799308	2.485706
18	1	0	-4.180193	3.239539	0.612906
19	7	0	0.048867	-1.245562	-0.774854
20	1	0	2.177146	-1.685892	-1.922631
21	1	0	3.696394	-0.648657	-1.709003
22	6	0	2.556135	1.434961	-0.583055
23	8	0	3.747400	1.622345	-0.622212
24	8	0	1.688415	2.370037	-0.135435
25	6	0	2.287581	3.601418	0.264587
26	1	0	2.820588	4.054872	-0.571504
27	1	0	1.468038	4.238482	0.587246
28	1	0	2.990988	3.434410	1.080835
29	6	0	3.279243	-2.025945	0.184579
30	6	0	2.159978	-1.966046	0.972580
31	1	0	3.521710	-2.965838	-0.291211
32	1	0	4.091205	-1.351762	0.430238
33	1	0	1.378185	-2.721258	0.989630
34	8	0	1.947987	-0.855966	1.640881
35	6	0	0.748453	-0.743034	2.417004
36	1	0	1.012113	-0.847476	3.469543
37	1	0	0.351591	0.254865	2.231633
38	1	0	0.022813	-1.500869	2.117424

TS5′ (NIMAG=1; 381.5i cm^{-1})

Center Number	Atomic Number	Atomic Type	Coordinates (Angstroms) X	Y	Z
1	6	0	1.790423	-0.113542	1.799452
2	6	0	1.742095	-0.750106	0.547412
3	6	0	0.595718	-0.555177	-0.259269
4	1	0	0.575121	-0.978094	-1.265553
5	16	0	-1.547104	0.648485	-0.893024
6	8	0	-1.347058	0.042095	-2.200453
7	8	0	-1.661480	2.093746	-0.767589
8	6	0	-2.998488	-0.084926	-0.166250
9	6	0	-3.611042	0.556512	0.903465
10	6	0	-3.473270	-1.286465	-0.674645
11	6	0	-4.730170	-0.029383	1.480658
12	1	0	-3.222417	1.504967	1.253403
13	6	0	-4.595221	-1.861717	-0.088410
14	1	0	-2.976968	-1.741896	-1.522928
15	6	0	-5.218176	-1.236494	0.986878
16	1	0	-5.225926	0.458174	2.311151
17	1	0	-4.985339	-2.795068	-0.475366
18	1	0	-6.093347	-1.688044	1.438743
19	7	0	-0.358425	0.212893	0.187707
20	1	0	0.835171	0.019597	2.291733
21	1	0	2.635045	-0.329438	2.442133
22	6	0	2.882870	-1.450295	-0.041144
23	8	0	2.878591	-2.065920	-1.077209
24	8	0	4.008209	-1.311949	0.710042
25	6	0	5.152029	-1.997867	0.201363
26	1	0	5.954211	-1.803487	0.908811
27	1	0	4.953132	-3.067316	0.130275
28	1	0	5.412011	-1.624279	-0.789884
29	6	0	2.102126	1.865314	1.687114
30	6	0	1.447046	2.209831	0.518238
31	1	0	1.637188	2.198475	2.605441
32	1	0	3.184149	1.831831	1.695444
33	1	0	0.422358	2.562253	0.482447
34	8	0	1.941200	2.025101	-0.678386
35	6	0	3.341193	1.766500	-0.845163
36	1	0	3.680182	0.949949	-0.210849
37	1	0	3.461149	1.499913	-1.891161
38	1	0	3.898423	2.678767	-0.618836

TS6′ (NIMAG=1; 393.0i cm^{-1})

Center Number	Atomic Number	Atomic Type	Coordinates (Angstroms) X	Y	Z
1	6	0	2.421685	-1.340671	-1.049744
2	6	0	1.748212	-0.106740	-0.997475
3	6	0	0.333968	-0.121098	-0.926649
4	1	0	-0.179619	0.836215	-0.815861
5	16	0	-1.896261	-1.397461	-0.563693
6	8	0	-1.965272	-2.288328	0.586063
7	8	0	-2.597475	-1.732044	-1.785745
8	6	0	-2.465332	0.206677	-0.025469
9	6	0	-3.111265	1.035585	-0.932871
10	6	0	-2.213822	0.601097	1.284483
11	6	0	-3.521062	2.296557	-0.511614
12	1	0	-3.297273	0.681040	-1.939574

13	6	0	-2.627029	1.863089	1.691445
14	1	0	-1.713969	-0.080799	1.962381
15	6	0	-3.276813	2.707709	0.794316
16	1	0	-4.032448	2.955232	-1.202643
17	1	0	-2.448645	2.187129	2.709543
18	1	0	-3.597484	3.690866	1.117524
19	7	0	-0.274676	-1.268958	-0.889510
20	1	0	1.894294	-2.133797	-1.565851
21	1	0	3.496824	-1.319230	-1.179490
22	6	0	2.425560	1.173343	-0.808985
23	8	0	1.900066	2.259325	-0.802491
24	8	0	3.759646	1.018550	-0.598146
25	6	0	4.484586	2.237865	-0.439342
26	1	0	4.114851	2.792940	0.423773
27	1	0	5.522854	1.949677	-0.295776
28	1	0	4.378275	2.861244	-1.327253
29	6	0	2.419541	-2.323034	0.676490
30	6	0	1.189278	-2.005862	1.227079
31	1	0	2.527341	-3.342686	0.330711
32	1	0	3.313169	-1.875580	1.093109
33	1	0	0.299199	-2.613754	1.111272
34	8	0	0.936390	-0.879413	1.850064
35	6	0	2.014285	-0.040567	2.286352
36	1	0	2.726765	0.152495	1.487210
37	1	0	1.547686	0.890117	2.597287
38	1	0	2.513519	-0.517449	3.133240

TS7′ (NIMAG=1; 363.0*i* cm^{-1})

Center Number	Atomic Number	Atomic Type	Coordinates (Angstroms) X	Y	Z
1	6	0	-1.746102	-0.459861	1.953290
2	6	0	-1.771490	0.480502	0.912421
3	6	0	-0.672813	0.534607	0.019684
4	1	0	-0.702646	1.210935	-0.835564
5	16	0	1.433776	-0.404351	-1.034828
6	8	0	1.162696	0.546207	-2.103602
7	8	0	1.551365	-1.823364	-1.332428
8	6	0	2.924722	0.112366	-0.208809
9	6	0	3.584087	-0.793380	0.613422
10	6	0	3.383921	1.409480	-0.393033
11	6	0	4.734879	-0.381038	1.272438
12	1	0	3.205508	-1.803482	0.710601
13	6	0	4.538239	1.809070	0.271325
14	1	0	2.850157	2.076124	-1.059281
15	6	0	5.208031	0.917671	1.103094
16	1	0	5.266614	-1.073891	1.913046
17	1	0	4.916805	2.814613	0.134708
18	1	0	6.107976	1.233535	1.617201
19	7	0	0.308110	-0.307033	0.187891
20	1	0	-0.769666	-0.707153	2.348853
21	1	0	-2.568951	-0.432185	2.658453
22	6	0	-3.005425	1.223467	0.662887
23	8	0	-4.068379	0.989933	1.196172
24	8	0	-2.856446	2.196427	-0.254857
25	6	0	-4.038534	2.938445	-0.551667
26	1	0	-3.744976	3.675053	-1.294618
27	1	0	-4.813027	2.280423	-0.947683
28	1	0	-4.416938	3.425926	0.346976
29	6	0	-2.063685	-2.367454	1.352483
30	6	0	-1.493782	-2.366760	0.094752

31	1	0	-1.528054	-2.910664	2.119349
32	1	0	-3.141494	-2.350526	1.449828
33	1	0	-0.468685	-2.659712	-0.102258
34	8	0	-2.077918	-1.884770	-0.972033
35	6	0	-3.493027	-1.651344	-0.959574
36	1	0	-3.792502	-1.002896	-0.137868
37	1	0	-3.712287	-1.175730	-1.911181
38	1	0	-4.009701	-2.611611	-0.890846

TS8′ (NIMAG=1; 376.1*i* cm^{-1})

Center Number	Atomic Number	Atomic Type	Coordinates (Angstroms) X	Y	Z
1	6	0	2.578509	-1.261088	-1.122184
2	6	0	1.855838	-0.062195	-1.012331
3	6	0	0.441694	-0.129854	-0.938665
4	1	0	-0.115366	0.796101	-0.788513
5	16	0	-1.735571	-1.501550	-0.614960
6	8	0	-1.765167	-2.452192	0.486779
7	8	0	-2.437999	-1.792387	-1.847479
8	6	0	-2.353535	0.052462	0.013082
9	6	0	-3.061360	0.892988	-0.835398
10	6	0	-2.083145	0.395417	1.333887
11	6	0	-3.515664	2.112349	-0.342524
12	1	0	-3.260467	0.577474	-1.852577
13	6	0	-2.540491	1.616414	1.812581
14	1	0	-1.534264	-0.293947	1.964881
15	6	0	-3.253420	2.471765	0.975194
16	1	0	-4.077980	2.777511	-0.986278
17	1	0	-2.349525	1.898560	2.840882
18	1	0	-3.611494	3.421159	1.355350
19	7	0	-0.122629	-1.300288	-0.943842
20	1	0	2.091482	-2.059509	-1.667371
21	1	0	3.652675	-1.173463	-1.239237
22	6	0	2.579884	1.178602	-0.750838
23	8	0	3.757791	1.243523	-0.473453
24	8	0	1.793548	2.271137	-0.816399
25	6	0	2.451881	3.509168	-0.552693
26	1	0	3.258581	3.670658	-1.267832
27	1	0	1.688921	4.276291	-0.654685
28	1	0	2.869538	3.513281	0.454959
29	6	0	2.642764	-2.333173	0.571334
30	6	0	1.408600	-2.078926	1.138281
31	1	0	2.782036	-3.327867	0.169331
32	1	0	3.525936	-1.864650	0.986465
33	1	0	0.535428	-2.704496	0.991632
34	8	0	1.126455	-0.990444	1.815536
35	6	0	2.192101	-0.159562	2.297577
36	1	0	2.868745	0.141476	1.499755
37	1	0	1.704905	0.714203	2.721793
38	1	0	2.739433	-0.699260	3.074024

TS9′ (NIMAG=1; 315.4*i* cm^{-1})

Center Number	Atomic Number	Atomic Type	Coordinates (Angstroms) X	Y	Z
1	6	0	-2.229036	0.784397	-1.239419
2	6	0	-2.218709	-0.355598	-0.450659
3	6	0	-1.051365	-0.693264	0.280232

4	1	0	-1.093815	-1.551509	0.954181
5	16	0	1.242813	-0.298535	1.275929
6	8	0	0.954616	-1.493607	2.049750
7	8	0	1.564743	0.948164	1.956872
8	6	0	2.580776	-0.668894	0.157970
9	6	0	3.619791	0.239442	0.013493
10	6	0	2.557376	-1.876690	-0.530769
11	6	0	4.660728	-0.068313	-0.857679
12	1	0	3.612766	1.153937	0.594348
13	6	0	3.598619	-2.169678	-1.399895
14	1	0	1.743156	-2.574843	-0.374555
15	6	0	4.645959	-1.265329	-1.564222
16	1	0	5.484391	0.624586	-0.978211
17	1	0	3.598154	-3.105738	-1.944488
18	1	0	5.458212	-1.500956	-2.241329
19	7	0	0.004005	0.069039	0.213424
20	1	0	-1.296758	1.125230	-1.675102
21	1	0	-3.137583	1.024521	-1.776937
22	6	0	-3.434794	-1.132150	-0.159792
23	8	0	-3.468739	-2.145108	0.491861
24	8	0	-4.537916	-0.585977	-0.713299
25	6	0	-5.745628	-1.310187	-0.479057
26	1	0	-5.946313	-1.379342	0.590339
27	1	0	-6.529185	-0.749285	-0.981769
28	1	0	-5.669735	-2.317838	-0.888481
29	6	0	-2.278907	2.522356	-0.004500
30	6	0	-0.945979	2.642204	0.272203
31	1	0	-2.681038	3.108376	-0.821679
32	1	0	-2.952223	2.224742	0.786025
33	1	0	-0.489712	2.318977	1.203721
34	8	0	-0.139787	3.138661	-0.648515
35	6	0	1.268826	3.020938	-0.423001
36	1	0	1.493205	3.059676	0.643242
37	1	0	1.610784	2.069066	-0.831013
38	1	0	1.733372	3.851208	-0.949918

TS10′ (NIMAG=1; 302.8*i* cm^{-1})

Center Number	Atomic Number	Atomic Type	Coordinates (Angstroms) X	Y	Z
1	6	0	-2.726640	0.272584	-0.834561
2	6	0	-1.814302	-0.727984	-0.543252
3	6	0	-0.440507	-0.399139	-0.380797
4	1	0	0.238366	-1.213782	-0.118119
5	16	0	1.497174	1.293204	-0.092928
6	8	0	1.414160	1.908912	1.226012
7	8	0	1.990077	2.069280	-1.214548
8	6	0	2.501327	-0.171969	0.046880
9	6	0	3.065844	-0.705553	-1.106193
10	6	0	2.672011	-0.760456	1.293803
11	6	0	3.822413	-1.865736	-1.000927
12	1	0	2.924619	-0.203001	-2.055591
13	6	0	3.432506	-1.920658	1.384321
14	1	0	2.228976	-0.299388	2.168216
15	6	0	4.001232	-2.471184	0.240088
16	1	0	4.275687	-2.295002	-1.885794
17	1	0	3.581806	-2.392747	2.347373
18	1	0	4.591620	-3.376394	0.315898
19	7	0	-0.056850	0.836069	-0.474174
20	1	0	-2.380315	1.148906	-1.370841
21	1	0	-3.766488	-0.002081	-0.957153

22	6	0	-2.213283	-2.098550	-0.194497
23	8	0	-1.449566	-2.999656	0.052058
24	8	0	-3.552762	-2.257780	-0.177488
25	6	0	-3.995785	-3.577722	0.136295
26	1	0	-3.644893	-3.872041	1.125769
27	1	0	-5.081634	-3.539381	0.109517
28	1	0	-3.617491	-4.289997	-0.597408
29	6	0	-3.091986	1.493478	0.889233
30	6	0	-1.990056	2.300786	0.842508
31	1	0	-4.013699	1.866374	0.459963
32	1	0	-3.158865	0.745470	1.665611
33	1	0	-1.099837	2.132297	1.443195
34	8	0	-1.961884	3.318988	0.006451
35	6	0	-0.710028	4.000635	-0.164195
36	1	0	-0.161184	3.539341	-0.985140
37	1	0	-0.954383	5.033532	-0.400238
38	1	0	-0.115799	3.945011	0.748697

TS11′ (NIMAG=1; 295.6*i* cm^{-1})

Center Number	Atomic Number	Atomic Type	Coordinates (Angstroms) X	Y	Z
1	6	0	-2.147975	1.111375	-1.374199
2	6	0	-2.265502	-0.072166	-0.666959
3	6	0	-1.169601	-0.552007	0.097348
4	1	0	-1.299032	-1.451761	0.700321
5	16	0	1.086045	-0.409322	1.234671
6	8	0	0.679911	-1.650816	1.872266
7	8	0	1.437291	0.744145	2.051049
8	6	0	2.467545	-0.759439	0.164386
9	6	0	3.567792	0.086040	0.174868
10	6	0	2.414630	-1.888957	-0.644893
11	6	0	4.642457	-0.204691	-0.660442
12	1	0	3.578735	0.938243	0.843711
13	6	0	3.490280	-2.165247	-1.476748
14	1	0	1.550057	-2.541597	-0.609918
15	6	0	4.600070	-1.322589	-1.485474
16	1	0	5.513203	0.439373	-0.661173
17	1	0	3.467366	-3.040582	-2.113973
18	1	0	5.438559	-1.545047	-2.134449
19	7	0	-0.060891	0.131824	0.143968
20	1	0	-1.174558	1.425313	-1.731058
21	1	0	-3.018393	1.447691	-1.925597
22	6	0	-3.604727	-0.674381	-0.540119
23	8	0	-4.617974	-0.197635	-0.986540
24	8	0	-3.582604	-1.838191	0.136766
25	6	0	-4.852328	-2.471545	0.292709
26	1	0	-5.283836	-2.700478	-0.682007
27	1	0	-4.660538	-3.382652	0.852911
28	1	0	-5.536513	-1.820535	0.837469
29	6	0	-2.166877	2.782842	-0.011251
30	6	0	-0.851615	2.774803	0.349560
31	1	0	-2.473326	3.442162	-0.813786
32	1	0	-2.912527	2.474403	0.706786
33	1	0	-0.482390	2.343270	1.275905
34	8	0	0.050837	3.275458	-0.477066
35	6	0	1.427193	3.027294	-0.175622
36	1	0	1.579884	2.955995	0.901768
37	1	0	1.727226	2.090484	-0.646718
38	1	0	1.987859	3.860398	-0.593482

TS12′ (NIMAG=1; 283.2*i* cm^{-1})

Center Number	Atomic Number	Atomic Type	Coordinates (Angstroms) X	Y	Z
1	6	0	2.759971	0.761601	-0.868652
2	6	0	1.544530	1.353166	-0.579792
3	6	0	0.387898	0.541674	-0.404080
4	1	0	-0.551701	1.038056	-0.155996
5	16	0	-0.778479	-1.747519	-0.087225
6	8	0	-0.480218	-2.272532	1.239126
7	8	0	-0.944929	-2.662053	-1.200006
8	6	0	-2.258055	-0.760058	0.036416
9	6	0	-2.974171	-0.483168	-1.122685
10	6	0	-2.646674	-0.273522	1.278420
11	6	0	-4.112358	0.307857	-1.029169
12	1	0	-2.648685	-0.901405	-2.067745
13	6	0	-3.788822	0.515472	1.357669
14	1	0	-2.069923	-0.532558	2.158121
15	6	0	-4.515064	0.806602	0.206991
16	1	0	-4.688699	0.529324	-1.918879
17	1	0	-4.113874	0.897383	2.317546
18	1	0	-5.405296	1.420472	0.274255
19	7	0	0.493887	-0.748752	-0.473816
20	1	0	2.777173	-0.193197	-1.380322
21	1	0	3.616353	1.415096	-0.986626
22	6	0	1.525853	2.787378	-0.250926
23	8	0	2.499058	3.495618	-0.190147
24	8	0	0.277538	3.245619	-0.019220
25	6	0	0.194394	4.636081	0.291133
26	1	0	0.586440	5.232904	-0.532739
27	1	0	-0.861526	4.841672	0.445975
28	1	0	0.767197	4.859546	1.191551
29	6	0	3.577404	-0.208185	0.894050
30	6	0	2.853269	-1.363453	0.860329
31	1	0	4.565756	-0.208269	0.451611
32	1	0	3.357917	0.529803	1.651555
33	1	0	1.961827	-1.526722	1.460793
34	8	0	3.202723	-2.334150	0.037396
35	6	0	2.292054	-3.432441	-0.113599
36	1	0	1.613023	-3.224179	-0.940440
37	1	0	2.900478	-4.307063	-0.331229
38	1	0	1.717237	-3.583334	0.801056

TS13′ (NIMAG=1; 403.6*i* cm^{-1})

Center Number	Atomic Number	Atomic Type	Coordinates (Angstroms) X	Y	Z
1	6	0	-2.083013	0.941085	-1.044374
2	6	0	-2.203067	-0.358253	-0.548533
3	6	0	-1.074224	-0.950636	0.064281
4	1	0	-1.159079	-1.954049	0.485583
5	16	0	1.231335	-0.867964	1.134536
6	8	0	1.049210	-2.287055	1.391518
7	8	0	1.385367	0.056272	2.248325
8	6	0	2.629211	-0.665278	0.053175
9	6	0	3.096543	0.622300	-0.187646
10	6	0	3.216509	-1.785294	-0.515509
11	6	0	4.186845	0.784663	-1.030870
12	1	0	2.612048	1.465192	0.292600

13	6	0	4.310198	-1.607032	-1.357591
14	1	0	2.823926	-2.768649	-0.287053
15	6	0	4.790104	-0.327964	-1.614846
16	1	0	4.573670	1.776996	-1.228903
17	1	0	4.788294	-2.468239	-1.807849
18	1	0	5.643179	-0.195108	-2.269575
19	7	0	0.006862	-0.233128	0.193838
20	1	0	-1.115593	1.210073	-1.449938
21	1	0	-2.949829	1.384500	-1.518439
22	6	0	-3.491819	-1.049443	-0.423456
23	8	0	-3.638915	-2.183447	-0.044136
24	8	0	-4.531127	-0.263781	-0.787720
25	6	0	-5.810254	-0.891896	-0.706417
26	1	0	-6.014176	-1.209930	0.316343
27	1	0	-6.529520	-0.142304	-1.026336
28	1	0	-5.847447	-1.764367	-1.359267
29	6	0	-1.964309	2.268259	0.499645
30	6	0	-0.693363	2.149112	1.029141
31	1	0	-2.206875	3.137612	-0.099183
32	1	0	-2.772091	1.873784	1.099690
33	1	0	-0.455948	1.523901	1.880498
34	8	0	0.382966	2.778028	0.595761
35	6	0	0.251755	3.706510	-0.474777
36	1	0	-0.155153	3.224723	-1.364753
37	1	0	-0.387602	4.541319	-0.176703
38	1	0	1.256096	4.066936	-0.679492

TS14′ (NIMAG=1; 382.4*i* cm^{-1})

Center Number	Atomic Number	Atomic Type	Coordinates (Angstroms) X	Y	Z
1	6	0	2.608601	0.074939	-0.905457
2	6	0	1.604432	0.979723	-0.563175
3	6	0	0.289154	0.486807	-0.367915
4	1	0	-0.485314	1.201082	-0.078861
5	16	0	-1.364035	-1.481003	-0.011522
6	8	0	-1.176274	-1.958441	1.350764
7	8	0	-1.735195	-2.400534	-1.065820
8	6	0	-2.589803	-0.184633	0.035359
9	6	0	-3.207701	0.192503	-1.151793
10	6	0	-2.879243	0.434857	1.244468
11	6	0	-4.139565	1.221987	-1.121405
12	1	0	-2.969510	-0.330689	-2.070228
13	6	0	-3.815405	1.463056	1.261435
14	1	0	-2.387608	0.095820	2.148214
15	6	0	-4.439090	1.855987	0.081747
16	1	0	-4.635887	1.526559	-2.034551
17	1	0	-4.058169	1.954959	2.195209
18	1	0	-5.166795	2.658453	0.099722
19	7	0	0.083975	-0.792081	-0.458150
20	1	0	2.307388	-0.771459	-1.509710
21	1	0	3.607656	0.459864	-1.067164
22	6	0	1.873705	2.366159	-0.172584
23	8	0	1.034017	3.188638	0.099741
24	8	0	3.196989	2.644019	-0.143318
25	6	0	3.517649	3.989006	0.209584
26	1	0	3.139022	4.222728	1.205023
27	1	0	4.602663	4.050602	0.187314
28	1	0	3.078167	4.684961	-0.505496
29	6	0	3.113585	-1.084437	0.713208
30	6	0	2.048810	-1.932791	0.947889

31	1	0	4.024249	-1.484207	0.284172
32	1	0	3.239707	-0.267339	1.409268
33	1	0	1.235774	-1.686255	1.620407
34	8	0	1.869627	-3.122048	0.420301
35	6	0	2.842850	-3.626988	-0.485669
36	1	0	3.802362	-3.759580	0.021189
37	1	0	2.462359	-4.587029	-0.821098
38	1	0	2.959494	-2.957969	-1.339500

TS15′ (NIMAG=1; 387.7*i* cm^{-1})

Center Number	Atomic Number	Atomic Type	Coordinates (Angstroms) X	Y	Z
1	6	0	-1.984042	1.263507	-1.161596
2	6	0	-2.244605	-0.024927	-0.697187
3	6	0	-1.203232	-0.738381	-0.056980
4	1	0	-1.388662	-1.738039	0.337498
5	16	0	1.052517	-0.901353	1.100860
6	8	0	0.729251	-2.304702	1.305390
7	8	0	1.238262	-0.028150	2.250293
8	6	0	2.511486	-0.799914	0.088725
9	6	0	3.086814	0.449794	-0.115816
10	6	0	3.039513	-1.957995	-0.461323
11	6	0	4.226817	0.533362	-0.902758
12	1	0	2.643799	1.324977	0.346573
13	6	0	4.184457	-1.858767	-1.246473
14	1	0	2.561609	-2.909577	-0.263016
15	6	0	4.772402	-0.618612	-1.466989
16	1	0	4.696801	1.494539	-1.072399
17	1	0	4.617474	-2.751171	-1.681318
18	1	0	5.664549	-0.547225	-2.077739
19	7	0	-0.064318	-0.131007	0.129299
20	1	0	-0.983978	1.459865	-1.525819
21	1	0	-2.801958	1.786398	-1.644130
22	6	0	-3.640910	-0.478052	-0.647017
23	8	0	-4.594842	0.201590	-0.939595
24	8	0	-3.753765	-1.753679	-0.231622
25	6	0	-5.089189	-2.252237	-0.163869
26	1	0	-5.558089	-2.215965	-1.147541
27	1	0	-5.003523	-3.278152	0.183892
28	1	0	-5.682007	-1.657538	0.531667
29	6	0	-1.818746	2.553966	0.424365
30	6	0	-0.585639	2.308680	0.992579
31	1	0	-1.962861	3.448717	-0.168482
32	1	0	-2.683484	2.213594	0.976308
33	1	0	-0.435285	1.639659	1.830451
34	8	0	0.558956	2.847292	0.612798
35	6	0	0.547161	3.816490	-0.429298
36	1	0	0.130391	3.400714	-1.347701
37	1	0	-0.027099	4.694773	-0.123899
38	1	0	1.585678	4.092825	-0.589492

TS16′ (NIMAG=1; 363.5*i* cm^{-1})

Center Number	Atomic Number	Atomic Type	Coordinates (Angstroms) X	Y	Z
1	6	0	2.605990	0.812481	-0.933460
2	6	0	1.382788	1.393721	-0.609404
3	6	0	0.259966	0.549635	-0.405844

4	1	0	-0.694247	1.006169	-0.137062
5	16	0	-0.759496	-1.804039	-0.017479
6	8	0	-0.446218	-2.198344	1.347983
7	8	0	-0.856703	-2.798125	-1.064674
8	6	0	-2.305131	-0.909996	0.023867
9	6	0	-3.001828	-0.725036	-1.165181
10	6	0	-2.765268	-0.401939	1.232024
11	6	0	-4.190773	-0.007269	-1.138131
12	1	0	-2.620679	-1.157341	-2.082771
13	6	0	-3.958446	0.312610	1.246184
14	1	0	-2.200617	-0.588599	2.137492
15	6	0	-4.664906	0.511301	0.064286
16	1	0	-4.751315	0.141463	-2.052833
17	1	0	-4.337980	0.708779	2.180037
18	1	0	-5.594591	1.067535	0.080271
19	7	0	0.431942	-0.735838	-0.469267
20	1	0	2.579637	-0.100756	-1.513372
21	1	0	3.444624	1.481026	-1.090074
22	6	0	1.361362	2.811763	-0.236234
23	8	0	2.337332	3.516205	-0.143376
24	8	0	0.112897	3.264687	0.003509
25	6	0	0.030666	4.642501	0.364565
26	1	0	0.428694	5.269056	-0.434047
27	1	0	-1.025804	4.844540	0.520404
28	1	0	0.598027	4.831917	1.276273
29	6	0	3.429427	-0.096581	0.731716
30	6	0	2.667192	-1.221198	0.967162
31	1	0	4.423623	-0.202328	0.315657
32	1	0	3.283494	0.740872	1.399199
33	1	0	1.799513	-1.220749	1.616183
34	8	0	2.864681	-2.416885	0.458202
35	6	0	3.975371	-2.619365	-0.406849
36	1	0	4.912104	-2.449842	0.130599
37	1	0	3.912351	-3.654115	-0.729867
38	1	0	3.917139	-1.958215	-1.272914

TS17′ (NIMAG=1; 507.1*i* cm^{-1})

Center Number	Atomic Number	Atomic Type	Coordinates (Angstroms) X	Y	Z
1	6	0	1.786310	-0.075869	-1.601056
2	6	0	1.602163	0.669046	-0.441881
3	6	0	0.474712	0.396339	0.347938
4	1	0	0.382544	0.844816	1.336551
5	16	0	-1.668465	-0.872758	0.930281
6	8	0	-1.464277	-0.327860	2.261084
7	8	0	-1.943507	-2.283826	0.741864
8	6	0	-2.963633	0.062768	0.143917
9	6	0	-3.628067	-0.494626	-0.941368
10	6	0	-3.266071	1.329128	0.626514
11	6	0	-4.626184	0.245940	-1.561536
12	1	0	-3.375375	-1.495529	-1.269272
13	6	0	-4.266529	2.059312	-0.004624
14	1	0	-2.737251	1.716675	1.488960
15	6	0	-4.940707	1.519759	-1.095623
16	1	0	-5.162286	-0.172371	-2.404504
17	1	0	-4.523058	3.046379	0.359700
18	1	0	-5.721274	2.092427	-1.582085
19	7	0	-0.363921	-0.520089	-0.085136
20	1	0	0.905181	-0.386576	-2.147235
21	1	0	2.690879	0.074377	-2.178283

22	6	0	2.640766	1.541724	0.143272
23	8	0	2.528586	2.152193	1.173199
24	8	0	3.748581	1.609979	-0.625938
25	6	0	4.783254	2.448745	-0.110027
26	1	0	5.117368	2.084408	0.862090
27	1	0	5.590162	2.405400	-0.836967
28	1	0	4.420896	3.470480	0.002728
29	6	0	2.071175	-2.006900	-0.797061
30	6	0	0.780815	-2.385186	-0.499060
31	1	0	2.514346	-2.270404	-1.754871
32	1	0	0.529336	-2.683757	0.508599
33	1	0	0.103715	-2.675155	-1.288601
34	8	0	2.920269	-1.862027	0.259499
35	6	0	4.271974	-1.642282	-0.099565
36	1	0	4.379340	-0.717419	-0.674050
37	1	0	4.830715	-1.556676	0.830014
38	1	0	4.660564	-2.487384	-0.678008

TS18′ (NIMAG=1; 495.5*i* cm^{-1})

Center Number	Atomic Number	Atomic Type	Coordinates (Angstroms) X	Y	Z
1	6	0	2.310415	-1.479389	-0.712306
2	6	0	1.760554	-0.226245	-0.933387
3	6	0	0.360377	-0.116088	-0.996224
4	1	0	-0.084451	0.878462	-1.035529
5	16	0	-2.018300	-1.142094	-0.943674
6	8	0	-2.485772	-2.308466	-0.223451
7	8	0	-2.358662	-0.923377	-2.333503
8	6	0	-2.527475	0.301175	-0.026227
9	6	0	-2.664167	1.513684	-0.692803
10	6	0	-2.769894	0.182267	1.337739
11	6	0	-3.032685	2.639367	0.034911
12	1	0	-2.505608	1.557474	-1.763840
13	6	0	-3.141112	1.314251	2.052326
14	1	0	-2.703505	-0.788976	1.813440
15	6	0	-3.264513	2.539368	1.403007
16	1	0	-3.144313	3.591705	-0.468299
17	1	0	-3.342873	1.238496	3.113648
18	1	0	-3.553820	3.419122	1.965193
19	7	0	-0.337431	-1.216056	-0.848927
20	1	0	1.785638	-2.340194	-1.105543
21	1	0	3.383019	-1.568014	-0.588586
22	6	0	2.530327	1.033198	-0.855345
23	8	0	2.060106	2.134968	-0.967808
24	8	0	3.848238	0.820212	-0.664428
25	6	0	4.642705	2.005445	-0.593805
26	1	0	5.665817	1.668037	-0.450331
27	1	0	4.551544	2.576798	-1.517428
28	1	0	4.319530	2.629248	0.240363
29	6	0	1.582984	-1.878609	1.269123
30	6	0	0.275452	-2.221807	1.016665
31	1	0	2.360312	-2.636697	1.321992
32	1	0	-0.508150	-1.656701	1.501305
33	1	0	0.020773	-3.191158	0.615894
34	8	0	1.785546	-0.730144	1.976270
35	6	0	3.134890	-0.489430	2.330836
36	1	0	3.759487	-0.375858	1.439799
37	1	0	3.146796	0.437495	2.900464
38	1	0	3.521820	-1.301999	2.955015

TS19′ (NIMAG=1; 507.1*i* cm^{-1})

Center Number	Atomic Number	Atomic Type	Coordinates (Angstroms) X	Y	Z
1	6	0	1.786127	-0.075835	-1.601109
2	6	0	1.602048	0.669176	-0.441981
3	6	0	0.474623	0.396558	0.347899
4	1	0	0.382479	0.845124	1.336472
5	16	0	-1.668500	-0.872572	0.930414
6	8	0	-1.464336	-0.327469	2.261136
7	8	0	-1.943436	-2.283688	0.742198
8	6	0	-2.963748	0.062742	0.143931
9	6	0	-3.628276	-0.494933	-0.941151
10	6	0	-3.266137	1.329232	0.626219
11	6	0	-4.626444	0.245476	-1.561428
12	1	0	-3.375619	-1.495925	-1.268808
13	6	0	-4.266644	2.059256	-0.005024
14	1	0	-2.737247	1.717001	1.488524
15	6	0	-4.940920	1.519418	-1.095824
16	1	0	-5.162622	-0.173055	-2.404238
17	1	0	-4.523138	3.046419	0.359063
18	1	0	-5.721526	2.091962	-1.582368
19	7	0	-0.363992	-0.519949	-0.085062
20	1	0	0.904966	-0.386575	-2.147220
21	1	0	2.690662	0.074377	-2.178396
22	6	0	2.640837	1.541671	0.143197
23	8	0	2.528725	2.152232	1.173061
24	8	0	3.748752	1.609543	-0.625909
25	6	0	4.783642	2.447990	-0.109919
26	1	0	5.590785	2.404013	-0.836561
27	1	0	4.421740	3.469942	0.002351
28	1	0	5.117239	2.083829	0.862440
29	6	0	2.071182	-2.006681	-0.796947
30	6	0	0.780903	-2.385025	-0.498628
31	1	0	2.514186	-2.270393	-1.754776
32	1	0	0.529633	-2.683355	0.509155
33	1	0	0.103695	-2.675268	-1.287976
34	8	0	2.920471	-1.861473	0.259421
35	6	0	4.272162	-1.642199	-0.099988
36	1	0	4.660601	-2.487908	-0.677643
37	1	0	4.379572	-0.717910	-0.675394
38	1	0	4.831016	-1.555733	0.829444

TS20′ (NIMAG=1; 484.0*i* cm^{-1})

Center Number	Atomic Number	Atomic Type	Coordinates (Angstroms) X	Y	Z
1	6	0	2.422953	-1.429662	-0.891713
2	6	0	1.848476	-0.170059	-0.969224
3	6	0	0.445392	-0.077017	-0.997805
4	1	0	-0.029162	0.900761	-0.926207
5	16	0	-1.909224	-1.157407	-1.003090
6	8	0	-2.337295	-2.388984	-0.372705
7	8	0	-2.290793	-0.829218	-2.360576
8	6	0	-2.427524	0.193036	0.043786
9	6	0	-2.634426	1.446587	-0.520791
10	6	0	-2.608966	-0.037905	1.402782
11	6	0	-3.013992	2.498565	0.305355
12	1	0	-2.522582	1.575948	-1.590875

13	6	0	-2.989943	1.021287	2.216960
14	1	0	-2.489300	-1.039093	1.799383
15	6	0	-3.185036	2.286331	1.669676
16	1	0	-3.184913	3.480485	-0.118391
17	1	0	-3.144797	0.857208	3.276173
18	1	0	-3.484447	3.108284	2.308832
19	7	0	-0.228170	-1.201361	-0.952028
20	1	0	1.915478	-2.256712	-1.369539
21	1	0	3.500964	-1.491462	-0.792615
22	6	0	2.688896	1.021742	-0.730030
23	8	0	3.868183	0.991254	-0.478618
24	8	0	1.997823	2.167636	-0.839641
25	6	0	2.761021	3.358102	-0.640062
26	1	0	2.064102	4.180858	-0.773694
27	1	0	3.185504	3.371237	0.364059
28	1	0	3.570317	3.414928	-1.368105
29	6	0	1.773734	-2.050819	1.048682
30	6	0	0.464067	-2.386993	0.799854
31	1	0	2.560200	-2.799347	0.988905
32	1	0	-0.314551	-1.885048	1.356469
33	1	0	0.210019	-3.309819	0.300975
34	8	0	1.991619	-0.989045	1.873823
35	6	0	3.353329	-0.781438	2.216692
36	1	0	3.768663	-1.675434	2.693747
37	1	0	3.943155	-0.512483	1.336373
38	1	0	3.371930	0.044033	2.924921

TS21′ (NIMAG=1; 537.4*i* cm^{-1})

Center Number	Atomic Number	Atomic Type	Coordinates (Angstroms) X	Y	Z
1	6	0	-1.835181	0.073743	1.752639
2	6	0	-1.659892	0.750006	0.541432
3	6	0	-0.530300	0.445172	-0.223591
4	1	0	-0.447236	0.827345	-1.241206
5	16	0	1.562852	-0.913928	-0.770507
6	8	0	1.286835	-0.517660	-2.142169
7	8	0	1.836273	-2.301817	-0.448462
8	6	0	2.907416	0.082491	-0.165195
9	6	0	3.606580	-0.348493	0.955552
10	6	0	3.211652	1.270355	-0.816703
11	6	0	4.642361	0.441560	1.436874
12	1	0	3.349933	-1.293407	1.418600
13	6	0	4.250750	2.051134	-0.323291
14	1	0	2.654143	1.557566	-1.700036
15	6	0	4.959866	1.638636	0.800362
16	1	0	5.205377	0.121399	2.304875
17	1	0	4.509478	2.977946	-0.820162
18	1	0	5.769942	2.250302	1.179149
19	7	0	0.309674	-0.450830	0.257349
20	1	0	-0.950183	-0.139525	2.339905
21	1	0	-2.753669	0.251829	2.298121
22	6	0	-2.754314	1.488590	-0.123792
23	8	0	-2.709706	1.913138	-1.250452
24	8	0	-3.821642	1.646691	0.676336
25	6	0	-4.928876	2.317619	0.075044
26	1	0	-5.280125	1.762267	-0.795122
27	1	0	-5.699548	2.360158	0.839892
28	1	0	-4.639487	3.320683	-0.238860
29	6	0	-2.161168	-1.823686	1.160702
30	6	0	-0.888952	-2.266100	0.839334

31	1	0	-2.531358	-2.028124	2.159582
32	1	0	-0.624894	-2.666294	-0.128178
33	1	0	-0.202414	-2.485159	1.644114
34	8	0	-3.189429	-1.730870	0.262838
35	6	0	-2.872744	-1.963457	-1.100396
36	1	0	-2.609209	-3.013285	-1.266301
37	1	0	-3.771789	-1.721840	-1.662605
38	1	0	-2.054140	-1.324494	-1.443648

TS22′ (NIMAG=1; 525.2*i* cm^{-1})

Center Number	Atomic Number	Atomic Type	Coordinates (Angstroms) X	Y	Z
1	6	0	2.294976	-1.698393	0.523350
2	6	0	1.931853	-0.821749	-0.497167
3	6	0	0.593408	-0.798757	-0.906334
4	1	0	0.283432	-0.031704	-1.615625
5	16	0	-1.881304	-1.453312	-0.699607
6	8	0	-2.583904	-2.281389	0.256815
7	8	0	-2.008974	-1.648732	-2.129208
8	6	0	-2.306405	0.248248	-0.346547
9	6	0	-2.071445	1.224089	-1.309894
10	6	0	-2.837097	0.566295	0.899345
11	6	0	-2.325339	2.553940	-0.993744
12	1	0	-1.721508	0.938266	-2.294632
13	6	0	-3.095231	1.898731	1.200304
14	1	0	-3.065561	-0.228372	1.600378
15	6	0	-2.824685	2.890217	0.261140
16	1	0	-2.142299	3.325054	-1.731660
17	1	0	-3.516959	2.161310	2.162898
18	1	0	-3.023581	3.927884	0.500784
19	7	0	-0.251329	-1.593445	-0.286721
20	1	0	1.797984	-2.659967	0.562851
21	1	0	3.310417	-1.649476	0.896271
22	6	0	2.787340	0.306426	-0.925482
23	8	0	2.423353	1.208591	-1.637647
24	8	0	4.032076	0.217946	-0.433611
25	6	0	4.896052	1.300253	-0.781691
26	1	0	4.490863	2.241620	-0.409437
27	1	0	5.849999	1.079698	-0.310682
28	1	0	5.004790	1.364405	-1.864398
29	6	0	1.301966	-0.915909	2.141718
30	6	0	0.010923	-1.355700	1.920070
31	1	0	1.959194	-1.518220	2.758148
32	1	0	-0.822626	-0.671746	1.875336
33	1	0	-0.225686	-2.396804	2.085058
34	8	0	1.666982	0.401802	2.163870
35	6	0	0.671209	1.351901	1.819047
36	1	0	-0.122469	1.380341	2.573984
37	1	0	1.175067	2.315150	1.785543
38	1	0	0.227706	1.146375	0.839499

TS23′ (NIMAG=1; 530.3*i* cm^{-1})

Center Number	Atomic Number	Atomic Type	Coordinates (Angstroms) X	Y	Z
1	6	0	-1.777406	-0.381064	1.931864
2	6	0	-1.695926	0.556431	0.897326
3	6	0	-0.625677	0.463089	0.002619

4	1	0	-0.603211	1.072317	-0.899597
5	16	0	1.430459	-0.682105	-0.988413
6	8	0	1.094096	0.081849	-2.179964
7	8	0	1.681738	-2.108253	-1.079938
8	6	0	2.836006	0.089132	-0.215732
9	6	0	3.574337	-0.640322	0.708176
10	6	0	3.148493	1.401941	-0.542166
11	6	0	4.658501	-0.028967	1.324102
12	1	0	3.309333	-1.669827	0.915759
13	6	0	4.236521	2.001173	0.082361
14	1	0	2.558578	1.926269	-1.284100
15	6	0	4.985169	1.288568	1.013534
16	1	0	5.251876	-0.581346	2.042207
17	1	0	4.502276	3.021993	-0.162853
18	1	0	5.833039	1.759982	1.495916
19	7	0	0.251875	-0.502656	0.201180
20	1	0	-0.855754	-0.702713	2.400514
21	1	0	-2.663146	-0.335966	2.555932
22	6	0	-2.886470	1.382793	0.603318
23	8	0	-3.867717	1.440916	1.296203
24	8	0	-2.753426	2.081497	-0.540220
25	6	0	-3.859836	2.924274	-0.862908
26	1	0	-4.021407	3.657463	-0.072265
27	1	0	-3.595014	3.415240	-1.795414
28	1	0	-4.765711	2.329456	-0.982769
29	6	0	-2.137901	-2.107171	0.964565
30	6	0	-0.891138	-2.436670	0.461378
31	1	0	-2.427719	-2.530015	1.920578
32	1	0	-0.694574	-2.605961	-0.586696
33	1	0	-0.141191	-2.801776	1.147895
34	8	0	-3.234033	-1.852205	0.188553
35	6	0	-3.021488	-1.759461	-1.209388
36	1	0	-2.245033	-1.028908	-1.455263
37	1	0	-2.749160	-2.733244	-1.630154
38	1	0	-3.968015	-1.433601	-1.634712

TS24′ (NIMAG=1; 516.3*i* cm^{-1})

Center Number	Atomic Number	Atomic Type	Coordinates (Angstroms) X	Y	Z
1	6	0	2.313866	-1.917692	0.242435
2	6	0	2.040848	-0.784632	-0.521871
3	6	0	0.737693	-0.601120	-0.999761
4	1	0	0.483160	0.327906	-1.506825
5	16	0	-1.752965	-1.192780	-1.144076
6	8	0	-2.539133	-2.245966	-0.539138
7	8	0	-1.771998	-0.936965	-2.570419
8	6	0	-2.187464	0.336315	-0.321244
9	6	0	-1.876593	1.549191	-0.927787
10	6	0	-2.805588	0.281886	0.923507
11	6	0	-2.143978	2.729895	-0.244040
12	1	0	-1.461695	1.560599	-1.928626
13	6	0	-3.074522	1.469560	1.594371
14	1	0	-3.091018	-0.679374	1.335433
15	6	0	-2.729738	2.688929	1.017988
16	1	0	-1.907336	3.681917	-0.702968
17	1	0	-3.562967	1.443239	2.560849
18	1	0	-2.939117	3.612205	1.544727
19	7	0	-0.163707	-1.503870	-0.676138
20	1	0	1.808351	-2.840671	-0.011530
21	1	0	3.306185	-1.989006	0.673464

22	6	0	3.033475	0.312467	-0.536362
23	8	0	4.141277	0.240009	-0.076101
24	8	0	2.558567	1.423490	-1.133894
25	6	0	3.489355	2.503441	-1.218488
26	1	0	4.372251	2.199505	-1.781157
27	1	0	2.965465	3.306305	-1.730242
28	1	0	3.796977	2.816234	-0.220396
29	6	0	1.224004	-1.585403	1.950318
30	6	0	-0.051647	-1.908784	1.531301
31	1	0	1.835218	-2.354955	2.407547
32	1	0	-0.874233	-1.216936	1.626256
33	1	0	-0.306269	-2.946351	1.371132
34	8	0	1.596920	-0.340485	2.370683
35	6	0	0.641121	0.697533	2.236310
36	1	0	0.272880	0.786882	1.208864
37	1	0	-0.209858	0.539056	2.908451
38	1	0	1.155340	1.614847	2.514088

TS25′ (NIMAG=1; 531.3i cm^{-1})

Center Number	Atomic Number	Atomic Type	Coordinates (Angstroms) X	Y	Z
1	6	0	-2.033319	0.462663	-0.960048
2	6	0	-1.729635	-0.750181	-0.343656
3	6	0	-0.507631	-0.850580	0.334964
4	1	0	-0.276333	-1.741361	0.919052
5	16	0	1.645565	0.123955	1.311406
6	8	0	1.616312	-1.046937	2.172239
7	8	0	1.840838	1.446484	1.873103
8	6	0	2.877100	-0.144482	0.054523
9	6	0	3.330958	0.945211	-0.678887
10	6	0	3.340508	-1.433702	-0.170269
11	6	0	4.279597	0.730008	-1.669799
12	1	0	2.955811	1.936866	-0.457348
13	6	0	4.290770	-1.633860	-1.165423
14	1	0	2.972213	-2.250769	0.438185
15	6	0	4.754997	-0.556275	-1.912681
16	1	0	4.651712	1.565723	-2.249473
17	1	0	4.671400	-2.630299	-1.352703
18	1	0	5.496644	-0.717901	-2.685816
19	7	0	0.245467	0.228942	0.375304
20	1	0	-1.211520	1.022928	-1.389212
21	1	0	-3.000142	0.561983	-1.438211
22	6	0	-2.706239	-1.844837	-0.171128
23	8	0	-2.464658	-2.909497	0.334168
24	8	0	-3.922072	-1.523923	-0.656324
25	6	0	-4.905610	-2.552686	-0.534031
26	1	0	-5.816382	-2.144045	-0.963577
27	1	0	-4.589851	-3.444220	-1.076168
28	1	0	-5.055245	-2.812075	0.514250
29	6	0	-2.158815	2.031710	0.370239
30	6	0	-1.101322	1.816924	1.229190
31	1	0	-3.164044	1.739163	0.663812
32	1	0	-1.237089	1.166871	2.081230
33	1	0	-0.256982	2.491991	1.241159
34	8	0	-2.066620	3.098241	-0.489647
35	6	0	-3.296419	3.497878	-1.061290
36	1	0	-4.005068	3.808720	-0.286543
37	1	0	-3.079894	4.344062	-1.709471
38	1	0	-3.742571	2.694800	-1.657319

TS26′ (NIMAG=1; 517.8*i* cm^{-1})

Center Number	Atomic Number	Atomic Type	Coordinates (Angstroms) X	Y	Z
1	6	0	2.508241	-0.406564	-0.605867
2	6	0	1.653563	0.684763	-0.710025
3	6	0	0.288369	0.435194	-0.917563
4	1	0	-0.405793	1.276530	-0.916350
5	16	0	-1.735012	-1.154843	-1.116193
6	8	0	-1.901305	-2.510555	-0.635726
7	8	0	-2.112251	-0.772006	-2.460439
8	6	0	-2.596337	-0.076447	0.017157
9	6	0	-3.063456	1.151911	-0.435702
10	6	0	-2.774990	-0.489611	1.333263
11	6	0	-3.706216	1.996193	0.462971
12	1	0	-2.941737	1.419889	-1.478607
13	6	0	-3.421035	0.362572	2.220296
14	1	0	-2.439791	-1.474811	1.636181
15	6	0	-3.878461	1.604111	1.786459
16	1	0	-4.077582	2.956275	0.126829
17	1	0	-3.578399	0.053466	3.246350
18	1	0	-4.382736	2.264349	2.481801
19	7	0	-0.093993	-0.820836	-0.928153
20	1	0	2.272575	-1.287321	-1.190844
21	1	0	3.551274	-0.219197	-0.382699
22	6	0	2.055036	2.072646	-0.397058
23	8	0	1.329602	3.030166	-0.473254
24	8	0	3.343559	2.159072	-0.018448
25	6	0	3.795141	3.482356	0.275473
26	1	0	4.839139	3.382128	0.559726
27	1	0	3.694065	4.119706	-0.603067
28	1	0	3.211665	3.909995	1.091121
29	6	0	2.028817	-1.659574	1.014245
30	6	0	0.651945	-1.697445	0.975407
31	1	0	2.541302	-0.994435	1.704545
32	1	0	0.096710	-0.932541	1.500181
33	1	0	0.141956	-2.613843	0.707807
34	8	0	2.703752	-2.794953	0.652377
35	6	0	4.074497	-2.803695	0.998611
36	1	0	4.205009	-2.696876	2.080625
37	1	0	4.469820	-3.766365	0.682797
38	1	0	4.623126	-2.005854	0.487681

TS27′ (NIMAG=1; 522.1*i* cm^{-1})

Center Number	Atomic Number	Atomic Type	Coordinates (Angstroms) X	Y	Z
1	6	0	-2.056016	0.690663	-1.047416
2	6	0	-1.820849	-0.588473	-0.545458
3	6	0	-0.633624	-0.807304	0.166497
4	1	0	-0.446381	-1.756164	0.666902
5	16	0	1.506178	-0.016412	1.319478
6	8	0	1.428307	-1.292824	2.012395
7	8	0	1.688690	1.214209	2.064591
8	6	0	2.793130	-0.131487	0.095061
9	6	0	3.269264	1.038947	-0.483823
10	6	0	3.278086	-1.382975	-0.259177
11	6	0	4.262271	0.946123	-1.449769
12	1	0	2.875933	1.996360	-0.164622

13	6	0	4.273507	-1.460514	-1.227117
14	1	0	2.890584	-2.267292	0.231829
15	6	0	4.760089	-0.300581	-1.820834
16	1	0	4.651500	1.845933	-1.909914
17	1	0	4.671652	-2.426280	-1.512902
18	1	0	5.536579	-0.366725	-2.573536
19	7	0	0.153357	0.234178	0.345794
20	1	0	-1.205211	1.263397	-1.394558
21	1	0	-3.006779	0.854779	-1.542320
22	6	0	-2.924994	-1.569920	-0.545862
23	8	0	-4.042334	-1.339296	-0.933633
24	8	0	-2.547439	-2.765919	-0.065177
25	6	0	-3.570578	-3.762244	-0.046729
26	1	0	-3.938148	-3.945664	-1.056582
27	1	0	-3.105226	-4.654778	0.362220
28	1	0	-4.402440	-3.439084	0.579490
29	6	0	-2.204374	2.118486	0.420882
30	6	0	-1.194876	1.784967	1.298846
31	1	0	-3.229455	1.825957	0.636979
32	1	0	-1.385182	1.052031	2.069015
33	1	0	-0.332017	2.425539	1.416448
34	8	0	-2.045105	3.264271	-0.317442
35	6	0	-3.232208	3.744331	-0.919122
36	1	0	-3.986478	3.979558	-0.161189
37	1	0	-2.965439	4.653024	-1.453996
38	1	0	-3.645748	3.019123	-1.627216

TS28′ (NIMAG=1; 506.6*i* cm^{-1})

Center Number	Atomic Number	Atomic Type	Coordinates (Angstroms) X	Y	Z
1	6	0	2.667886	-0.073587	-0.558732
2	6	0	1.676570	0.897968	-0.633756
3	6	0	0.358021	0.481048	-0.874510
4	1	0	-0.450770	1.211167	-0.854689
5	16	0	-1.425008	-1.360574	-1.167542
6	8	0	-1.409535	-2.745727	-0.746792
7	8	0	-1.843819	-0.973285	-2.498567
8	6	0	-2.437395	-0.461133	-0.001609
9	6	0	-3.084285	0.697535	-0.415198
10	6	0	-2.554306	-0.941726	1.298386
11	6	0	-3.849442	1.403170	0.507173
12	1	0	-3.004276	1.014327	-1.448478
13	6	0	-3.322260	-0.227733	2.209977
14	1	0	-2.076162	-1.875778	1.569885
15	6	0	-3.961978	0.944541	1.815685
16	1	0	-4.365045	2.304889	0.200560
17	1	0	-3.432768	-0.592698	3.223719
18	1	0	-4.562648	1.495482	2.529420
19	7	0	0.150534	-0.814567	-0.941204
20	1	0	2.570433	-0.953042	-1.182724
21	1	0	3.666900	0.261316	-0.303390
22	6	0	1.993312	2.278826	-0.210116
23	8	0	3.059272	2.632604	0.223844
24	8	0	0.951758	3.113901	-0.367127
25	6	0	1.203684	4.470714	0.001787
26	1	0	1.473799	4.532518	1.056200
27	1	0	2.017522	4.880147	-0.596836
28	1	0	0.277766	5.005620	-0.190791
29	6	0	2.360011	-1.433097	1.008772
30	6	0	1.002482	-1.658498	0.950065

31	1	0	2.769277	-0.723752	1.724052
32	1	0	0.342417	-0.992411	1.487775
33	1	0	0.623906	-2.624262	0.640803
34	8	0	3.188886	-2.450597	0.620959
35	6	0	4.548800	-2.271638	0.968053
36	1	0	4.664143	-2.164731	2.051603
37	1	0	5.074262	-3.164410	0.637139
38	1	0	4.978546	-1.396721	0.470454

TS29′ (NIMAG=1; 548.3*i* cm^{-1})

Center Number	Atomic Number	Atomic Type	Coordinates (Angstroms) X	Y	Z
1	6	0	-2.107704	0.599996	-1.065938
2	6	0	-1.905710	-0.598427	-0.373923
3	6	0	-0.710878	-0.741702	0.336730
4	1	0	-0.559063	-1.601072	0.989778
5	16	0	1.483357	0.165068	1.300488
6	8	0	1.318849	-0.862595	2.314717
7	8	0	1.813258	1.528275	1.674771
8	6	0	2.697153	-0.403221	0.128315
9	6	0	3.325764	0.527824	-0.689294
10	6	0	2.970854	-1.762263	0.049124
11	6	0	4.256359	0.077401	-1.616728
12	1	0	3.100304	1.581182	-0.575523
13	6	0	3.903801	-2.198874	-0.884527
14	1	0	2.473650	-2.451586	0.720903
15	6	0	4.540492	-1.282168	-1.715155
16	1	0	4.763938	0.786644	-2.258822
17	1	0	4.137096	-3.253810	-0.958446
18	1	0	5.268205	-1.627976	-2.439487
19	7	0	0.114213	0.290499	0.328344
20	1	0	-1.232854	1.035189	-1.534534
21	1	0	-3.051900	0.763350	-1.569679
22	6	0	-2.962782	-1.608672	-0.158926
23	8	0	-2.804840	-2.657395	0.409225
24	8	0	-4.142663	-1.228106	-0.682883
25	6	0	-5.203445	-2.171207	-0.520322
26	1	0	-5.386239	-2.357601	0.538167
27	1	0	-6.074178	-1.719991	-0.988279
28	1	0	-4.948945	-3.113538	-1.005803
29	6	0	-2.119525	2.232563	0.134493
30	6	0	-1.166241	1.951187	1.099524
31	1	0	-3.164484	2.069590	0.369819
32	1	0	-1.463307	1.320625	1.926618
33	1	0	-0.264993	2.531087	1.237034
34	8	0	-1.977646	3.220799	-0.803132
35	6	0	-0.655739	3.692436	-1.001520
36	1	0	0.049401	2.860049	-1.105901
37	1	0	-0.674426	4.280277	-1.916340
38	1	0	-0.335457	4.325940	-0.168418

TS30′ (NIMAG=1; 540.4*i* cm^{-1})

Center Number	Atomic Number	Atomic Type	Coordinates (Angstroms) X	Y	Z
1	6	0	-2.598583	0.639961	-0.360329
2	6	0	-1.869318	-0.526115	-0.595421
3	6	0	-0.500817	-0.395921	-0.856678

4	1	0	0.109507	-1.294729	-0.952554
5	16	0	1.649012	1.037379	-1.035631
6	8	0	1.944328	2.330238	-0.448574
7	8	0	1.928589	0.759729	-2.428770
8	6	0	2.474077	-0.204186	-0.054513
9	6	0	2.817237	-1.415278	-0.644109
10	6	0	2.740157	0.062122	1.284516
11	6	0	3.425673	-2.391192	0.137603
12	1	0	2.627699	-1.570155	-1.699730
13	6	0	3.350502	-0.920775	2.053548
14	1	0	2.498377	1.034459	1.697505
15	6	0	3.685715	-2.145413	1.481769
16	1	0	3.701181	-3.339995	-0.305652
17	1	0	3.574777	-0.727724	3.095407
18	1	0	4.162304	-2.908168	2.085694
19	7	0	0.002500	0.819677	-0.781559
20	1	0	-2.310378	1.512186	-0.934615
21	1	0	-3.642871	0.564632	-0.085530
22	6	0	-2.397775	-1.888005	-0.366136
23	8	0	-1.780531	-2.903447	-0.558753
24	8	0	-3.665720	-1.873570	0.080119
25	6	0	-4.240073	-3.162923	0.302132
26	1	0	-3.663937	-3.711420	1.047682
27	1	0	-5.251081	-2.978800	0.655038
28	1	0	-4.253053	-3.735094	-0.625628
29	6	0	-1.929555	1.711415	1.267183
30	6	0	-0.558984	1.518425	1.236811
31	1	0	-2.535177	1.099367	1.924193
32	1	0	-0.177816	0.601604	1.667568
33	1	0	0.144569	2.327821	1.098625
34	8	0	-2.531009	2.912137	1.018379
35	6	0	-1.725209	3.880331	0.362146
36	1	0	-1.197307	3.444932	-0.492770
37	1	0	-2.401653	4.661723	0.024305
38	1	0	-0.990382	4.308191	1.051142

TS31′ (NIMAG=1; 540.1*i* cm^{-1})

Center Number	Atomic Number	Atomic Type	Coordinates (Angstroms) X	Y	Z
1	6	0	-2.077730	0.888081	-1.184696
2	6	0	-1.991454	-0.374613	-0.589761
3	6	0	-0.852920	-0.660064	0.169238
4	1	0	-0.781508	-1.576437	0.753016
5	16	0	1.331608	0.013149	1.323740
6	8	0	1.067868	-1.121947	2.193288
7	8	0	1.688968	1.304243	1.882627
8	6	0	2.595847	-0.462607	0.163451
9	6	0	3.296900	0.531954	-0.507450
10	6	0	2.836661	-1.812529	-0.054424
11	6	0	4.267403	0.156843	-1.427354
12	1	0	3.094679	1.573170	-0.287531
13	6	0	3.810679	-2.173588	-0.978449
14	1	0	2.282394	-2.554941	0.506921
15	6	0	4.519685	-1.192058	-1.663554
16	1	0	4.830852	0.915836	-1.955920
17	1	0	4.019469	-3.220920	-1.158262
18	1	0	5.278896	-1.479194	-2.381142
19	7	0	0.033059	0.313227	0.297149
20	1	0	-1.157771	1.306546	-1.574272
21	1	0	-2.989332	1.139105	-1.714155

22	6	0	-3.191787	-1.236240	-0.552959
23	8	0	-4.271631	-0.919820	-0.981218
24	8	0	-2.946520	-2.429662	0.013732
25	6	0	-4.067943	-3.312273	0.074154
26	1	0	-3.704666	-4.218077	0.551660
27	1	0	-4.871784	-2.863603	0.658183
28	1	0	-4.436176	-3.525097	-0.929662
29	6	0	-2.073477	2.409892	0.146130
30	6	0	-1.202003	1.989371	1.136862
31	1	0	-3.139287	2.290656	0.302432
32	1	0	-1.585641	1.306835	1.882955
33	1	0	-0.278247	2.495086	1.378127
34	8	0	-1.816068	3.463273	-0.689914
35	6	0	-0.459617	3.865298	-0.770385
36	1	0	0.200736	3.001719	-0.909838
37	1	0	-0.387535	4.529858	-1.628250
38	1	0	-0.154635	4.402970	0.132915

TS32′ (NIMAG=1; 530.9*i* cm^{-1})

Center Number	Atomic Number	Atomic Type	Coordinates (Angstroms) X	Y	Z
1	6	0	2.799192	-0.178981	-0.284810
2	6	0	1.875572	0.847698	-0.483445
3	6	0	0.556832	0.490747	-0.788208
4	1	0	-0.212603	1.258881	-0.861301
5	16	0	-1.288908	-1.299651	-1.091071
6	8	0	-1.354433	-2.652260	-0.572468
7	8	0	-1.593629	-1.006488	-2.476087
8	6	0	-2.346567	-0.280528	-0.075533
9	6	0	-2.916633	0.861932	-0.624866
10	6	0	-2.566912	-0.646461	1.248158
11	6	0	-3.712916	1.667234	0.182407
12	1	0	-2.753480	1.090415	-1.671548
13	6	0	-3.364714	0.166443	2.043649
14	1	0	-2.143284	-1.568539	1.628698
15	6	0	-3.930446	1.322679	1.512416
16	1	0	-4.169921	2.557997	-0.230601
17	1	0	-3.555449	-0.108187	3.073807
18	1	0	-4.554565	1.951253	2.136170
19	7	0	0.282825	-0.799183	-0.783177
20	1	0	2.694616	-1.058973	-0.906992
21	1	0	3.802215	0.088037	0.026182
22	6	0	2.245770	2.232847	-0.120294
23	8	0	3.297660	2.552743	0.368495
24	8	0	1.269263	3.112951	-0.403424
25	6	0	1.576928	4.474173	-0.098320
26	1	0	2.454873	4.797622	-0.657713
27	1	0	0.701571	5.047393	-0.391161
28	1	0	1.774841	4.587967	0.967732
29	6	0	2.317678	-1.430630	1.275184
30	6	0	0.935892	-1.484472	1.223863
31	1	0	2.795831	-0.749330	1.968662
32	1	0	0.390996	-0.669127	1.681625
33	1	0	0.389923	-2.397939	1.032079
34	8	0	3.127763	-2.490510	0.985851
35	6	0	2.518516	-3.553974	0.268354
36	1	0	1.933604	-3.178735	-0.578020
37	1	0	3.329060	-4.184663	-0.088662
38	1	0	1.862370	-4.139101	0.920175

TS1″ (NIMAG=1; 363.3i cm^{-1})

Center Number	Atomic Number	Atomic Type	Coordinates (Angstroms) X	Y	Z
1	6	0	-1.925854	-1.357479	2.417177
2	6	0	-1.918639	-0.043126	1.973413
3	6	0	-1.019070	0.314274	0.952975
4	1	0	-2.652629	0.678956	2.305126
5	16	0	0.637676	-0.301382	-0.939907
6	8	0	0.325083	1.008638	-1.507133
7	8	0	0.520572	-1.476022	-1.793191
8	6	0	2.296580	-0.256569	-0.294946
9	6	0	3.108456	-1.370466	-0.459586
10	6	0	2.730687	0.891149	0.359472
11	6	0	4.402106	-1.330944	0.049009
12	1	0	2.727481	-2.234722	-0.989187
13	6	0	4.024156	0.911961	0.864864
14	1	0	2.066672	1.740974	0.469803
15	6	0	4.855819	-0.194393	0.709992
16	1	0	5.054977	-2.186324	-0.074382
17	1	0	4.384220	1.795178	1.377947
18	1	0	5.864664	-0.168297	1.104610
19	7	0	-0.291986	-0.626072	0.388257
20	1	0	-1.006238	-1.919611	2.317624
21	6	0	-2.877576	-2.626217	1.041417
22	6	0	-2.478415	-2.122762	-0.170294
23	1	0	-2.470584	-3.577160	1.355436
24	1	0	-3.870995	-2.363960	1.381579
25	1	0	-1.639196	-2.509065	-0.742344
26	8	0	-3.101810	-1.066166	-0.640319
27	6	0	-2.659010	-0.526110	-1.895853
28	1	0	-3.538429	-0.439344	-2.531834
29	1	0	-2.228251	0.456346	-1.710955
30	1	0	-1.915111	-1.178093	-2.354520
31	1	0	-2.568557	-1.630699	3.245843
32	6	0	-0.944372	1.791367	0.603203
33	8	0	-0.072042	2.511648	0.998175
34	8	0	-1.999552	2.190936	-0.103125
35	6	0	-1.927357	3.548596	-0.558359
36	1	0	-1.047331	3.665591	-1.191324
37	1	0	-2.839769	3.717892	-1.122977
38	1	0	-1.862911	4.228995	0.290112

TS2″ (NIMAG=1; 373.3i cm^{-1})

Center Number	Atomic Number	Atomic Type	Coordinates (Angstroms) X	Y	Z
1	6	0	-2.867020	-0.053430	2.171007
2	6	0	-1.963580	0.905815	1.731105
3	6	0	-0.928927	0.491815	0.872342
4	1	0	-2.086921	1.959822	1.936458
5	16	0	0.258727	-1.550564	-0.377571
6	8	0	0.039653	-1.382661	-1.809750
7	8	0	0.274325	-2.895336	0.161499
8	6	0	1.838431	-0.809871	0.028260
9	6	0	2.173171	-0.591387	1.359119
10	6	0	2.726730	-0.540606	-1.004507
11	6	0	3.424051	-0.062976	1.657162
12	1	0	1.465232	-0.824505	2.146796

13	6	0	3.983451	-0.034500	-0.692066
14	1	0	2.419799	-0.724163	-2.027439
15	6	0	4.327690	0.210015	0.634514
16	1	0	3.694069	0.128839	2.688107
17	1	0	4.691985	0.173776	-1.484861
18	1	0	5.304632	0.613111	0.872978
19	7	0	-0.915835	-0.759205	0.471705
20	1	0	-2.506795	-1.072997	2.234294
21	1	0	-3.635853	0.233891	2.878580
22	6	0	0.019762	1.588587	0.431461
23	8	0	0.403174	2.464393	1.152938
24	8	0	0.335189	1.479005	-0.860472
25	6	0	1.302676	2.423942	-1.328612
26	1	0	0.901593	3.435454	-1.260448
27	1	0	2.209287	2.350330	-0.726790
28	1	0	1.502302	2.151381	-2.361505
29	6	0	-4.183432	-0.581308	0.652200
30	6	0	-3.421193	-0.500721	-0.486924
31	1	0	-4.496346	-1.560786	0.985772
32	1	0	-4.839424	0.254740	0.857454
33	1	0	-2.971469	-1.355049	-0.984396
34	8	0	-3.158128	0.694648	-0.965976
35	6	0	-2.406668	0.746230	-2.190195
36	1	0	-2.089050	1.779076	-2.302242
37	1	0	-1.538323	0.088145	-2.130072
38	1	0	-3.058058	0.462360	-3.018590

TS3″ (NIMAG=1; 349.7*i* cm^{-1})

Center Number	Atomic Number	Atomic Type	Coordinates (Angstroms) X	Y	Z
1	6	0	-2.336277	-1.005809	2.323147
2	6	0	-2.061745	0.282362	1.890719
3	6	0	-1.086921	0.462854	0.893449
4	1	0	-2.652819	1.134280	2.197935
5	16	0	0.446275	-0.466802	-0.984971
6	8	0	0.346288	0.838851	-1.634449
7	8	0	0.158045	-1.654547	-1.775684
8	6	0	2.087037	-0.629789	-0.316312
9	6	0	2.478863	-1.862755	0.192972
10	6	0	2.933503	0.469462	-0.319046
11	6	0	3.755361	-1.989078	0.723519
12	1	0	1.794901	-2.701994	0.157494
13	6	0	4.211843	0.327438	0.210044
14	1	0	2.589417	1.404057	-0.743543
15	6	0	4.618051	-0.895423	0.733087
16	1	0	4.080686	-2.941503	1.123581
17	1	0	4.891484	1.170862	0.209942
18	1	0	5.614635	-1.000374	1.145071
19	7	0	-0.530159	-0.594657	0.342753
20	1	0	-1.549059	-1.742932	2.230413
21	6	0	-3.498895	-2.043945	0.891458
22	6	0	-2.999841	-1.561811	-0.291022
23	1	0	-3.282443	-3.069032	1.157657
24	1	0	-4.421949	-1.609952	1.253055
25	1	0	-2.254674	-2.076630	-0.892220
26	8	0	-3.377811	-0.365255	-0.675909
27	6	0	-2.843556	0.158329	-1.902512
28	1	0	-3.674212	0.277653	-2.597238
29	1	0	-2.395374	1.124474	-1.679198
30	1	0	-2.096730	-0.520396	-2.316982

31	1	0	-3.041598	-1.153856	3.132262
32	6	0	-0.796189	1.897800	0.494073
33	8	0	-1.617497	2.653628	0.059331
34	8	0	0.469517	2.211739	0.767101
35	6	0	0.893045	3.478203	0.254192
36	1	0	0.256926	4.276670	0.634363
37	1	0	1.917713	3.606387	0.593777
38	1	0	0.839797	3.451358	-0.835829

TS4″ (NIMAG=1; 380.1*i* cm^{-1})

Center Number	Atomic Number	Atomic Type	Coordinates (Angstroms) X	Y	Z
1	6	0	2.801953	0.845776	-2.007532
2	6	0	1.836535	1.494072	-1.245525
3	6	0	0.873308	0.701248	-0.595075
4	1	0	1.877402	2.555366	-1.042878
5	16	0	-0.131661	-1.708919	-0.126124
6	8	0	0.191416	-2.053240	1.252464
7	8	0	-0.159745	-2.761918	-1.120473
8	6	0	-1.749301	-0.940867	-0.139775
9	6	0	-2.256158	-0.466772	-1.344387
10	6	0	-2.480368	-0.882011	1.038571
11	6	0	-3.528795	0.091538	-1.362434
12	1	0	-1.662523	-0.537245	-2.248869
13	6	0	-3.760628	-0.340293	1.004235
14	1	0	-2.034545	-1.246621	1.955535
15	6	0	-4.281056	0.146651	-0.191698
16	1	0	-3.937593	0.469748	-2.291330
17	1	0	-4.349432	-0.294733	1.912337
18	1	0	-5.278402	0.570139	-0.212738
19	7	0	0.964064	-0.604622	-0.693088
20	1	0	2.519775	-0.102409	-2.448375
21	1	0	3.518469	1.440512	-2.561825
22	6	0	-0.111685	1.416346	0.305217
23	8	0	-0.268497	1.158246	1.466872
24	8	0	-0.759845	2.376647	-0.350182
25	6	0	-1.762748	3.055915	0.416034
26	1	0	-2.503364	2.335841	0.766296
27	1	0	-1.309459	3.557804	1.270822
28	1	0	-2.212053	3.774930	-0.262809
29	6	0	4.197882	-0.090320	-0.800258
30	6	0	3.476133	-0.475760	0.304210
31	1	0	4.565011	-0.863708	-1.460466
32	1	0	4.794082	0.807263	-0.697900
33	1	0	3.104241	-1.482405	0.472895
34	8	0	3.145671	0.450926	1.172236
35	6	0	2.449733	0.017039	2.356012
36	1	0	2.014741	0.910017	2.793498
37	1	0	1.659793	-0.687234	2.092367
38	1	0	3.171134	-0.432854	3.040120

TS5″ (NIMAG=1; 440.5*i* cm^{-1})

Center Number	Atomic Number	Atomic Type	Coordinates (Angstroms) X	Y	Z
1	6	0	-2.012639	-1.463636	2.204544
2	6	0	-1.906713	-0.102540	1.884999
3	6	0	-0.952297	0.275753	0.934930

4	1	0	-2.629147	0.623818	2.234048
5	16	0	0.730013	-0.335526	-0.933065
6	8	0	0.400905	0.962356	-1.511778
7	8	0	0.636575	-1.527013	-1.763890
8	6	0	2.390822	-0.253651	-0.290626
9	6	0	3.228716	-1.348500	-0.449966
10	6	0	2.800064	0.906568	0.357793
11	6	0	4.522096	-1.276736	0.056486
12	1	0	2.866653	-2.224045	-0.974296
13	6	0	4.093126	0.960449	0.861703
14	1	0	2.115977	1.741080	0.464772
15	6	0	4.950487	-0.126865	0.711094
16	1	0	5.194530	-2.117417	-0.063543
17	1	0	4.433194	1.854346	1.370088
18	1	0	5.959009	-0.075348	1.104163
19	7	0	-0.182058	-0.657888	0.408925
20	1	0	-1.090887	-2.032362	2.181937
21	6	0	-2.794496	-2.541742	0.714377
22	6	0	-2.263041	-1.991874	-0.442619
23	1	0	-2.426620	-3.525333	0.975628
24	1	0	-3.837912	-2.362063	0.940968
25	1	0	-1.381053	-2.374665	-0.941862
26	8	0	-2.731159	-0.913560	-1.024062
27	6	0	-4.014019	-0.396508	-0.660252
28	1	0	-4.110012	0.535662	-1.207958
29	1	0	-4.788397	-1.104992	-0.966693
30	1	0	-4.072427	-0.198827	0.407438
31	1	0	-2.719776	-1.757626	2.972860
32	6	0	-0.904646	1.744987	0.557078
33	8	0	-0.055872	2.501130	0.935788
34	8	0	-1.966482	2.095409	-0.169672
35	6	0	-1.894328	3.420913	-0.709252
36	1	0	-1.008283	3.497205	-1.339896
37	1	0	-2.799246	3.550970	-1.296550
38	1	0	-1.841930	4.156937	0.092635

TS6″ (NIMAG=1; 440.5i cm^{-1})

Center Number	Atomic Number	Atomic Type	Coordinates (Angstroms) X	Y	Z
1	6	0	-3.065517	-0.157954	1.881329
2	6	0	-2.106003	0.826002	1.598208
3	6	0	-0.947568	0.423208	0.921118
4	1	0	-2.285549	1.879881	1.762802
5	16	0	0.364509	-1.619553	-0.215849
6	8	0	0.027110	-1.589851	-1.632047
7	8	0	0.524164	-2.906223	0.433245
8	6	0	1.914940	-0.749116	0.002446
9	6	0	2.325119	-0.378179	1.276755
10	6	0	2.716514	-0.547232	-1.113548
11	6	0	3.563174	0.236800	1.429594
12	1	0	1.685603	-0.562113	2.133180
13	6	0	3.963604	0.043358	-0.944222
14	1	0	2.350153	-0.847173	-2.088232
15	6	0	4.382578	0.440025	0.323007
16	1	0	3.889864	0.548015	2.414022
17	1	0	4.606053	0.199355	-1.802642
18	1	0	5.351564	0.907977	0.449467
19	7	0	-0.822720	-0.853020	0.631442
20	1	0	-2.674832	-1.141203	2.116958
21	1	0	-3.953932	0.135361	2.430232

22	6	0	-0.025286	1.544852	0.495584
23	8	0	0.324671	2.428009	1.227483
24	8	0	0.298981	1.452859	-0.792529
25	6	0	1.265037	2.410340	-1.237451
26	1	0	0.854507	3.418040	-1.167603
27	1	0	2.162837	2.339813	-0.621334
28	1	0	1.483300	2.148293	-2.268944
29	6	0	-3.954866	-0.857933	0.240496
30	6	0	-3.001131	-0.786841	-0.765722
31	1	0	-4.226506	-1.857506	0.553634
32	1	0	-4.767854	-0.142792	0.226898
33	1	0	-2.409502	-1.625290	-1.107046
34	8	0	-2.673904	0.327120	-1.376865
35	6	0	-3.526470	1.470619	-1.281234
36	1	0	-2.991860	2.269193	-1.788037
37	1	0	-4.468278	1.263027	-1.796219
38	1	0	-3.711609	1.748097	-0.246102

TS7″ (NIMAG=1; 424.5*i* cm^{-1})

Center Number	Atomic Number	Atomic Type	Coordinates (Angstroms) X	Y	Z
1	6	0	-2.506550	-1.086062	2.104871
2	6	0	-2.098780	0.217558	1.796054
3	6	0	-1.046226	0.386592	0.890702
4	1	0	-2.670801	1.084268	2.099317
5	16	0	0.516364	-0.586881	-0.935495
6	8	0	0.331808	0.660399	-1.670643
7	8	0	0.300284	-1.848295	-1.627818
8	6	0	2.176684	-0.601494	-0.287448
9	6	0	2.626106	-1.738505	0.374323
10	6	0	2.985413	0.511019	-0.466893
11	6	0	3.918760	-1.750003	0.880405
12	1	0	1.970621	-2.594570	0.478727
13	6	0	4.281212	0.484742	0.038098
14	1	0	2.597134	1.366423	-1.005486
15	6	0	4.743318	-0.639768	0.713221
16	1	0	4.286524	-2.626646	1.399476
17	1	0	4.930655	1.340871	-0.099255
18	1	0	5.752832	-0.655242	1.106515
19	7	0	-0.433709	-0.680970	0.414333
20	1	0	-1.736678	-1.848048	2.100781
21	6	0	-3.476869	-1.948718	0.565047
22	6	0	-2.766544	-1.536555	-0.550546
23	1	0	-3.369328	-2.991982	0.831493
24	1	0	-4.451491	-1.516709	0.753270
25	1	0	-1.970903	-2.120199	-0.999953
26	8	0	-2.912311	-0.371713	-1.130095
27	6	0	-4.036617	0.464528	-0.831077
28	1	0	-3.808708	1.420796	-1.291235
29	1	0	-4.934878	0.021261	-1.268609
30	1	0	-4.154372	0.601697	0.240717
31	1	0	-3.291737	-1.222335	2.840735
32	6	0	-0.767031	1.814548	0.463514
33	8	0	-1.616425	2.567776	0.077673
34	8	0	0.512336	2.131329	0.652198
35	6	0	0.909520	3.380770	0.082513
36	1	0	0.298106	4.192168	0.475949
37	1	0	1.952335	3.513356	0.359635
38	1	0	0.794908	3.323892	-1.001715

TS8″ (NIMAG=1; 453.1i cm^{-1})

Center Number	Atomic Number	Atomic Type	Coordinates (Angstroms) X	Y	Z
1	6	0	-3.459029	-0.013538	1.283857
2	6	0	-2.385076	0.884836	1.347343
3	6	0	-1.101502	0.389734	1.084762
4	1	0	-2.525538	1.952103	1.452992
5	16	0	0.339422	-1.749112	0.321191
6	8	0	-0.137635	-2.582360	-0.774673
7	8	0	0.908263	-2.375155	1.497476
8	6	0	1.614688	-0.705526	-0.390222
9	6	0	2.913863	-0.850544	0.080491
10	6	0	1.310909	0.137276	-1.452179
11	6	0	3.934282	-0.132368	-0.532752
12	1	0	3.102320	-1.518468	0.912289
13	6	0	2.337270	0.869193	-2.040821
14	1	0	0.290402	0.223065	-1.808511
15	6	0	3.646053	0.728275	-1.588375
16	1	0	4.954369	-0.244135	-0.185128
17	1	0	2.115187	1.540445	-2.861347
18	1	0	4.443281	1.289589	-2.061093
19	7	0	-0.987647	-0.875243	0.747360
20	1	0	-3.242779	-1.031543	1.587457
21	1	0	-4.447960	0.349301	1.542533
22	6	0	0.009231	1.415483	1.095396
23	8	0	-0.066325	2.472049	0.528034
24	8	0	1.042840	1.016248	1.825804
25	6	0	2.177743	1.889895	1.795305
26	1	0	2.491772	2.045438	0.762157
27	1	0	1.922467	2.847436	2.249405
28	1	0	2.952612	1.383057	2.363103
29	6	0	-3.855635	-0.589389	-0.580434
30	6	0	-2.624434	-0.634766	-1.215973
31	1	0	-4.321211	-1.549923	-0.402811
32	1	0	-4.535697	0.215845	-0.828386
33	1	0	-2.047299	-1.541679	-1.354184
34	8	0	-2.005829	0.434987	-1.674394
35	6	0	-2.696110	1.683981	-1.795775
36	1	0	-1.925554	2.418564	-2.011585
37	1	0	-3.411182	1.619778	-2.619949
38	1	0	-3.196439	1.952213	-0.869226

TS9″ (NIMAG=1; 396.4i cm^{-1})

Center Number	Atomic Number	Atomic Type	Coordinates (Angstroms) X	Y	Z
1	6	0	2.431094	1.396911	1.873402
2	6	0	2.438519	0.048835	1.540613
3	6	0	1.461980	-0.456367	0.670870
4	1	0	3.275886	-0.585901	1.800371
5	16	0	-0.452579	-0.182794	-1.044565
6	8	0	-0.081979	-1.506509	-1.524708
7	8	0	-0.549835	0.909457	-2.003640
8	6	0	-2.018354	-0.305000	-0.201419
9	6	0	-3.034738	0.581827	-0.527647
10	6	0	-2.181546	-1.303570	0.753826
11	6	0	-4.255501	0.468755	0.131253
12	1	0	-2.866211	1.327688	-1.295018

13	6	0	-3.403843	-1.400043	1.404577
14	1	0	-1.366982	-1.984935	0.978893
15	6	0	-4.436235	-0.516682	1.094838
16	1	0	-5.063974	1.147056	-0.112636
17	1	0	-3.553039	-2.168784	2.152737
18	1	0	-5.387930	-0.601391	1.605855
19	7	0	0.560190	0.356303	0.155373
20	1	0	1.480935	1.916642	1.940417
21	1	0	3.208671	1.761409	2.535402
22	6	0	1.540586	-1.947624	0.385476
23	8	0	0.851962	-2.751861	0.948919
24	8	0	2.513004	-2.234539	-0.470466
25	6	0	2.539960	-3.602021	-0.898897
26	1	0	1.591140	-3.839083	-1.380812
27	1	0	3.362442	-3.671955	-1.604708
28	1	0	2.698084	-4.263932	-0.047800
29	6	0	2.944035	2.551728	0.216593
30	6	0	1.721003	2.694858	-0.390219
31	1	0	3.270570	3.354245	0.866658
32	1	0	3.704694	1.977857	-0.292826
33	1	0	1.420063	2.173752	-1.293484
34	8	0	0.834234	3.494994	0.170442
35	6	0	-0.499519	3.480438	-0.351353
36	1	0	-0.488873	3.323041	-1.429235
37	1	0	-1.057285	2.674184	0.126182
38	1	0	-0.931992	4.446085	-0.100424

TS10″ (NIMAG=1; 362.4*i* cm^{-1})

Center Number	Atomic Number	Atomic Type	Coordinates (Angstroms) X	Y	Z
1	6	0	2.902702	1.833115	-1.157280
2	6	0	1.617073	2.249815	-0.849139
3	6	0	0.662567	1.291030	-0.464334
4	1	0	1.375642	3.296641	-0.723290
5	16	0	0.054660	-1.271895	-0.034848
6	8	0	0.324411	-1.569619	1.366708
7	8	0	0.335647	-2.297884	-1.024580
8	6	0	-1.686897	-0.899517	-0.207260
9	6	0	-2.161916	-0.378213	-1.403994
10	6	0	-2.537450	-1.246621	0.834317
11	6	0	-3.529671	-0.173036	-1.546473
12	1	0	-1.475365	-0.132015	-2.206573
13	6	0	-3.905259	-1.062577	0.668142
14	1	0	-2.118095	-1.647851	1.749296
15	6	0	-4.398292	-0.521385	-0.516255
16	1	0	-3.915502	0.250315	-2.465315
17	1	0	-4.585630	-1.337013	1.465328
18	1	0	-5.464385	-0.371701	-0.638140
19	7	0	0.985302	0.020115	-0.474309
20	1	0	3.047609	0.847249	-1.585605
21	1	0	3.639490	2.580886	-1.427557
22	6	0	-0.631143	1.881255	0.060119
23	8	0	-1.271525	2.706170	-0.526539
24	8	0	-0.918897	1.411963	1.273385
25	6	0	-2.161846	1.873756	1.814980
26	1	0	-2.114033	2.948281	1.992757
27	1	0	-2.971061	1.655092	1.117065
28	1	0	-2.295738	1.330662	2.746302
29	6	0	3.857930	1.213892	0.621054
30	6	0	3.414680	-0.078117	0.749457

31	1	0	4.841122	1.361571	0.190781
32	1	0	3.498984	1.951838	1.322895
33	1	0	2.604676	-0.371225	1.412414
34	8	0	3.978855	-1.023497	0.027897
35	6	0	3.371377	-2.323974	0.041484
36	1	0	2.687577	-2.404807	-0.802212
37	1	0	4.182210	-3.043699	-0.044847
38	1	0	2.819756	-2.478331	0.970532

TS11″ (NIMAG=1; 389.6i cm^{-1})

Center Number	Atomic Number	Atomic Type	Coordinates (Angstroms) X	Y	Z
1	6	0	2.883080	0.913631	-1.722323
2	6	0	1.904350	1.812524	-1.320715
3	6	0	0.844619	1.374478	-0.516508
4	1	0	2.018488	2.877648	-1.471984
5	16	0	-0.270801	-0.338290	1.069656
6	8	0	-0.715115	0.777264	1.895823
7	8	0	0.376335	-1.467297	1.726326
8	6	0	-1.679797	-0.970689	0.178619
9	6	0	-1.485596	-1.963525	-0.773958
10	6	0	-2.941312	-0.482851	0.484983
11	6	0	-2.590180	-2.469679	-1.445963
12	1	0	-0.484728	-2.318182	-0.990289
13	6	0	-4.041026	-1.004269	-0.188218
14	1	0	-3.043584	0.288167	1.239180
15	6	0	-3.864778	-1.991578	-1.151869
16	1	0	-2.458297	-3.238744	-2.197113
17	1	0	-5.035113	-0.639709	0.040843
18	1	0	-4.723860	-2.392162	-1.676589
19	7	0	0.795689	0.117540	-0.116840
20	1	0	2.614373	-0.126795	-1.872300
21	6	0	4.058419	0.400837	-0.076233
22	6	0	3.383844	-0.688925	0.412372
23	1	0	4.906614	0.209237	-0.721727
24	1	0	4.069812	1.304513	0.515394
25	1	0	2.762213	-0.674836	1.303063
26	8	0	3.436240	-1.818735	-0.265951
27	6	0	2.791619	-2.961658	0.314781
28	1	0	1.837781	-2.673447	0.756336
29	1	0	2.650784	-3.670977	-0.496926
30	1	0	3.447452	-3.397097	1.071057
31	1	0	3.689981	1.282177	-2.345575
32	6	0	-0.162684	2.446597	-0.136611
33	8	0	0.129802	3.519321	0.302457
34	8	0	-1.398018	2.053711	-0.454082
35	6	0	-2.439549	2.907481	0.024218
36	1	0	-2.308170	3.919613	-0.357321
37	1	0	-3.366941	2.472653	-0.340238
38	1	0	-2.413812	2.919440	1.115532

TS12″ (NIMAG=1; 367.5i cm^{-1})

Center Number	Atomic Number	Atomic Type	Coordinates (Angstroms) X	Y	Z
1	6	0	2.716589	2.071966	-1.015901
2	6	0	1.411210	2.333883	-0.626988
3	6	0	0.573345	1.255830	-0.289334

4	1	0	1.087008	3.338546	-0.388991
5	16	0	0.185595	-1.352089	-0.023790
6	8	0	0.549057	-1.709274	1.341034
7	8	0	0.473373	-2.293787	-1.092126
8	6	0	-1.578961	-1.067813	-0.087357
9	6	0	-2.158942	-0.749399	-1.310011
10	6	0	-2.333845	-1.248074	1.063172
11	6	0	-3.539262	-0.600938	-1.375564
12	1	0	-1.538962	-0.631120	-2.191305
13	6	0	-3.715370	-1.115585	0.980288
14	1	0	-1.832133	-1.477780	1.994895
15	6	0	-4.314416	-0.793308	-0.234454
16	1	0	-4.009617	-0.352089	-2.318883
17	1	0	-4.322964	-1.261712	1.864897
18	1	0	-5.391798	-0.691920	-0.293386
19	7	0	1.008666	0.027346	-0.429039
20	1	0	2.931863	1.146960	-1.539833
21	1	0	3.365513	2.908685	-1.248371
22	6	0	-0.712405	1.631539	0.417654
23	8	0	-0.941245	1.362357	1.562399
24	8	0	-1.518427	2.332781	-0.377797
25	6	0	-2.773479	2.695693	0.212454
26	1	0	-3.303020	1.795953	0.527822
27	1	0	-2.611037	3.343380	1.074002
28	1	0	-3.323260	3.216404	-0.566287
29	6	0	3.803344	1.402669	0.652596
30	6	0	3.472643	0.071205	0.701511
31	1	0	4.751439	1.660840	0.196144
32	1	0	3.424011	2.052127	1.427531
33	1	0	2.722635	-0.335546	1.375098
34	8	0	4.078209	-0.769442	-0.110370
35	6	0	3.571154	-2.110555	-0.175820
36	1	0	2.848670	-2.177623	-0.988048
37	1	0	4.427206	-2.754488	-0.363748
38	1	0	3.086899	-2.382366	0.763792

TS13″ (NIMAG=1; 456.8*i* cm^{-1})

Center Number	Atomic Number	Atomic Type	Coordinates (Angstroms) X	Y	Z
1	6	0	2.556233	-0.747113	-1.825631
2	6	0	2.235227	0.589416	-1.581357
3	6	0	1.180151	0.865258	-0.704893
4	1	0	2.881026	1.395268	-1.904704
5	16	0	-0.546135	0.123890	1.082666
6	8	0	-0.586931	1.525308	1.482271
7	8	0	-0.303492	-0.892535	2.092030
8	6	0	-2.075534	-0.263784	0.257636
9	6	0	-2.651385	-1.510267	0.458842
10	6	0	-2.640824	0.692682	-0.578543
11	6	0	-3.841713	-1.805340	-0.197125
12	1	0	-2.173147	-2.215052	1.128169
13	6	0	-3.826912	0.379893	-1.230121
14	1	0	-2.155802	1.653762	-0.712454
15	6	0	-4.424767	-0.863537	-1.038742
16	1	0	-4.314877	-2.768230	-0.047622
17	1	0	-4.287173	1.109196	-1.885374
18	1	0	-5.352574	-1.097881	-1.547200
19	7	0	0.578090	-0.145837	-0.108423
20	1	0	1.724782	-1.441596	-1.853851
21	1	0	3.367811	-0.961938	-2.512251

22	6	0	0.823146	2.326220	-0.493400
23	8	0	-0.079198	2.866691	-1.068582
24	8	0	1.678411	2.924633	0.328541
25	6	0	1.298424	4.251961	0.711632
26	1	0	0.329453	4.210302	1.209852
27	1	0	2.071410	4.593459	1.394006
28	1	0	1.237041	4.898198	-0.163704
29	6	0	3.370904	-1.570807	-0.172494
30	6	0	2.322326	-1.880222	0.680399
31	1	0	3.865465	-2.378664	-0.698600
32	1	0	4.013948	-0.763628	0.148241
33	1	0	2.030699	-1.263695	1.519079
34	8	0	1.571820	-2.959121	0.605029
35	6	0	1.894893	-3.960589	-0.352769
36	1	0	1.880812	-3.560861	-1.367185
37	1	0	2.877396	-4.388308	-0.135602
38	1	0	1.129368	-4.724141	-0.249409

TS14″ (NIMAG=1; 444.5*i* cm^{-1})

Center Number	Atomic Number	Atomic Type	Coordinates (Angstroms) X	Y	Z
1	6	0	2.767344	1.759492	-1.349126
2	6	0	1.551151	2.257627	-0.906291
3	6	0	0.613972	1.338489	-0.401516
4	1	0	1.374282	3.316914	-0.775939
5	16	0	0.119959	-1.228362	0.208871
6	8	0	0.220158	-1.330677	1.653998
7	8	0	0.591765	-2.324033	-0.618168
8	6	0	-1.599656	-0.963136	-0.215253
9	6	0	-1.937041	-0.575737	-1.506220
10	6	0	-2.564233	-1.221068	0.749387
11	6	0	-3.280101	-0.418699	-1.828558
12	1	0	-1.162041	-0.393918	-2.242473
13	6	0	-3.905042	-1.084198	0.407780
14	1	0	-2.251538	-1.514190	1.744769
15	6	0	-4.260674	-0.677438	-0.875225
16	1	0	-3.559949	-0.100914	-2.825193
17	1	0	-4.671858	-1.288764	1.145363
18	1	0	-5.306367	-0.562372	-1.134221
19	7	0	0.985573	0.083681	-0.328144
20	1	0	2.766014	0.785701	-1.819010
21	1	0	3.535478	2.449737	-1.679338
22	6	0	-0.674536	1.933450	0.126661
23	8	0	-1.284534	2.792617	-0.444157
24	8	0	-1.004800	1.419075	1.308294
25	6	0	-2.252160	1.875975	1.840691
26	1	0	-2.202345	2.946206	2.042257
27	1	0	-3.052498	1.676158	1.127082
28	1	0	-2.401256	1.313505	2.758149
29	6	0	3.849410	0.947703	0.275176
30	6	0	3.138366	-0.054200	0.909517
31	1	0	4.701023	0.681251	-0.338711
32	1	0	3.918444	1.885367	0.807017
33	1	0	2.509189	0.149733	1.769062
34	8	0	3.215471	-1.353063	0.689835
35	6	0	3.780557	-1.802418	-0.534266
36	1	0	4.840310	-1.539580	-0.597449
37	1	0	3.660200	-2.881631	-0.533563
38	1	0	3.215926	-1.381576	-1.367682

TS15″ (NIMAG=1; 450.9*i* cm^{-1})

Center Number	Atomic Number	Atomic Type	Coordinates (Angstroms) X	Y	Z
1	6	0	2.754815	0.788188	-1.652925
2	6	0	1.832740	1.783758	-1.324339
3	6	0	0.746228	1.444748	-0.512042
4	1	0	2.019285	2.828185	-1.535172
5	16	0	-0.414581	-0.172797	1.161290
6	8	0	-1.074208	0.994110	1.734592
7	8	0	0.303868	-1.075417	2.045233
8	6	0	-1.626927	-1.127446	0.274720
9	6	0	-1.271656	-2.397151	-0.166706
10	6	0	-2.885362	-0.591384	0.045155
11	6	0	-2.208598	-3.144994	-0.866405
12	1	0	-0.281917	-2.780859	0.052452
13	6	0	-3.816315	-1.353369	-0.653230
14	1	0	-3.119406	0.395918	0.422898
15	6	0	-3.477058	-2.622387	-1.109705
16	1	0	-1.954443	-4.138158	-1.216144
17	1	0	-4.807720	-0.957463	-0.836791
18	1	0	-4.206141	-3.211623	-1.653106
19	7	0	0.675617	0.219930	-0.025932
20	1	0	2.354240	-0.210425	-1.787499
21	6	0	3.844103	0.292687	-0.028331
22	6	0	3.059727	-0.587979	0.698850
23	1	0	4.682299	-0.099373	-0.591750
24	1	0	3.998063	1.265004	0.417868
25	1	0	2.478688	-0.302709	1.564364
26	8	0	2.921369	-1.872064	0.440270
27	6	0	3.737701	-2.466266	-0.563520
28	1	0	4.791953	-2.391818	-0.284305
29	1	0	3.440698	-3.510283	-0.604728
30	1	0	3.578741	-1.998950	-1.535972
31	1	0	3.580575	1.051300	-2.304923
32	6	0	-0.243992	2.559160	-0.229075
33	8	0	0.051452	3.619211	0.238485
34	8	0	-1.458117	2.224013	-0.674213
35	6	0	-2.500465	3.120109	-0.281861
36	1	0	-2.276462	4.135004	-0.608543
37	1	0	-3.404135	2.752092	-0.761602
38	1	0	-2.594899	3.094523	0.805391

TS16″ (NIMAG=1; 452.3*i* cm^{-1})

Center Number	Atomic Number	Atomic Type	Coordinates (Angstroms) X	Y	Z
1	6	0	-2.581049	2.054266	1.149474
2	6	0	-1.335196	2.369104	0.625139
3	6	0	-0.519318	1.303620	0.205126
4	1	0	-1.067531	3.382729	0.356306
5	16	0	-0.267007	-1.319715	-0.176612
6	8	0	-0.474566	-1.521478	-1.598620
7	8	0	-0.743843	-2.308027	0.772649
8	6	0	1.489477	-1.125438	0.108853
9	6	0	1.937546	-0.875195	1.400819
10	6	0	2.364791	-1.271905	-0.958405
11	6	0	3.304141	-0.760276	1.624776
12	1	0	1.225764	-0.776499	2.212451

13	6	0	3.731263	-1.176692	-0.717934
14	1	0	1.964408	-1.441884	-1.950182
15	6	0	4.197722	-0.919346	0.568196
16	1	0	3.671567	-0.561261	2.623965
17	1	0	4.430458	-1.297553	-1.536405
18	1	0	5.263454	-0.842828	0.749518
19	7	0	-1.011453	0.093042	0.297911
20	1	0	-2.659520	1.150610	1.737851
21	1	0	-3.261931	2.855479	1.414323
22	6	0	0.772868	1.646568	-0.503430
23	8	0	1.018082	1.345747	-1.636298
24	8	0	1.582329	2.343844	0.294007
25	6	0	2.859256	2.658050	-0.276107
26	1	0	3.378432	1.735629	-0.539129
27	1	0	2.733829	3.271780	-1.168127
28	1	0	3.398832	3.199786	0.495365
29	6	0	-3.788621	1.177066	-0.332552
30	6	0	-3.198094	0.045515	-0.867001
31	1	0	-4.642365	1.063552	0.324404
32	1	0	-3.790534	2.049268	-0.969978
33	1	0	-2.582039	0.089994	-1.758676
34	8	0	-3.394138	-1.207395	-0.501029
35	6	0	-3.959782	-1.459452	0.777497
36	1	0	-4.986836	-1.087668	0.833698
37	1	0	-3.944948	-2.538727	0.897131
38	1	0	-3.331690	-1.005084	1.545514

TS17″ (NIMAG=1; 496.6i cm^{-1})

Center Number	Atomic Number	Atomic Type	Coordinates (Angstroms) X	Y	Z
1	6	0	1.812389	-1.680739	-1.779413
2	6	0	1.640614	-0.310686	-1.764982
3	6	0	0.821562	0.234870	-0.769414
4	1	0	2.288614	0.350007	-2.329067
5	16	0	-0.889581	-0.085104	1.183130
6	8	0	-0.709455	1.322093	1.503543
7	8	0	-0.891423	-1.076196	2.240113
8	6	0	-2.397556	-0.245132	0.248440
9	6	0	-3.231352	-1.324480	0.503294
10	6	0	-2.696452	0.720510	-0.706881
11	6	0	-4.410883	-1.437591	-0.224887
12	1	0	-2.957969	-2.042747	1.266355
13	6	0	-3.874630	0.587989	-1.429827
14	1	0	-2.021605	1.552409	-0.880051
15	6	0	-4.728075	-0.486049	-1.188333
16	1	0	-5.081365	-2.267210	-0.037213
17	1	0	-4.128602	1.326933	-2.179654
18	1	0	-5.647654	-0.579359	-1.753765
19	7	0	0.226994	-0.622988	0.045412
20	1	0	1.002587	-2.311026	-1.437183
21	1	0	2.517390	-2.129514	-2.470660
22	6	0	0.855282	1.734381	-0.561613
23	8	0	0.207275	2.509925	-1.208569
24	8	0	1.729540	2.060551	0.381980
25	6	0	1.653925	3.421629	0.822131
26	1	0	2.422953	3.526885	1.581800
27	1	0	1.830984	4.102034	-0.010350
28	1	0	0.664061	3.596864	1.244728
29	6	0	2.784455	-1.904251	0.176931
30	6	0	1.714170	-1.812499	1.040028

31	1	0	3.129899	-2.863265	-0.200136
32	1	0	1.747495	-1.065959	1.822290
33	1	0	1.057590	-2.657214	1.185180
34	8	0	3.698985	-0.898170	0.246893
35	6	0	4.779722	-1.010865	-0.652372
36	1	0	4.440013	-0.906106	-1.688485
37	1	0	5.471356	-0.205184	-0.417076
38	1	0	5.290175	-1.973107	-0.534972

TS18″ (NIMAG=1; 463.3i cm^{-1})

Center Number	Atomic Number	Atomic Type	Coordinates (Angstroms) X	Y	Z
1	6	0	-2.706901	-0.397129	1.974678
2	6	0	-1.868794	0.637897	1.634342
3	6	0	-0.810624	0.377132	0.744424
4	1	0	-2.115210	1.672246	1.840490
5	16	0	0.597121	-1.538413	-0.538516
6	8	0	0.434419	-1.320603	-1.963825
7	8	0	0.681435	-2.898618	-0.046719
8	6	0	2.044115	-0.659019	0.026726
9	6	0	2.356191	-0.674299	1.381077
10	6	0	2.849844	-0.028852	-0.910435
11	6	0	3.501244	-0.013853	1.807022
12	1	0	1.710960	-1.191122	2.081989
13	6	0	4.001660	0.615600	-0.472124
14	1	0	2.561564	-0.049630	-1.954818
15	6	0	4.320818	0.627847	0.882128
16	1	0	3.756894	-0.004481	2.859192
17	1	0	4.647337	1.110035	-1.187873
18	1	0	5.214934	1.138027	1.219730
19	7	0	-0.676361	-0.878922	0.357473
20	1	0	-2.322590	-1.408593	1.976666
21	6	0	-3.452834	-0.797091	-0.129999
22	6	0	-2.417825	-1.551246	-0.637091
23	1	0	-4.299346	-1.237394	0.387691
24	1	0	-1.963450	-1.236867	-1.568605
25	1	0	-2.325581	-2.590495	-0.352692
26	8	0	-3.623859	0.430899	-0.681635
27	6	0	-4.690210	1.182638	-0.139544
28	1	0	-4.502334	1.415855	0.913844
29	1	0	-4.748971	2.105791	-0.711240
30	1	0	-5.635908	0.638226	-0.228740
31	1	0	-3.597108	-0.205879	2.563977
32	6	0	-0.123346	1.588413	0.175401
33	8	0	0.048579	2.601392	0.795895
34	8	0	0.224639	1.410964	-1.096987
35	6	0	0.906659	2.509839	-1.703666
36	1	0	1.120122	2.193696	-2.721259
37	1	0	0.270358	3.394894	-1.697524
38	1	0	1.827237	2.723232	-1.158360

TS19″ (NIMAG=1; 508.9i cm^{-1})

Center Number	Atomic Number	Atomic Type	Coordinates (Angstroms) X	Y	Z
1	6	0	-2.099844	-1.112552	1.944986
2	6	0	-1.885535	0.210077	1.600124
3	6	0	-0.973136	0.483552	0.576028

4	1	0	-2.564276	0.994252	1.913769
5	16	0	0.807956	-0.348691	-1.155326
6	8	0	0.755673	0.989143	-1.722422
7	8	0	0.720240	-1.507816	-2.020954
8	6	0	2.303488	-0.496660	-0.195468
9	6	0	3.039896	-1.669323	-0.286540
10	6	0	2.696981	0.567115	0.608478
11	6	0	4.212825	-1.776747	0.453419
12	1	0	2.698412	-2.465174	-0.936660
13	6	0	3.866464	0.441466	1.346340
14	1	0	2.091395	1.465041	0.653679
15	6	0	4.621840	-0.726217	1.267480
16	1	0	4.806871	-2.680210	0.390933
17	1	0	4.191899	1.256790	1.980696
18	1	0	5.535871	-0.815361	1.842452
19	7	0	-0.354114	-0.560677	0.040981
20	1	0	-1.279372	-1.812418	1.854873
21	1	0	-2.861792	-1.357995	2.676877
22	6	0	-0.977513	1.889673	0.003204
23	8	0	-1.847538	2.312641	-0.697307
24	8	0	0.058679	2.606587	0.451879
25	6	0	0.223981	3.871460	-0.200325
26	1	0	1.094631	4.330708	0.260261
27	1	0	0.389859	3.699737	-1.264381
28	1	0	-0.661088	4.490747	-0.058887
29	6	0	-2.956564	-1.819139	0.104370
30	6	0	-1.841472	-1.971408	-0.691841
31	1	0	-3.347539	-2.650545	0.686385
32	1	0	-1.807428	-1.459645	-1.643494
33	1	0	-1.193016	-2.824889	-0.562135
34	8	0	-3.846323	-0.867963	-0.293426
35	6	0	-4.973288	-0.714109	0.539917
36	1	0	-5.496387	-1.667649	0.674114
37	1	0	-4.686662	-0.317867	1.520078
38	1	0	-5.634468	-0.005580	0.046607

TS20″ (NIMAG=1; 455.9i cm^{-1})

Center Number	Atomic Number	Atomic Type	Coordinates (Angstroms) X	Y	Z
1	6	0	-3.089956	-1.023197	1.203600
2	6	0	-2.777102	0.173803	0.610988
3	6	0	-1.424043	0.570980	0.570206
4	1	0	-3.496382	0.722449	0.016338
5	16	0	1.031303	0.113964	1.533108
6	8	0	1.354358	-0.915761	2.500029
7	8	0	1.071295	1.519532	1.887830
8	6	0	2.152315	-0.157242	0.164715
9	6	0	2.632287	0.928518	-0.555726
10	6	0	2.627763	-1.446991	-0.054017
11	6	0	3.576437	0.704510	-1.550428
12	1	0	2.266784	1.920628	-0.333045
13	6	0	3.561831	-1.658938	-1.059884
14	1	0	2.289842	-2.259984	0.576699
15	6	0	4.030495	-0.584442	-1.809866
16	1	0	3.960841	1.541175	-2.120600
17	1	0	3.936814	-2.658177	-1.243799
18	1	0	4.765346	-0.750971	-2.588608
19	7	0	-0.558390	-0.264307	1.102693
20	1	0	-2.438888	-1.414350	1.974190
21	1	0	-4.106530	-1.399630	1.171614

22	6	0	-1.042221	1.757976	-0.266746
23	8	0	-0.022181	1.847940	-0.890805
24	8	0	-1.999603	2.688294	-0.255953
25	6	0	-1.715744	3.851630	-1.038622
26	1	0	-2.580639	4.499167	-0.927420
27	1	0	-0.816154	4.340289	-0.664730
28	1	0	-1.566449	3.576271	-2.082837
29	6	0	-1.943392	-2.358310	-0.263598
30	6	0	-0.661805	-2.193478	0.200776
31	1	0	-2.612679	-3.122777	0.118728
32	1	0	0.083331	-1.859421	-0.509324
33	1	0	-0.333921	-2.715945	1.088739
34	8	0	-2.216863	-1.843551	-1.490401
35	6	0	-3.552739	-2.005035	-1.918855
36	1	0	-3.841897	-3.061133	-1.909688
37	1	0	-4.235920	-1.436051	-1.279253
38	1	0	-3.606404	-1.625141	-2.936453

TS21″ (NIMAG=1; 546.4*i* cm^{-1})

Center Number	Atomic Number	Atomic Type	Coordinates (Angstroms) X	Y	Z
1	6	0	-1.816116	-1.546162	2.046428
2	6	0	-1.695579	-0.167546	1.890016
3	6	0	-0.860929	0.301356	0.876818
4	1	0	-2.383409	0.516493	2.371525
5	16	0	0.763184	-0.164158	-1.119701
6	8	0	0.534265	1.214203	-1.528556
7	8	0	0.686685	-1.229795	-2.099929
8	6	0	2.350109	-0.249825	-0.316391
9	6	0	3.153801	-1.358574	-0.543229
10	6	0	2.737454	0.795031	0.515627
11	6	0	4.392796	-1.419505	0.084976
12	1	0	2.811777	-2.139432	-1.211114
13	6	0	3.975346	0.714450	1.140216
14	1	0	2.083946	1.646735	0.670098
15	6	0	4.798954	-0.387721	0.924766
16	1	0	5.040352	-2.270987	-0.084294
17	1	0	4.298605	1.515847	1.792942
18	1	0	5.764981	-0.440350	1.412681
19	7	0	-0.261015	-0.616046	0.128962
20	1	0	-0.934570	-2.152263	1.874810
21	1	0	-2.530062	-1.923511	2.769634
22	6	0	-0.839003	1.791454	0.596528
23	8	0	-0.076887	2.553086	1.120842
24	8	0	-1.805450	2.140461	-0.249583
25	6	0	-1.709311	3.482134	-0.745797
26	1	0	-2.549960	3.606600	-1.422306
27	1	0	-1.760545	4.195408	0.076002
28	1	0	-0.763299	3.589361	-1.277075
29	6	0	-2.748701	-2.190422	0.333359
30	6	0	-1.760327	-2.048749	-0.628520
31	1	0	-2.857484	-3.157728	0.810618
32	1	0	-1.881074	-1.427882	-1.504399
33	1	0	-0.988707	-2.801426	-0.688450
34	8	0	-3.938271	-1.517835	0.330894
35	6	0	-4.071323	-0.468124	-0.611693
36	1	0	-4.097176	-0.863653	-1.633039
37	1	0	-5.020276	0.015503	-0.389833
38	1	0	-3.262696	0.262388	-0.523290

TS22″ (NIMAG=1; 504.0*i* cm^{-1})

Center Number	Atomic Number	Atomic Type	Coordinates (Angstroms) X	Y	Z
1	6	0	-2.752564	-0.307762	2.106204
2	6	0	-1.835975	0.690970	1.822594
3	6	0	-0.799996	0.403233	0.925534
4	1	0	-2.018047	1.724157	2.088120
5	16	0	0.364857	-1.459739	-0.627683
6	8	0	0.101174	-1.045402	-1.995329
7	8	0	0.374852	-2.875022	-0.318620
8	6	0	1.921530	-0.763259	-0.100699
9	6	0	2.321771	-0.927583	1.220479
10	6	0	2.715818	-0.119563	-1.038742
11	6	0	3.547787	-0.407320	1.613684
12	1	0	1.680744	-1.448027	1.922686
13	6	0	3.948759	0.381927	-0.635242
14	1	0	2.356404	-0.018206	-2.056126
15	6	0	4.358294	0.243715	0.687322
16	1	0	3.872741	-0.513845	2.641041
17	1	0	4.587020	0.883208	-1.352926
18	1	0	5.315646	0.643800	0.998868
19	7	0	-0.774746	-0.825687	0.435476
20	1	0	-2.404954	-1.332862	2.140586
21	6	0	-3.650410	-0.630872	0.197673
22	6	0	-2.678729	-1.389730	-0.431753
23	1	0	-4.428937	-1.125914	0.764703
24	1	0	-2.281395	-1.124000	-1.402059
25	1	0	-2.567563	-2.422594	-0.131114
26	8	0	-4.045773	0.600551	-0.225232
27	6	0	-3.363117	1.144014	-1.344813
28	1	0	-3.606086	0.584219	-2.254150
29	1	0	-3.718313	2.167329	-1.443403
30	1	0	-2.278731	1.142603	-1.206630
31	1	0	-3.626849	-0.065063	2.699078
32	6	0	0.023993	1.585984	0.482962
33	8	0	0.411309	2.434482	1.235426
34	8	0	0.218140	1.598092	-0.837119
35	6	0	1.012105	2.682583	-1.326399
36	1	0	1.109300	2.513211	-2.395404
37	1	0	0.513756	3.631181	-1.125661
38	1	0	1.987554	2.676815	-0.838860

TS23″ (NIMAG=1; 545.4*i* cm^{-1})

Center Number	Atomic Number	Atomic Type	Coordinates (Angstroms) X	Y	Z
1	6	0	-2.056429	-1.097826	2.138042
2	6	0	-1.889718	0.230584	1.750831
3	6	0	-0.995016	0.502762	0.717892
4	1	0	-2.591555	0.996681	2.055830
5	16	0	0.687753	-0.351365	-1.100336
6	8	0	0.589856	0.977541	-1.685313
7	8	0	0.532469	-1.522032	-1.941950
8	6	0	2.255441	-0.485673	-0.262368
9	6	0	2.987781	-1.656162	-0.401639
10	6	0	2.705944	0.585772	0.500579
11	6	0	4.215374	-1.753158	0.245351
12	1	0	2.600294	-2.458684	-1.016823

13	6	0	3.929911	0.470692	1.145723
14	1	0	2.102125	1.481807	0.586509
15	6	0	4.682024	-0.694729	1.016968
16	1	0	4.806556	-2.654915	0.143659
17	1	0	4.300135	1.292122	1.746744
18	1	0	5.638399	-0.775814	1.519680
19	7	0	-0.383791	-0.544041	0.173307
20	1	0	-1.186829	-1.744155	2.118517
21	1	0	-2.810302	-1.325620	2.883063
22	6	0	-1.018258	1.903465	0.133524
23	8	0	-1.931873	2.326727	-0.515331
24	8	0	0.047041	2.615644	0.499585
25	6	0	0.183986	3.869238	-0.182611
26	1	0	1.087309	4.324140	0.214587
27	1	0	0.280129	3.677276	-1.251682
28	1	0	-0.683415	4.500546	0.005962
29	6	0	-2.945870	-1.982309	0.528850
30	6	0	-1.916498	-2.039586	-0.399302
31	1	0	-3.113412	-2.855337	1.150029
32	1	0	-1.976442	-1.569397	-1.369858
33	1	0	-1.175280	-2.817955	-0.297675
34	8	0	-4.105712	-1.282206	0.359867
35	6	0	-4.152772	-0.377514	-0.732588
36	1	0	-3.335494	0.348387	-0.699068
37	1	0	-4.129563	-0.917528	-1.685124
38	1	0	-5.099818	0.149838	-0.642490

TS24″ (NIMAG=1; 559.9*i* cm^{-1})

Center Number	Atomic Number	Atomic Type	Coordinates (Angstroms) X	Y	Z
1	6	0	-2.134961	-2.255462	1.368056
2	6	0	-2.525968	-1.195487	0.552481
3	6	0	-1.667305	-0.101853	0.453150
4	1	0	-3.342160	-1.297145	-0.151888
5	16	0	0.470814	1.183712	1.233165
6	8	0	0.951770	1.217894	2.596941
7	8	0	-0.209064	2.335944	0.661014
8	6	0	1.863976	0.772490	0.196701
9	6	0	1.756532	0.916896	-1.182433
10	6	0	3.016499	0.286554	0.801974
11	6	0	2.837238	0.542469	-1.972653
12	1	0	0.844895	1.306842	-1.618722
13	6	0	4.090131	-0.079679	-0.002648
14	1	0	3.060810	0.212538	1.881770
15	6	0	3.996900	0.041991	-1.385440
16	1	0	2.775240	0.648431	-3.048846
17	1	0	4.998746	-0.455996	0.451058
18	1	0	4.834995	-0.246001	-2.008905
19	7	0	-0.535213	-0.165177	1.138491
20	1	0	-1.607856	-2.015131	2.284156
21	1	0	-2.752817	-3.146141	1.385002
22	6	0	-1.960823	0.915711	-0.628049
23	8	0	-1.360418	0.964726	-1.670091
24	8	0	-3.007326	1.663624	-0.320471
25	6	0	-3.299286	2.706642	-1.258942
26	1	0	-4.166795	3.223692	-0.860122
27	1	0	-2.442981	3.377835	-1.325482
28	1	0	-3.512402	2.284162	-2.240624
29	6	0	-0.482894	-3.022210	0.447848
30	6	0	0.490369	-2.077721	0.736420

31	1	0	-0.574721	-3.880907	1.103008
32	1	0	1.056099	-1.589461	-0.042887
33	1	0	0.916203	-2.069368	1.730089
34	8	0	-0.893459	-3.327792	-0.819470
35	6	0	-0.437290	-2.477211	-1.858612
36	1	0	-0.658963	-1.424602	-1.657363
37	1	0	0.641009	-2.595923	-2.014837
38	1	0	-0.966566	-2.789919	-2.755989

TS25″ (NIMAG=1; 525.3i cm^{-1})

Center Number	Atomic Number	Atomic Type	Coordinates (Angstroms) X	Y	Z
1	6	0	2.385616	-0.510268	-1.703893
2	6	0	1.688105	0.684290	-1.746340
3	6	0	0.670817	0.880920	-0.808513
4	1	0	2.032367	1.525797	-2.335601
5	16	0	-0.776014	0.016612	1.186226
6	8	0	-1.188503	1.395780	1.400739
7	8	0	-0.385336	-0.804531	2.314190
8	6	0	-2.076213	-0.815136	0.298744
9	6	0	-2.347416	-2.142658	0.598966
10	6	0	-2.778092	-0.109902	-0.672717
11	6	0	-3.364870	-2.784810	-0.097986
12	1	0	-1.777653	-2.643663	1.371761
13	6	0	-3.786667	-0.768964	-1.363717
14	1	0	-2.537227	0.927147	-0.880964
15	6	0	-4.078628	-2.099966	-1.076049
16	1	0	-3.600208	-3.818225	0.124794
17	1	0	-4.347759	-0.240902	-2.124710
18	1	0	-4.869892	-2.605335	-1.616951
19	7	0	0.477703	-0.093859	0.068647
20	1	0	1.862336	-1.404841	-1.389582
21	1	0	3.235934	-0.646164	-2.362754
22	6	0	0.068424	2.266559	-0.699876
23	8	0	-0.854831	2.655610	-1.357173
24	8	0	0.751154	2.998681	0.177954
25	6	0	0.123696	4.236187	0.538399
26	1	0	0.792602	4.708328	1.252091
27	1	0	-0.013829	4.863236	-0.341880
28	1	0	-0.840988	4.016157	0.996533
29	6	0	3.304521	-0.785304	0.228867
30	6	0	2.362630	-0.300861	1.112715
31	1	0	4.137923	-0.164940	-0.087450
32	1	0	2.352447	0.757405	1.335770
33	1	0	1.902267	-0.975453	1.822136
34	8	0	3.515502	-2.137745	0.210919
35	6	0	4.678386	-2.544759	-0.480295
36	1	0	5.573639	-2.090793	-0.042285
37	1	0	4.740051	-3.626163	-0.381232
38	1	0	4.624555	-2.284699	-1.542681

TS26″ (NIMAG=1; 493.8i cm^{-1})

Center Number	Atomic Number	Atomic Type	Coordinates (Angstroms) X	Y	Z
1	6	0	-3.016204	1.160499	1.064906
2	6	0	-1.804354	1.805238	0.951420
3	6	0	-0.676483	1.038886	0.615850

4	1	0	-1.727414	2.885585	0.925423
5	16	0	0.217916	-1.488913	0.407509
6	8	0	-0.104058	-2.351180	-0.712848
7	8	0	0.166684	-1.988439	1.764816
8	6	0	1.896263	-0.915311	0.172164
9	6	0	2.550415	-0.295763	1.230199
10	6	0	2.537942	-1.193845	-1.028208
11	6	0	3.884319	0.066067	1.071814
12	1	0	2.030029	-0.123751	2.165307
13	6	0	3.875544	-0.842727	-1.167164
14	1	0	1.993148	-1.697728	-1.816963
15	6	0	4.545664	-0.214063	-0.120421
16	1	0	4.408316	0.551075	1.885950
17	1	0	4.397822	-1.068975	-2.088961
18	1	0	5.589870	0.052977	-0.232065
19	7	0	-0.888175	-0.239749	0.371252
20	1	0	-3.030188	0.122139	1.372831
21	1	0	-3.915701	1.744303	1.223806
22	6	0	0.535602	1.823376	0.188281
23	8	0	1.050311	2.690364	0.835610
24	8	0	0.901745	1.481765	-1.052917
25	6	0	2.074477	2.145795	-1.537442
26	1	0	2.286101	1.701782	-2.506210
27	1	0	1.886229	3.215674	-1.630651
28	1	0	2.902058	1.980358	-0.847437
29	6	0	-3.463528	0.127785	-0.874688
30	6	0	-2.225325	-0.318385	-1.284288
31	1	0	-3.869871	1.072602	-1.222845
32	1	0	-1.589060	0.350275	-1.847365
33	1	0	-2.056922	-1.381812	-1.386576
34	8	0	-4.363561	-0.801739	-0.451575
35	6	0	-5.670432	-0.312052	-0.228218
36	1	0	-6.069180	0.164427	-1.129916
37	1	0	-6.286722	-1.170854	0.027084
38	1	0	-5.690318	0.403631	0.599759

TS27″ (NIMAG=1; 533.2i cm^{-1})

Center Number	Atomic Number	Atomic Type	Coordinates (Angstroms) X	Y	Z
1	6	0	2.732202	0.304580	-1.556578
2	6	0	1.791855	1.314171	-1.419455
3	6	0	0.692429	1.083481	-0.591474
4	1	0	1.975178	2.323395	-1.765811
5	16	0	-0.531815	-0.381918	1.204258
6	8	0	-0.968989	0.845528	1.856125
7	8	0	-0.005172	-1.464942	2.010435
8	6	0	-1.877741	-1.027242	0.232705
9	6	0	-1.660333	-2.163403	-0.537379
10	6	0	-3.120931	-0.418425	0.317577
11	6	0	-2.722803	-2.688966	-1.260672
12	1	0	-0.677717	-2.618628	-0.565300
13	6	0	-4.178964	-0.961928	-0.402112
14	1	0	-3.242026	0.458303	0.942012
15	6	0	-3.977754	-2.089304	-1.191955
16	1	0	-2.573926	-3.570225	-1.872274
17	1	0	-5.159996	-0.506191	-0.344881
18	1	0	-4.804403	-2.506872	-1.754257
19	7	0	0.653838	-0.083307	0.043320
20	1	0	2.395508	-0.724094	-1.511884
21	1	0	3.620903	0.494974	-2.148116

22	6	0	-0.230803	2.257982	-0.345023
23	8	0	0.148220	3.340084	-0.001837
24	8	0	-1.491856	1.946953	-0.645516
25	6	0	-2.458383	2.932080	-0.271931
26	1	0	-2.224956	3.890230	-0.734822
27	1	0	-3.414859	2.554613	-0.625087
28	1	0	-2.454501	3.037303	0.814649
29	6	0	3.617725	-0.171403	0.291806
30	6	0	2.561796	-0.141978	1.179807
31	1	0	4.288093	0.679604	0.210558
32	1	0	2.284518	0.799688	1.633486
33	1	0	2.243327	-1.055579	1.662789
34	8	0	4.160019	-1.396218	-0.001694
35	6	0	5.436253	-1.344481	-0.605948
36	1	0	6.159335	-0.845613	0.047836
37	1	0	5.749071	-2.373764	-0.766623
38	1	0	5.403555	-0.825531	-1.569961

TS28″ (NIMAG=1; 502.6*i* cm^{-1})

Center Number	Atomic Number	Atomic Type	Coordinates (Angstroms) X	Y	Z
1	6	0	-2.675804	1.290968	1.142524
2	6	0	-1.485569	1.827036	0.695654
3	6	0	-0.512736	0.948039	0.192788
4	1	0	-1.358277	2.891482	0.541951
5	16	0	0.201001	-1.571460	-0.340913
6	8	0	0.167141	-1.682506	-1.785561
7	8	0	-0.177842	-2.707533	0.471996
8	6	0	1.835067	-1.060798	0.166468
9	6	0	2.099757	-0.922598	1.524504
10	6	0	2.805661	-0.850866	-0.803267
11	6	0	3.376187	-0.543365	1.918424
12	1	0	1.318778	-1.114763	2.251029
13	6	0	4.084649	-0.488665	-0.393076
14	1	0	2.541658	-0.956166	-1.848265
15	6	0	4.365156	-0.330093	0.960814
16	1	0	3.602561	-0.425362	2.970810
17	1	0	4.859918	-0.327382	-1.132362
18	1	0	5.362374	-0.043734	1.273654
19	7	0	-0.828406	-0.333377	0.188474
20	1	0	-2.669328	0.301137	1.581333
21	1	0	-3.478870	1.957211	1.436837
22	6	0	0.641398	1.550566	-0.564363
23	8	0	0.940996	1.259875	-1.688104
24	8	0	1.263995	2.474464	0.171160
25	6	0	2.378881	3.101752	-0.473063
26	1	0	2.773588	3.810848	0.248837
27	1	0	3.126687	2.350135	-0.727727
28	1	0	2.053917	3.609988	-1.381069
29	6	0	-3.636624	0.136176	-0.460154
30	6	0	-2.583578	-0.546464	-1.033758
31	1	0	-4.010989	1.057415	-0.896026
32	1	0	-2.036998	-0.110085	-1.857313
33	1	0	-2.565297	-1.625046	-0.935301
34	8	0	-4.523502	-0.583491	0.290220
35	6	0	-5.722436	0.095173	0.602783
36	1	0	-6.254508	0.389242	-0.308026
37	1	0	-6.337542	-0.600211	1.169291
38	1	0	-5.531203	0.984647	1.212285

TS29″ (NIMAG=1; 540.8*i* cm^{-1})

Center Number	Atomic Number	Atomic Type	Coordinates (Angstroms) X	Y	Z
1	6	0	-2.240452	-0.828307	1.977440
2	6	0	-1.810875	0.489706	1.892031
3	6	0	-0.913024	0.807856	0.874071
4	1	0	-2.298875	1.287934	2.438341
5	16	0	0.555258	0.046559	-1.157362
6	8	0	0.653727	1.442120	-1.556302
7	8	0	0.280168	-0.974644	-2.151527
8	6	0	2.053935	-0.393149	-0.300825
9	6	0	2.608294	-1.645920	-0.523206
10	6	0	2.623874	0.532006	0.567122
11	6	0	3.778958	-1.982023	0.147991
12	1	0	2.133665	-2.324621	-1.221146
13	6	0	3.789093	0.177040	1.234342
14	1	0	2.164497	1.503382	0.717016
15	6	0	4.363721	-1.074180	1.024619
16	1	0	4.234339	-2.950795	-0.016670
17	1	0	4.250911	0.880625	1.915833
18	1	0	5.275124	-1.341156	1.546192
19	7	0	-0.579559	-0.185846	0.055506
20	1	0	-1.526369	-1.603663	1.730156
21	1	0	-3.014454	-1.104098	2.683977
22	6	0	-0.630656	2.267545	0.589647
23	8	0	0.228478	2.909850	1.123955
24	8	0	-1.503147	2.739578	-0.298621
25	6	0	-1.178010	4.031973	-0.827612
26	1	0	-1.973007	4.271649	-1.527734
27	1	0	-1.128009	4.768552	-0.026463
28	1	0	-0.216799	3.966954	-1.338186
29	6	0	-3.161105	-1.464196	0.184877
30	6	0	-2.461699	-0.792079	-0.804874
31	1	0	-4.098118	-1.060312	0.544981
32	1	0	-2.735978	0.237399	-0.997320
33	1	0	-1.932575	-1.309480	-1.592954
34	8	0	-3.107174	-2.816290	0.363753
35	6	0	-2.015198	-3.476055	-0.258677
36	1	0	-1.074290	-2.949636	-0.066468
37	1	0	-1.979475	-4.475703	0.168224
38	1	0	-2.165361	-3.547259	-1.340282

TS30″ (NIMAG=1; 498.1*i* cm^{-1})

Center Number	Atomic Number	Atomic Type	Coordinates (Angstroms) X	Y	Z
1	6	0	-3.052124	1.543630	0.865027
2	6	0	-1.792786	2.109361	0.833881
3	6	0	-0.701931	1.258607	0.611225
4	1	0	-1.642904	3.180836	0.774337
5	16	0	-0.031118	-1.345162	0.564988
6	8	0	-0.505140	-2.308133	-0.413496
7	8	0	-0.079711	-1.653236	1.979240
8	6	0	1.682491	-1.004968	0.193594
9	6	0	2.483664	-0.451574	1.185228
10	6	0	2.189661	-1.361333	-1.049790
11	6	0	3.833926	-0.248777	0.917402
12	1	0	2.059902	-0.214608	2.154043

13	6	0	3.541134	-1.159161	-1.301844
14	1	0	1.533872	-1.811596	-1.784651
15	6	0	4.360342	-0.605971	-0.320283
16	1	0	4.474063	0.175130	1.681212
17	1	0	3.958561	-1.446699	-2.259286
18	1	0	5.415448	-0.459575	-0.519014
19	7	0	-0.993951	-0.009366	0.369835
20	1	0	-3.148159	0.534452	1.243026
21	1	0	-3.936984	2.167322	0.909484
22	6	0	0.594685	1.928712	0.242850
23	8	0	1.156507	2.749113	0.910852
24	8	0	0.987155	1.546187	-0.979487
25	6	0	2.223200	2.117240	-1.422684
26	1	0	2.436094	1.651618	-2.381015
27	1	0	2.119271	3.197598	-1.527430
28	1	0	3.010904	1.895888	-0.702845
29	6	0	-3.443575	0.463251	-1.023560
30	6	0	-2.187338	0.001349	-1.376940
31	1	0	-3.811374	1.404194	-1.410322
32	1	0	-1.526761	0.694976	-1.880525
33	1	0	-1.984696	-1.051443	-1.515131
34	8	0	-4.460413	-0.332676	-0.609788
35	6	0	-4.100988	-1.651647	-0.215970
36	1	0	-3.260019	-1.639733	0.483513
37	1	0	-4.980943	-2.073713	0.263023
38	1	0	-3.830091	-2.258853	-1.084442

TS31″ (NIMAG=1; 545.5*i* cm^{-1})

Center Number	Atomic Number	Atomic Type	Coordinates (Angstroms) X	Y	Z
1	6	0	2.765433	0.500265	-1.736801
2	6	0	1.801767	1.483679	-1.536313
3	6	0	0.749248	1.194731	-0.671813
4	1	0	1.944029	2.507410	-1.858514
5	16	0	-0.306831	-0.415347	1.129076
6	8	0	-0.709045	0.735541	1.925795
7	8	0	0.300147	-1.558809	1.787453
8	6	0	-1.717456	-1.004873	0.214581
9	6	0	-1.543254	-2.038353	-0.697335
10	6	0	-2.964077	-0.460110	0.484158
11	6	0	-2.654902	-2.522868	-1.374109
12	1	0	-0.555365	-2.447137	-0.872167
13	6	0	-4.070593	-0.962666	-0.190752
14	1	0	-3.049331	0.338192	1.211502
15	6	0	-3.914335	-1.986278	-1.119937
16	1	0	-2.540182	-3.322980	-2.094858
17	1	0	-5.054278	-0.555588	0.009375
18	1	0	-4.778978	-2.371485	-1.646996
19	7	0	0.789148	0.004385	-0.072659
20	1	0	2.431529	-0.530589	-1.740724
21	1	0	3.635356	0.713164	-2.347042
22	6	0	-0.185034	2.326961	-0.310067
23	8	0	0.188724	3.414174	0.024444
24	8	0	-1.458877	1.973638	-0.478854
25	6	0	-2.416844	2.910312	0.020831
26	1	0	-2.263727	3.889490	-0.431291
27	1	0	-3.390004	2.507288	-0.248449
28	1	0	-2.308825	2.983242	1.104594
29	6	0	3.717370	-0.016834	0.026890
30	6	0	2.724134	0.095988	0.987217

31	1	0	4.411227	0.802060	-0.113705
32	1	0	2.491518	1.089158	1.349899
33	1	0	2.420759	-0.736256	1.606234
34	8	0	4.280831	-1.206864	-0.335868
35	6	0	3.551324	-2.368424	0.031731
36	1	0	2.488270	-2.260235	-0.208276
37	1	0	3.983631	-3.191603	-0.532451
38	1	0	3.648716	-2.569898	1.102818

TS32″ (NIMAG=1; 540.7*i* cm^{-1})

Center Number	Atomic Number	Atomic Type	Coordinates (Angstroms) X	Y	Z
1	6	0	-2.240965	-0.826606	1.977688
2	6	0	-1.809976	0.490939	1.892336
3	6	0	-0.911919	0.808190	0.874281
4	1	0	-2.297088	1.289657	2.438723
5	16	0	0.555136	0.045755	-1.157483
6	8	0	0.654278	1.441182	-1.556743
7	8	0	0.279322	-0.975490	-2.151404
8	6	0	2.053683	-0.394547	-0.301027
9	6	0	2.606572	-1.648209	-0.522105
10	6	0	2.624888	0.530954	0.565707
11	6	0	3.777071	-1.984828	0.149099
12	1	0	2.130997	-2.327191	-1.219126
13	6	0	3.789942	0.175468	1.232960
14	1	0	2.166574	1.502963	0.714702
15	6	0	4.363127	-1.076612	1.024489
16	1	0	4.231326	-2.954295	-0.014581
17	1	0	4.252736	0.879321	1.913510
18	1	0	5.274404	-1.343992	1.546075
19	7	0	-0.579593	-0.185833	0.055637
20	1	0	-1.527781	-1.602730	1.730222
21	1	0	-3.015212	-1.101627	2.684257
22	6	0	-0.628333	2.267621	0.589627
23	8	0	0.231645	2.909189	1.123462
24	8	0	-1.500943	2.740411	-0.298122
25	6	0	-1.174958	4.032496	-0.827348
26	1	0	-1.969825	4.272595	-1.527473
27	1	0	-1.124437	4.769172	-0.026318
28	1	0	-0.213813	3.966752	-1.337949
29	6	0	-3.162523	-1.461295	0.185007
30	6	0	-2.462389	-0.789871	-0.804702
31	1	0	-4.099032	-1.056372	0.545249
32	1	0	-2.735598	0.239903	-0.997104
33	1	0	-1.933914	-1.307859	-1.592838
34	8	0	-3.110097	-2.813461	0.363754
35	6	0	-2.018803	-3.474347	-0.258690
36	1	0	-1.077353	-2.948899	-0.066477
37	1	0	-1.984121	-4.474044	0.168182
38	1	0	-2.169034	-3.545360	-1.340298
